# Supplementary material for: Sulfated Triterpene Glycosides from the Far Eastern Sea Cucumber Cucumaria djakonovi: Djakonoviosides C1, D1, E1, and F1; Cytotoxicity against Human Breast Cancer Cell Lines; Quantitative Structure–Activity Relationships
Source: Mar Drugs. 2023 Nov 22;21(12):602. doi: 10.3390/md21120602 (PMC10744391; doi:10.3390/md21120602)
Supplement: Supplementary file 1 [file marinedrugs-21-00602-s001.zip › marinedrugs-2726444-supplementary.pdf]

Supplementary data content page

Title: Sulfated Triterpene Glycosides from the Far Eastern Sea Cucumber *Cucumaria djakonovi*: Djakonoviosides C_1, D_1, E_1, and F_1; Cytotoxicity Against Human Breast Cancer Cell Lines; Quantitative Structure – Activity Relationships

Authors: Alexandra S. Silchenko[1*], Anatoly I. Kalinovsky[1], Sergey A. Avilov[1], Roman S. Popov[1], Ekaterina A. Chingizova[1], Ekaterina S. Menchinskaya[1], Elena G. Panina[2], Vadim G. Stepanov[2], Elena A. Zelepuga[1], Vladimir I. Kalinin[1], Pavel. S. Dmitrenok[1*]

Address: [1]*G.B. Elyakov Pacific Institute of Bioorganic Chemistry, Far Eastern Branch of Russian Academy of Sciences, Pr. 100-let Vladivostoka 159, 690022 Vladivostok, Russia*
[2] *Kamchatka Branch of Pacific Institute of Geography, Far Eastern Branch of the Russian Academy of Sciences, Partizanskaya st. 6, 683000, Petropavlovsk-Kamchatsky, Russia;*
Correspondence: silchenko_als@piboc.dvo.ru; paveldmt@piboc.dvo.ru Tel.: +7-423-231-1168

Content:

Figure S1. The ^{13}C NMR (125.67 MHz) spectrum of djakonovioside C_1 (**1**) in C_5D_5N/D_2O (4/1)
Figure S2. The ^{1}H NMR (500.12 MHz) spectrum of djakonovioside C_1 (**1**) in C_5D_5N/D_2O (4/1)
Figure S3. The COSY (500.12 MHz) spectrum of djakonovioside C_1 (**1**) in C_5D_5N/D_2O (4/1)
Figure S4. The HSQC (500.12 MHz) spectrum of djakonovioside C_1 (**1**) in C_5D_5N/D_2O (4/1)
Figure S5. The ROESY (500.12 MHz) spectrum of djakonovioside C_1 (**1**) in C_5D_5N/D_2O (4/1)
Figure S6. The HMBC (500.12 MHz) spectrum of djakonovioside C_1 (**1**) in C_5D_5N/D_2O (4/1)
Figure S7. 1 D TOCSY (500.12 MHz) spectra of Xyl1, Xyl2, Glc3, MeGlc4, Xyl5 of djakonovioside C_1 (**1**) in C_5D_5N/D_2O (4/1)
Figure S8. HR-ESI-MS and ESI-MS/MS spectra of djakonovioside C_1 (**1**)
Figure S9. The ^{13}C NMR (125.67 MHz) spectrum of djakonovioside D_1 (**2**) in C_5D_5N/D_2O (4/1)
Figure S10. The ^{1}H NMR (500.12 MHz) spectrum of djakonovioside D_1 (**2**) in C_5D_5N/D_2O (4/1)
Figure S11. The COSY (500.12 MHz) spectrum of djakonovioside D_1 (**2**) in C_5D_5N/D_2O (4/1)
Figure S12. The HSQC (500.12 MHz) spectrum of djakonovioside D_1 (**2**) in C_5D_5N/D_2O (4/1)
Figure S13. The HMBC (500.12 MHz) spectrum of djakonovioside D_1 (**2**) in C_5D_5N/D_2O (4/1)
Figure S14. The ROESY (500.12 MHz) spectrum of djakonovioside D_1 (**2**) in C_5D_5N/D_2O (4/1)
Figure S15. 1D TOCSY (500.12 MHz) spectra of Xyl1, Glc2, Glc3, MeGlc4, Xyl5 of djakonovioside D_1 (**2**) in C_5D_5N/D_2O (4/1)
Figure S16. HR-ESI-MS and ESI-MS/MS spectra of djakonovioside D_1 (**2**)
Figure S17. The ^{13}C NMR (125.67 MHz) spectrum of djakonovioside E_1 (**3**) in C_5D_5N/D_2O (4/1)
Figure S18. The ^{1}H NMR (500.12 MHz) spectrum of djakonovioside E_1 (**3**) in C_5D_5N/D_2O (4/1)
Figure S19. The COSY (500.12 MHz) spectrum of djakonovioside E_1 (**3**) in C_5D_5N/D_2O (4/1)
Figure S20. The HSQC (500.12 MHz) spectrum of djakonovioside E_1 (**3**) in C_5D_5N/D_2O (4/1)
Figure S21. The ROESY (500.12 MHz) spectrum of djakonovioside E_1 (**3**) in C_5D_5N/D_2O (4/1)
Figure S22. The HMBC (500.12 MHz) spectrum of djakonovioside E_1 (**3**) in C_5D_5N/D_2O (4/1)

Figure S23. 1D TOCSY (500.12 MHz) spectra of Xyl1, Glc2, Glc3, MeGlc4 of djakonovioside E_1 (**3**) in C_5D_5N/D_2O (4/1)
Figure S24. HR-ESI-MS and ESI-MS/MS spectra of djakonovioside E_1 (**3**)
Table S1. ^{13}C and ^{1}H NMR chemical shifts, HMBC and ROESY correlations of the aglycone part of djakonovioside E_1 (**3**)
Figure S25. The ^{13}C NMR (125.67 MHz) spectrum of djakonovioside F_1 (**4**) in C_5D_5N/D_2O (4/1)
Figure S26. The ^{1}H NMR (500.12 MHz) spectrum of djakonovioside F_1 (**4**) in C_5D_5N/D_2O (4/1)
Figure S27. The COSY (500.12 MHz) spectrum of djakonovioside F_1 (**4**) in C_5D_5N/D_2O (4/1)
Figure S28. The HSQC (500.12 MHz) spectrum of djakonovioside F_1 (**4**) in C_5D_5N/D_2O (4/1)
Figure S29. The ROESY (500.12 MHz) spectrum of djakonovioside F_1 (**4**) in C_5D_5N/D_2O (4/1)
Figure S30. The HMBC (500.12 MHz) spectrum of djakonovioside F_1 (**4**) in C_5D_5N/D_2O (4/1)
Figure S31. 1D TOCSY (500.12 MHz) spectra of Xyl1, Qui2, Glc3, MeGlc4, Xyl5 of djakonovioside F_1 (**4**) in C_5D_5N/D_2O (4/1)
Figure S32. HR-ESI-MS and ESI-MS/MS spectra of djakonovioside F_1 (**4**)
Table S2. ^{13}C and ^{1}H NMR chemical shifts, HMBC and ROESY correlations of the aglycone part of okhotoside A_2-1 (**5**)
Table S3. ^{13}C and ^{1}H NMR chemical shifts, HMBC and ROESY correlations of the carbohydrate part of okhotoside A_2-1 (**5**)
Figure S33. The ^{13}C NMR (125.67 MHz) spectrum of okhotoside A_2-1 (**5**) in C_5D_5N/D_2O (4/1)
Figure S34. The ^{1}H NMR (500.12 MHz) spectrum of okhotoside A_2-1 (**5**) in C_5D_5N/D_2O (4/1)
Figure S35. The COSY (500.12 MHz) spectrum of okhotoside A_2-1 (**5**) in C_5D_5N/D_2O (4/1)
Figure S36. The HSQC (500.12 MHz) spectrum of okhotoside A_2-1 (**5**) in C_5D_5N/D_2O (4/1)
Figure S37. The ROESY (500.12 MHz) spectrum of okhotoside A_2-1 (**5**) in C_5D_5N/D_2O (4/1)
Figure S38. The HMBC (500.12 MHz) spectrum of okhotoside A_2-1 (**5**) in C_5D_5N/D_2O (4/1)
Table S4. ^{13}C and ^{1}H NMR chemical shifts, HMBC and ROESY correlations of the aglycone part of cucumarioside A_2-5 (**6**)
Table S5. ^{13}C and ^{1}H NMR chemical shifts, HMBC and ROESY correlations of the carbohydrate part of cucumarioside A_2-5 (**6**)
Figure S39. The ^{13}C NMR (125.67 MHz) spectrum of cucumarioside A_2-5 (**6**) in C_5D_5N/D_2O (4/1)
Figure S40. The ^{1}H NMR (500.12 MHz) spectrum of cucumarioside A_2-5 (**6**) in C_5D_5N/D_2O (4/1)
Figure S41. The COSY (500.12 MHz) spectrum of cucumarioside A_2-5 (**6**) in C_5D_5N/D_2O (4/1)
Figure S42. The HSQC (500.12 MHz) spectrum of cucumarioside A_2-5 (**6**) in C_5D_5N/D_2O (4/1)
Figure S43. The ROESY (500.12 MHz) spectrum of cucumarioside A_2-5 (**6**) in C_5D_5N/D_2O (4/1)
Figure S44. The HMBC (500.12 MHz) spectrum of cucumarioside A_2-5 (**6**) in C_5D_5N/D_2O (4/1)
Table S6. ^{13}C NMR chemical shifts of frondoside A_2-3 (**7**).
Figure S45. The ^{13}C NMR (125.67 MHz) spectrum of cucumarioside A_3-2 (**8**) in C_5D_5N/D_2O (4/1)
Figure S46. The ^{1}H NMR (500.12 MHz) spectrum of cucumarioside A_3-2 (**8**) in C_5D_5N/D_2O (4/1)
Figure S47. The COSY (500.12 MHz) spectrum of cucumarioside A_3-2 (**8**) in C_5D_5N/D_2O (4/1)

Figure S48. The HSQC (500.12 MHz) spectrum of cucumarioside A_3-2 (**8**) in C_5D_5N/D_2O (4/1)

Figure S49. The ROESY (500.12 MHz) spectrum of cucumarioside A_3-2 (**8**) in C_5D_5N/D_2O (4/1)

Figure S50. The HMBC (500.12 MHz) spectrum of cucumarioside A_3-2 (**8**) in C_5D_5N/D_2O (4/1)

Figure S51. 1D TOCSY (500.12 MHz) spectra of Xyl1, Qui2, Glc3, MeGlc4, Xyl5 of cucumarioside A_3-2 (**8**) in C_5D_5N/D_2O (4/1)

Table S7. ^{13}C and ^{1}H NMR chemical shifts, HMBC and ROESY correlations of the carbohydrate part of isokoreoside A (**9**)

Table S8. ^{13}C and ^{1}H NMR chemical shifts, HMBC and ROESY correlations of the aglycone part of isokoreoside A (**9**)

Figure S52. The ^{13}C NMR (125.67 MHz) spectrum of isokoreoside A (**9**) in C_5D_5N/D_2O (4/1)

Figure S53. The ^{1}H NMR (500.12 MHz) spectrum of isokoreoside A (**9**) in C_5D_5N/D_2O (4/1)

Figure S54. The COSY (500.12 MHz) spectrum of isokoreoside A (**9**) in C_5D_5N/D_2O (4/1)

Figure S55. The HSQC (500.12 MHz) spectrum of isokoreoside A (**9**) in C_5D_5N/D_2O (4/1)

Figure S56. The ROESY (500.12 MHz) spectrum of isokoreoside A (**9**) in C_5D_5N/D_2O (4/1)

Figure S57. The HMBC (500.12 MHz) spectrum of isokoreoside A (**9**) in C_5D_5N/D_2O (4/1)

Table S9. ^{13}C NMR chemical shifts of koreoside A (**10**)

Figure S58. The PCR QSAR model correlation plot reflecting the relationship of predicted and experimental hemolytic activity.

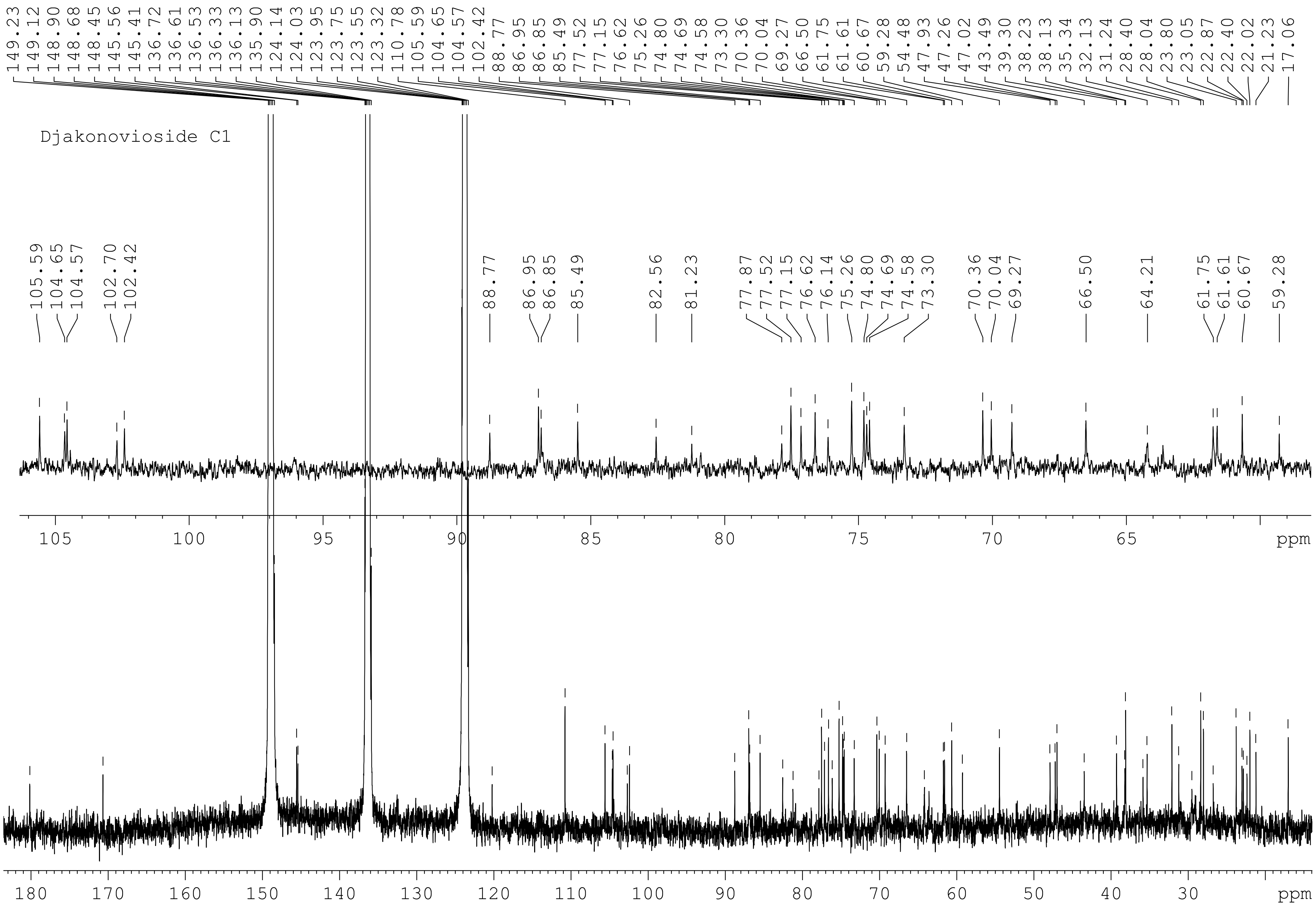

Figure S1. The ^{13}C NMR (125.67 MHz) spectrum of djakonovioside C_1 (**1**) in C_5D_5N/D_2O (4/1)

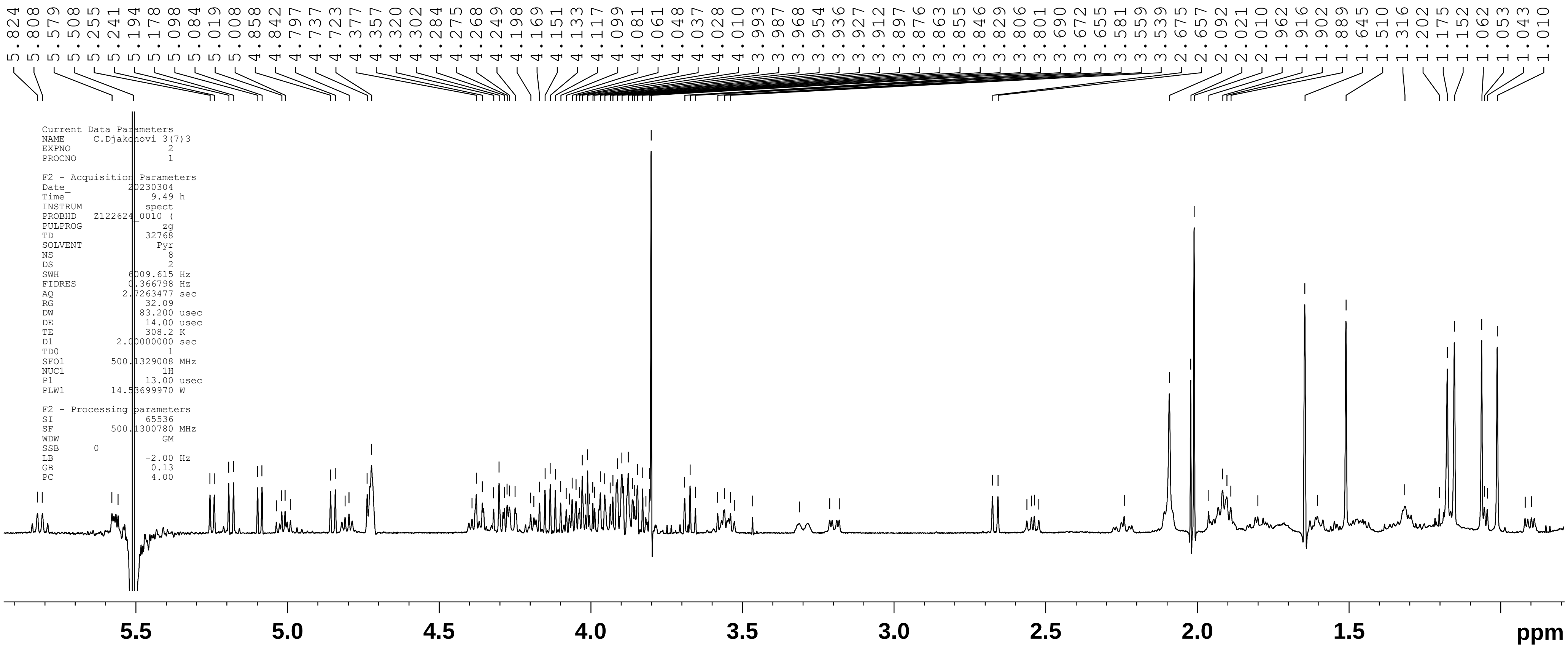

Figure S2. The ^{1}H NMR (500.12 MHz) spectrum of djakonovioside C$_1$ (**1**) in C_5D_5N/D_2O (4/1)

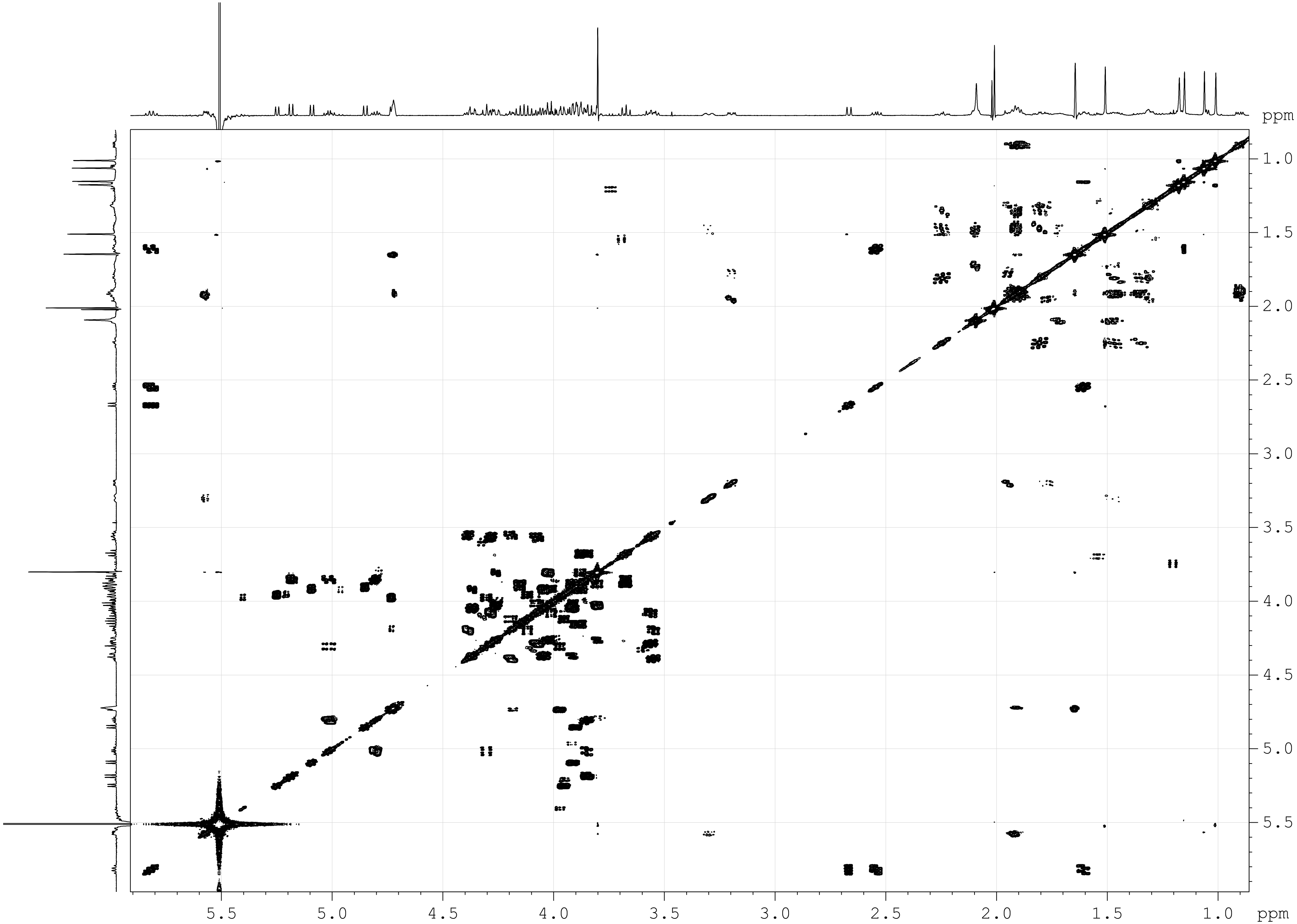

Figure S3. The COSY (500.12 MHz) spectrum of djakonovioside C1 (**1**) in C_5D_5N/D_2O (4/1)

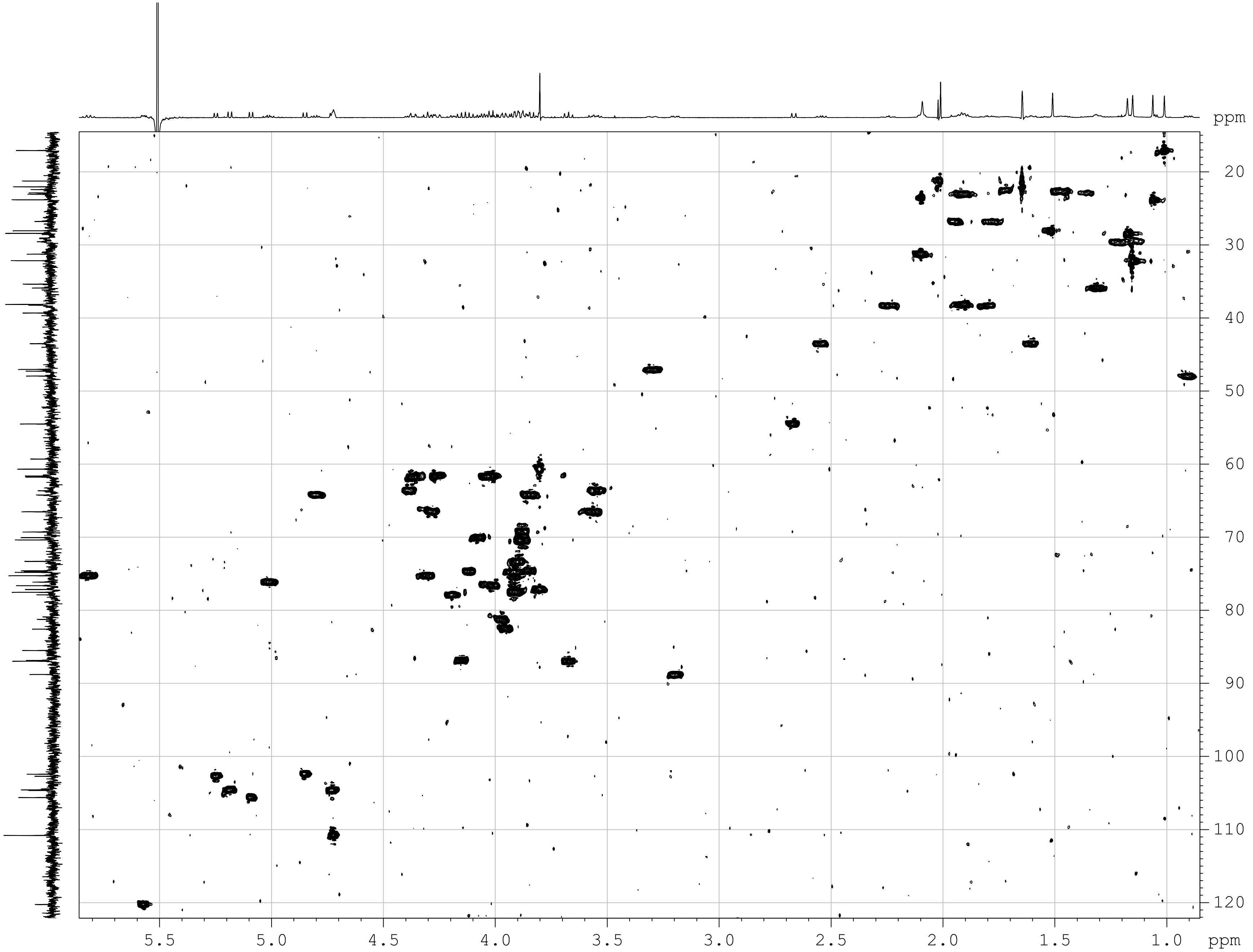

Figure S4. The HSQC (500.12 MHz) spectrum of djakonovioside C_1 (**1**) in C_5D_5N/D_2O (4/1)

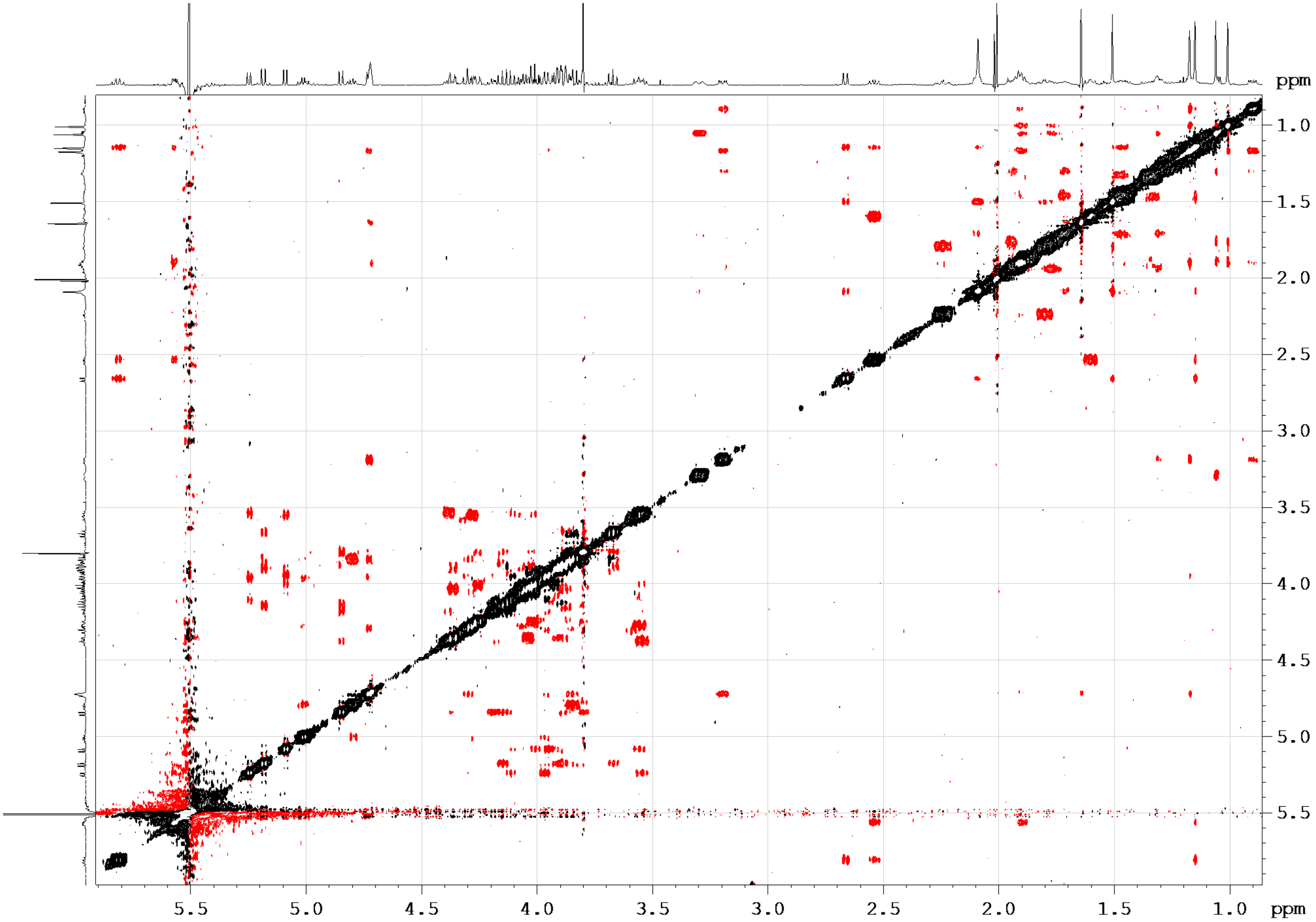

Figure S5. The ROESY (500.12 MHz) spectrum of djakonovioside C_1 (**1**) in C_5D_5N/D_2O (4/1)

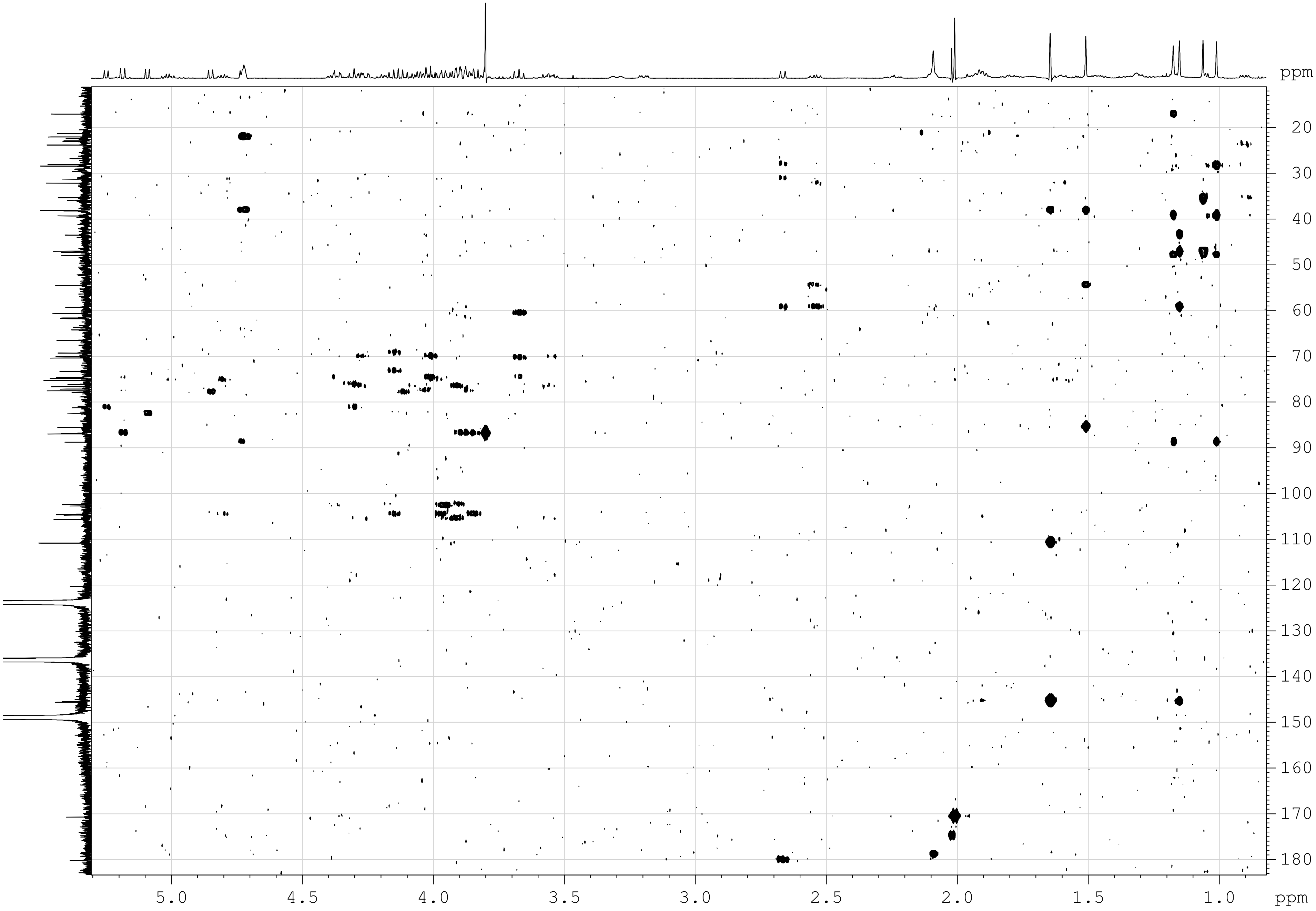

Figure S6. The HMBC (500.12 MHz) spectrum of djakonovioside C_1 (**1**) in C_5D_5N/D_2O (4/1)

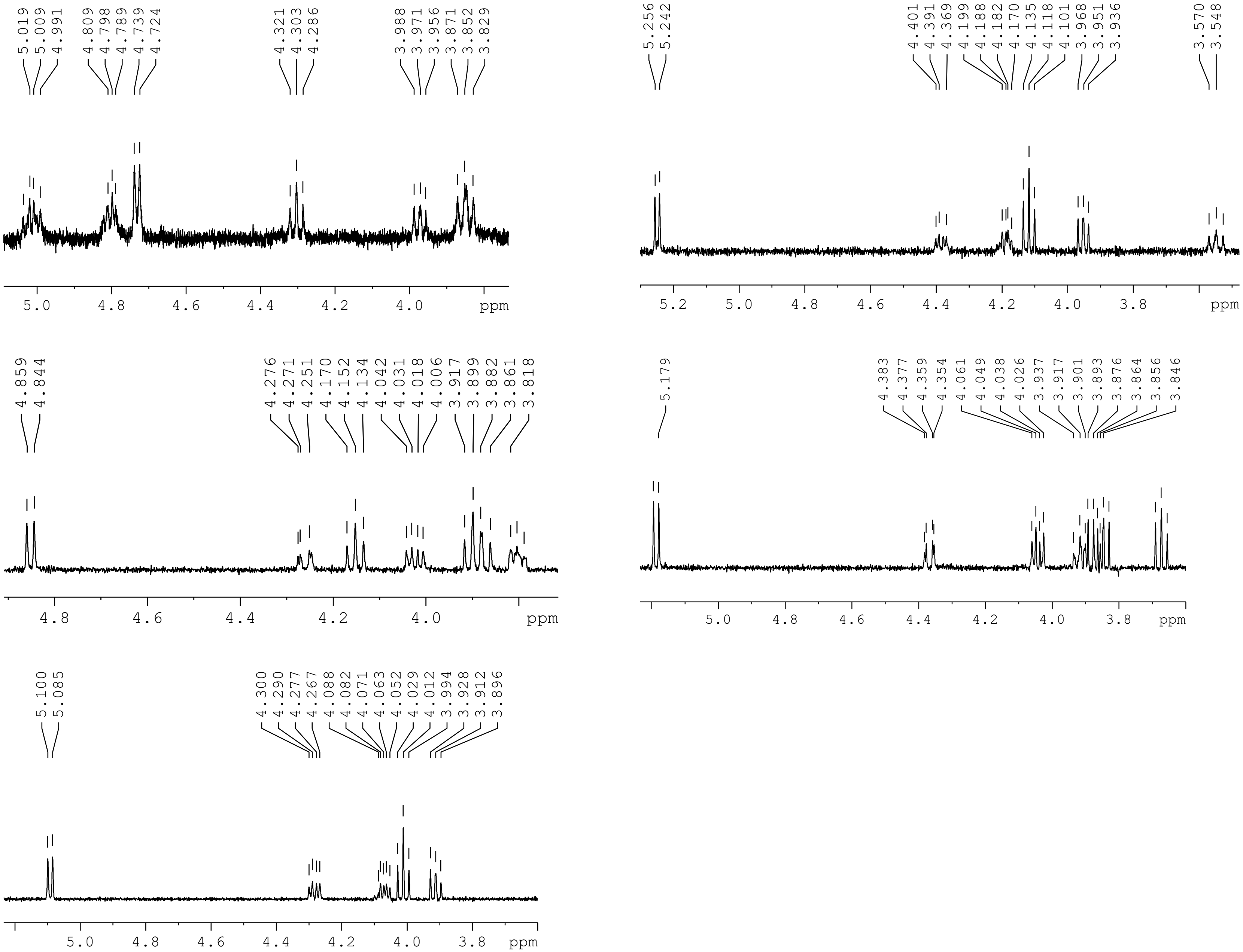

Figure S7. 1 D TOCSY (500.12 MHz) spectra of Xyl1, Xyl2, Glc3, MeGlc4, Xyl5 of djakonovioside C_1 (**1**) in C_5D_5N/D_2O (4/1)

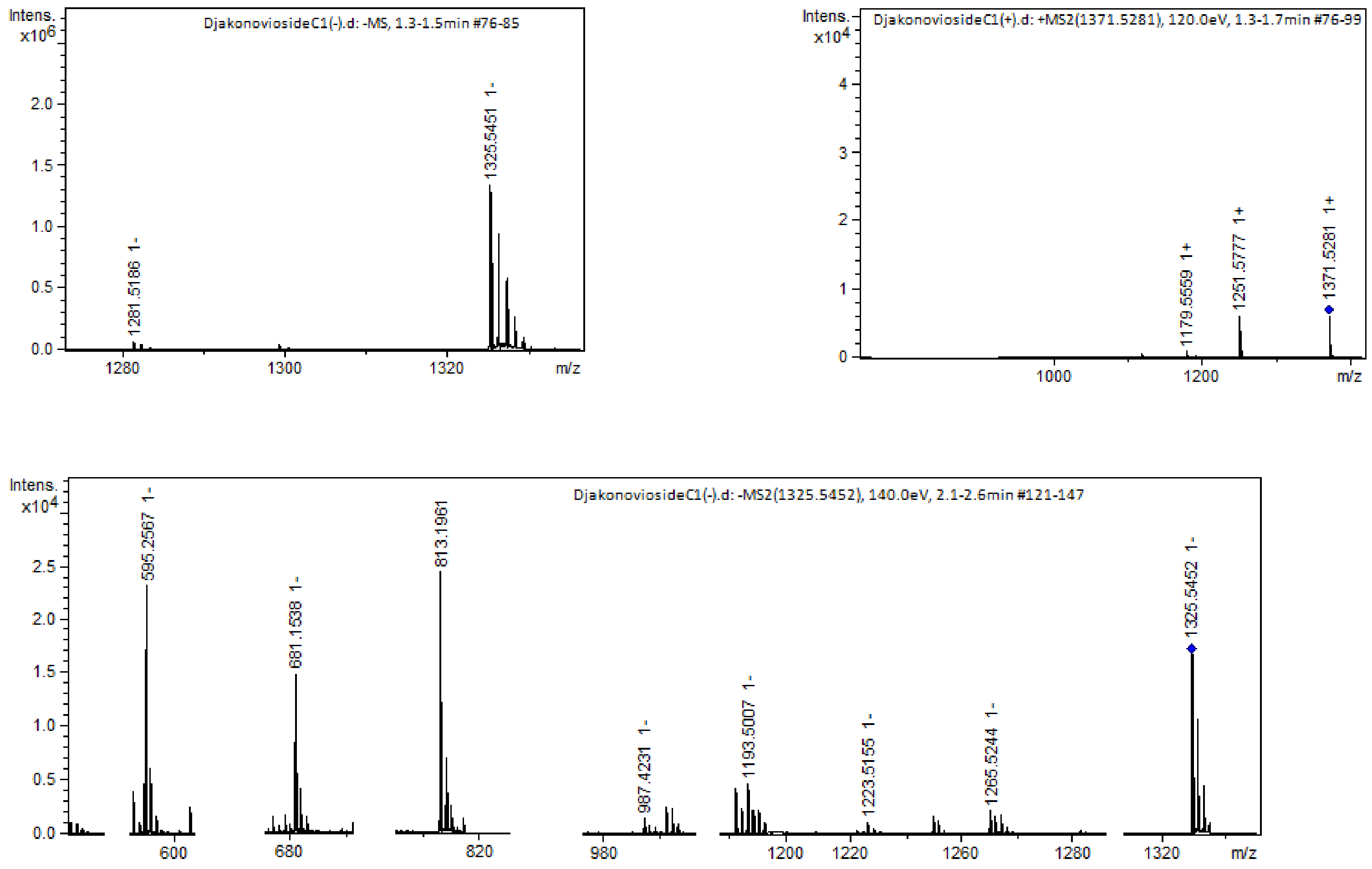

Figure S8. HR-ESI-MS and ESI-MS/MS spectra of djakonovioside C_1 (**1**)

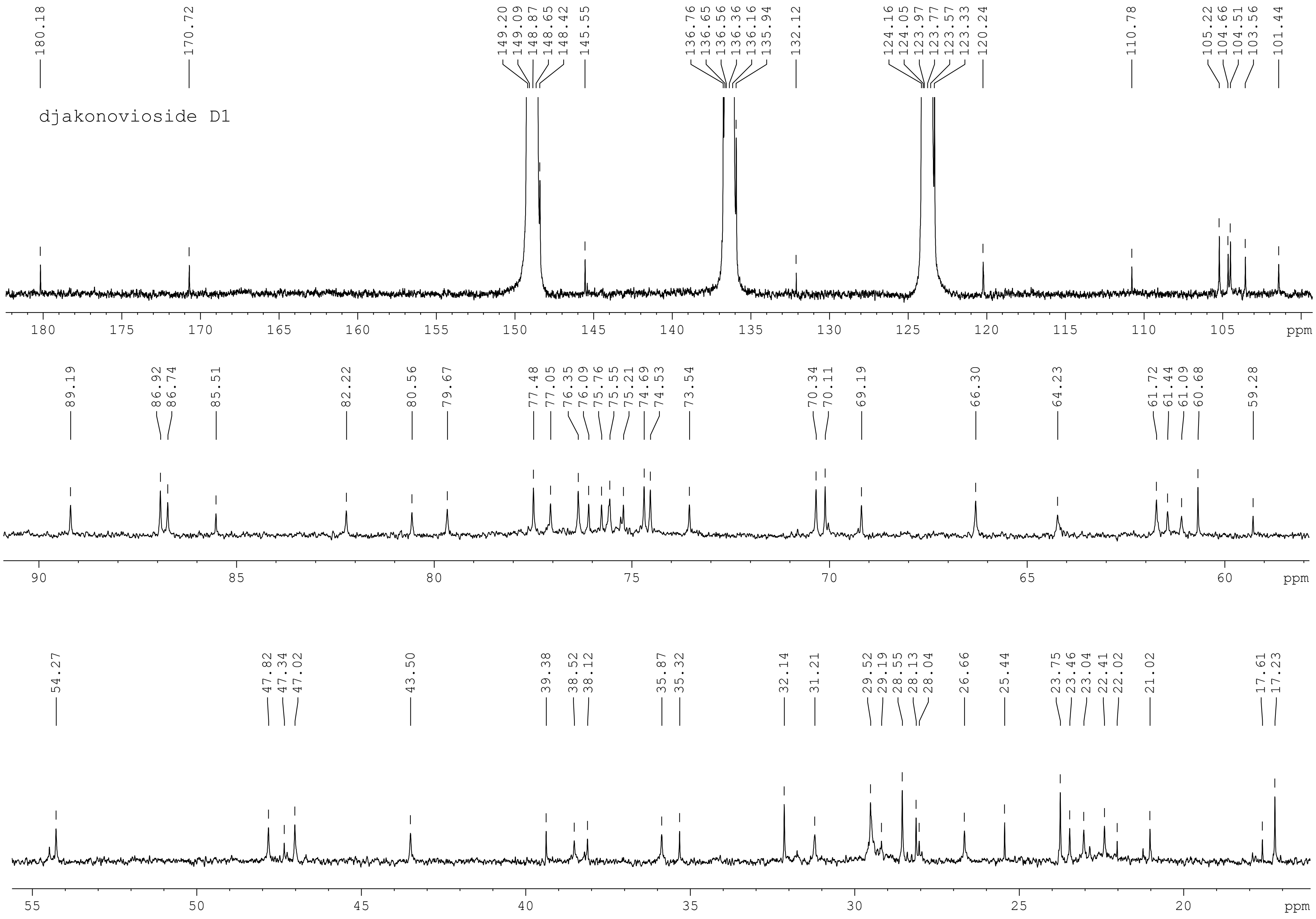

Figure S9. The ^{13}C NMR (125.67 MHz) spectrum of djakonovioside D_1 (**2**) in C_5D_5N/D_2O (4/1)

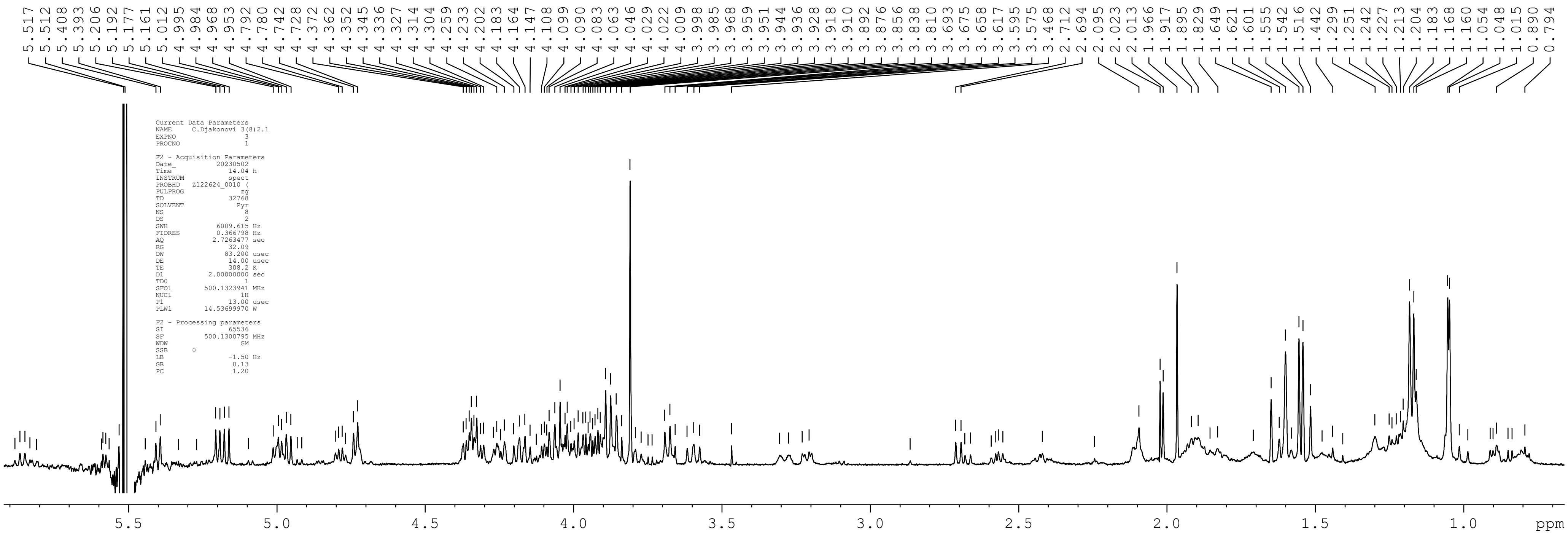

Figure S10. The ^{1}H NMR (500.12 MHz) spectrum of djakonovioside D$_1$ (**2**) in C_5D_5N/D_2O (4/1)

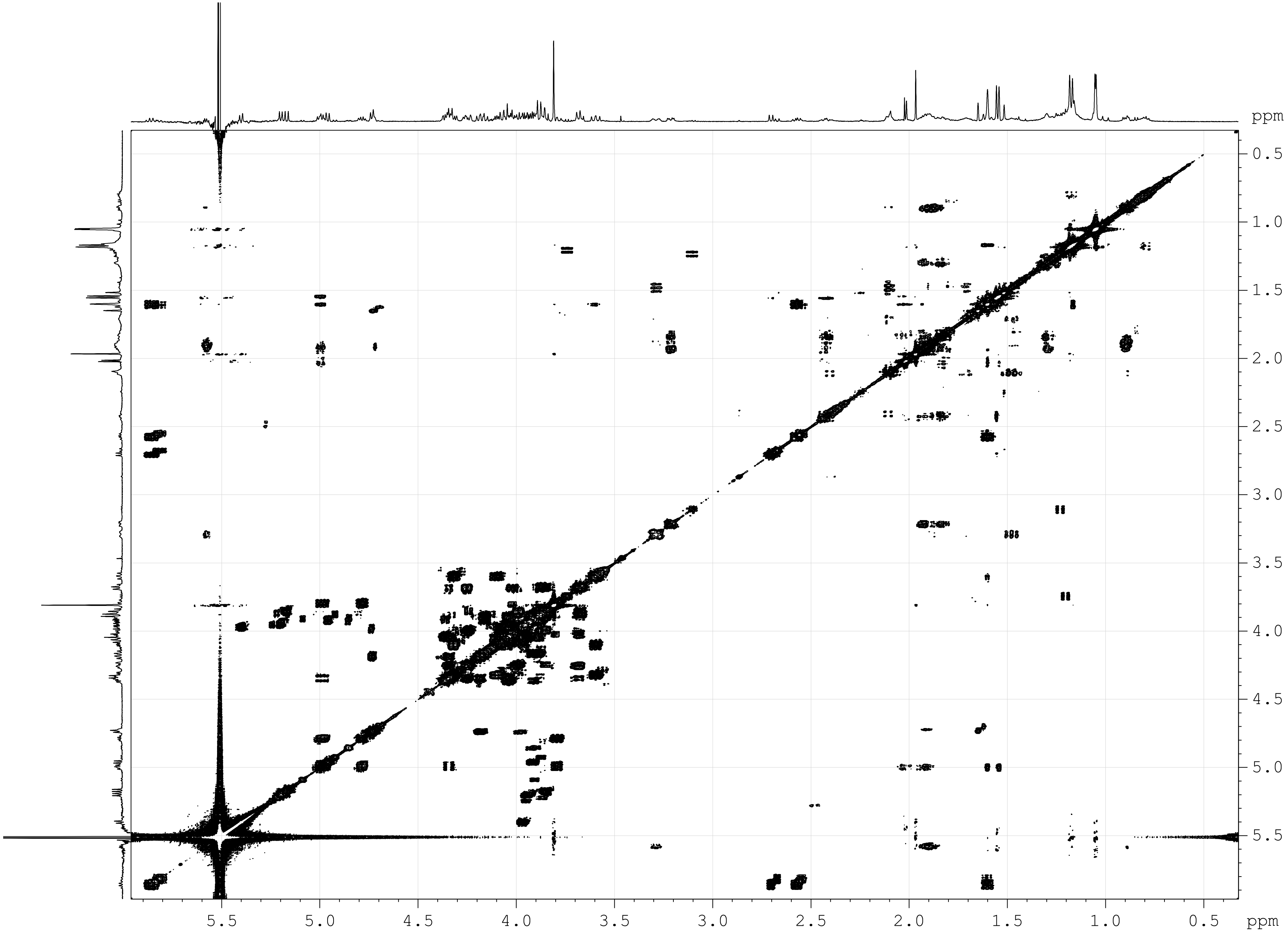

Figure S11. The COSY (500.12 MHz) spectrum of djakonovioside D_1 (**2**) in C_5D_5N/D_2O (4/1)

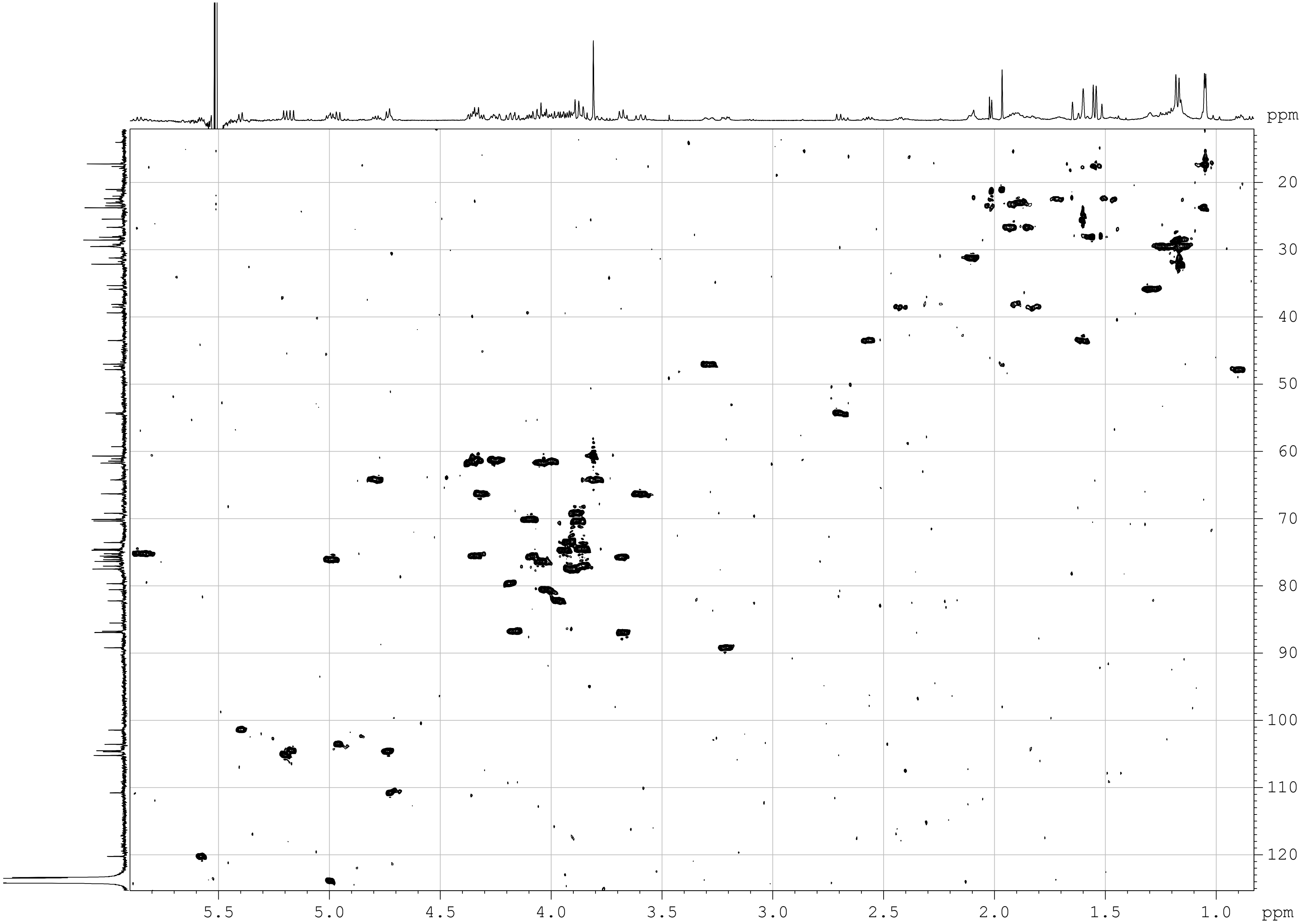

Figure S12. The HSQC (500.12 MHz) spectrum of djakonovioside D_1 (**2**) in C_5D_5N/D_2O (4/1)

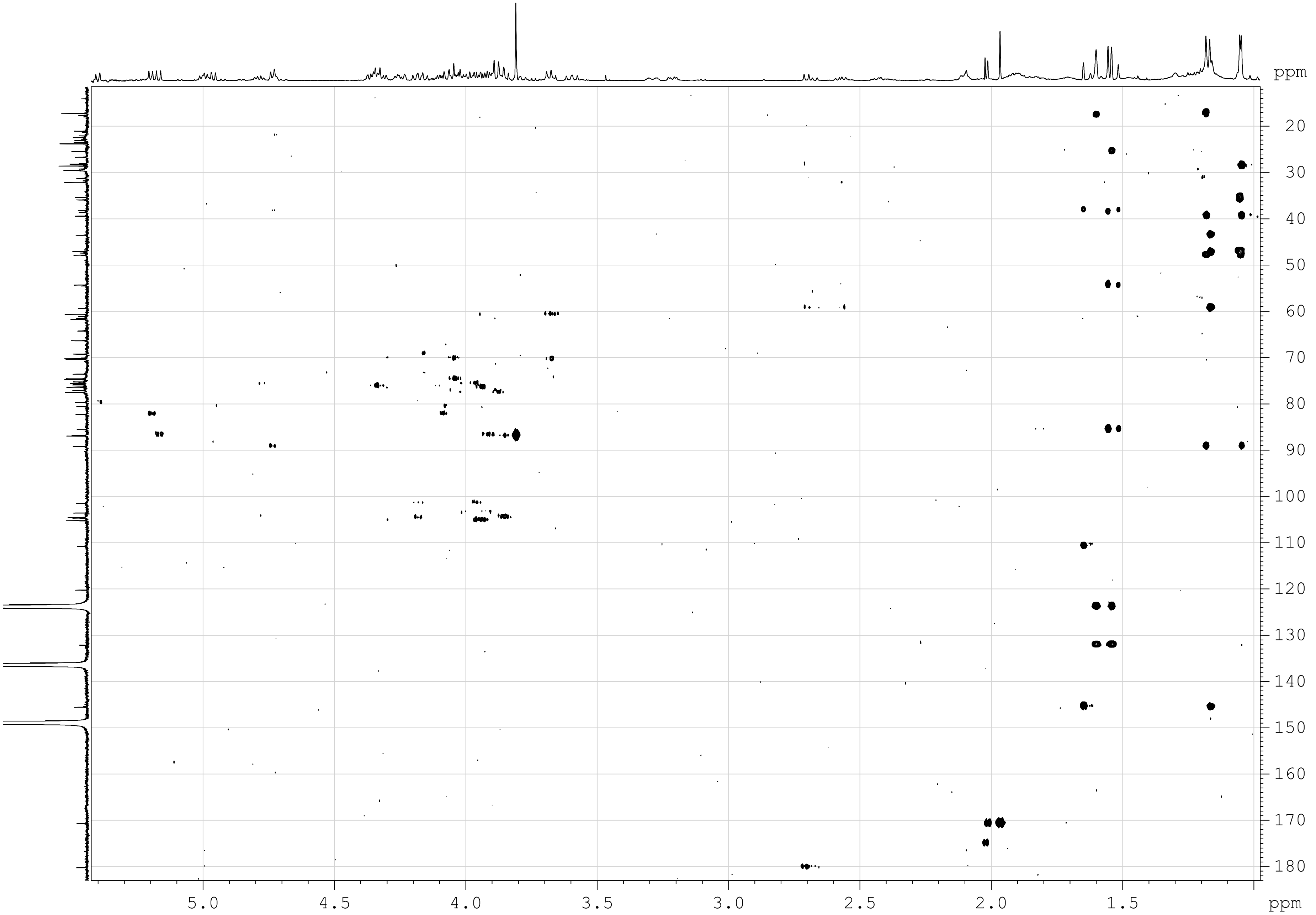

Figure S13. The HMBC (500.12 MHz) spectrum of djakonovioside D_1 (**2**) in C_5D_5N/D_2O (4/1)

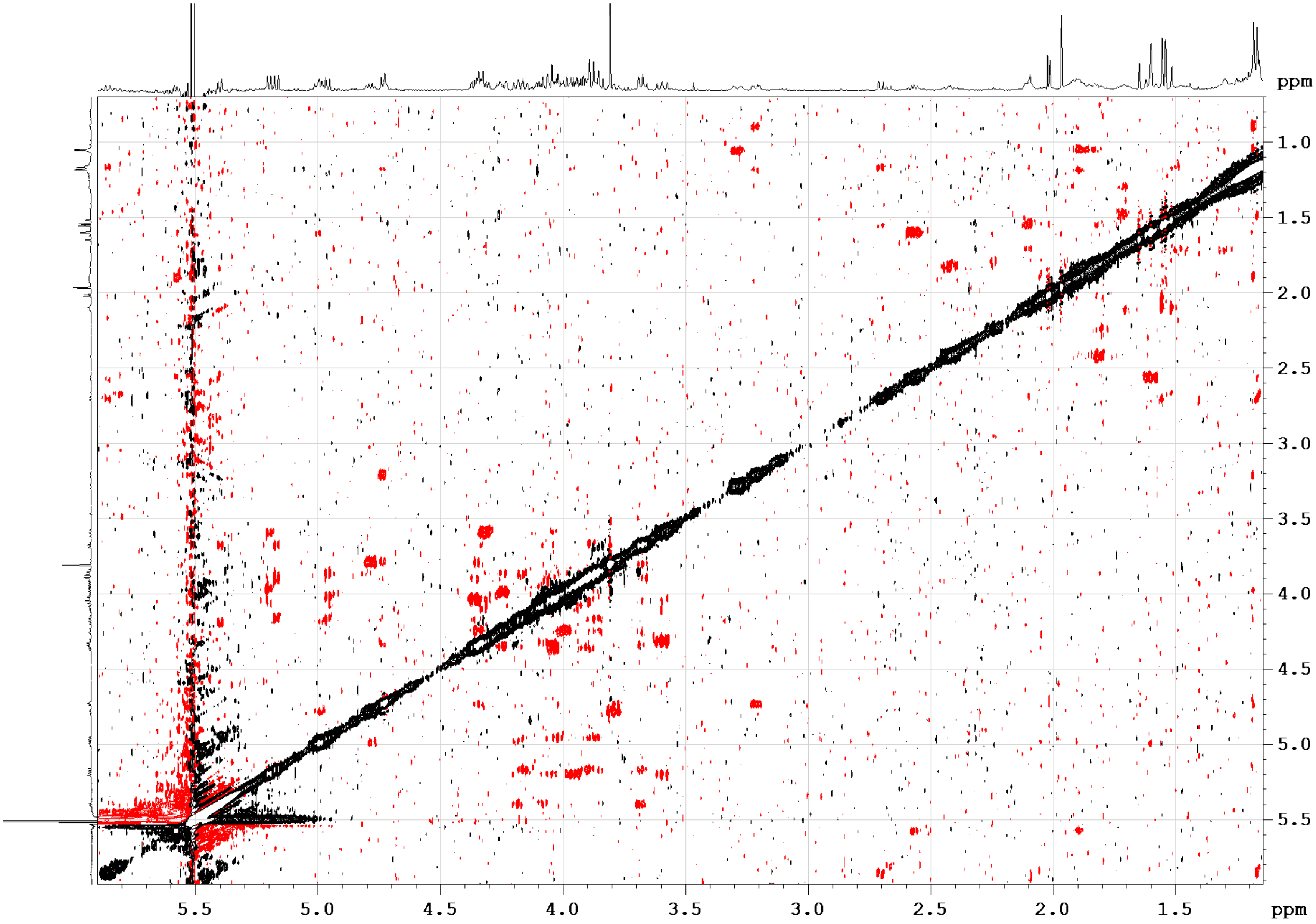

Figure S14. The ROESY (500.12 MHz) spectrum of djakonovioside D_1 (**2**) in C_5D_5N/D_2O (4/1)

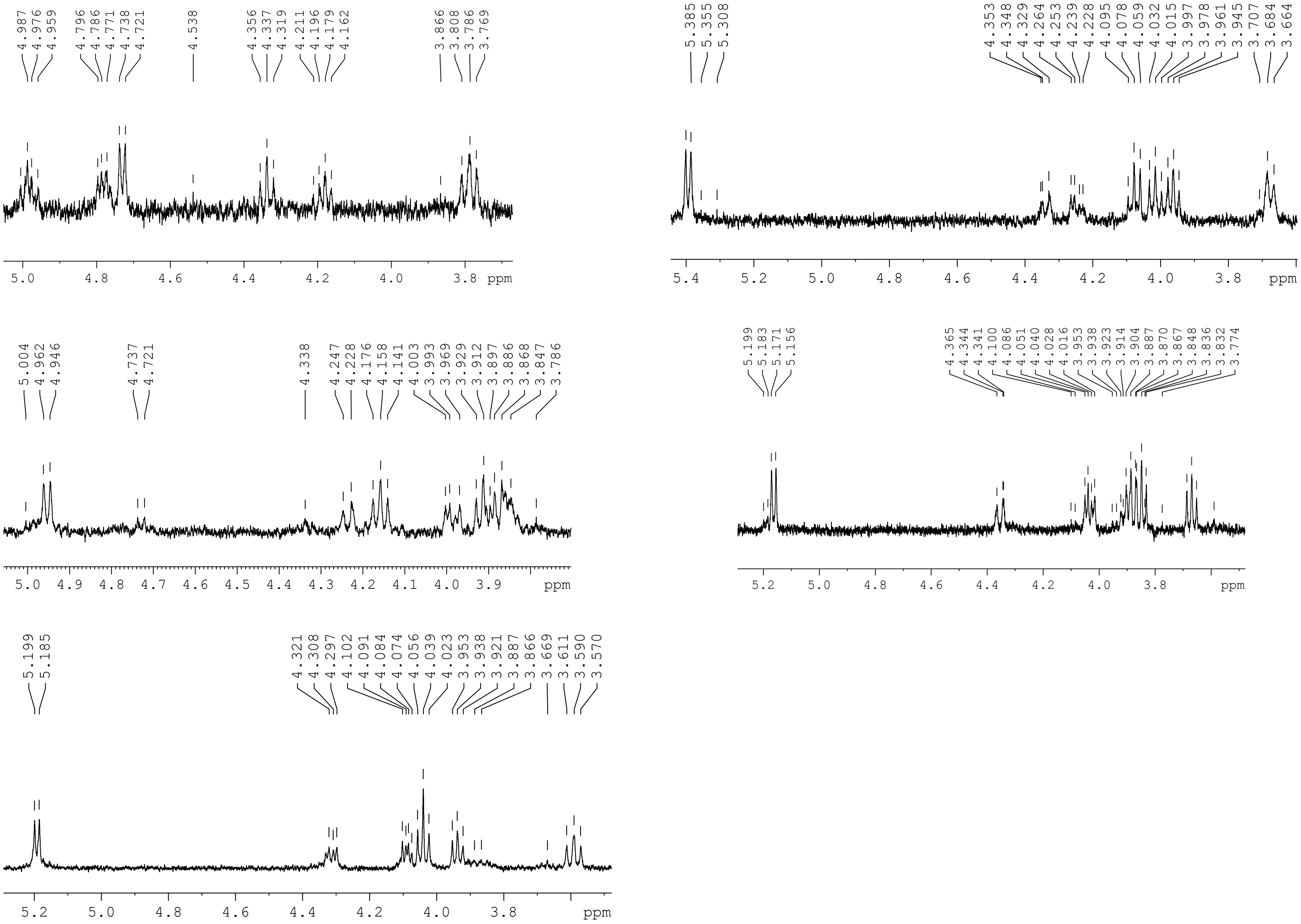

Figure S15. 1D TOCSY (500.12 MHz) spectra of Xyl1, Glc2, Glc3, MeGlc4, Xyl5 of djakonovioside D1 (**2**) in C_5D_5N/D_2O (4/1)

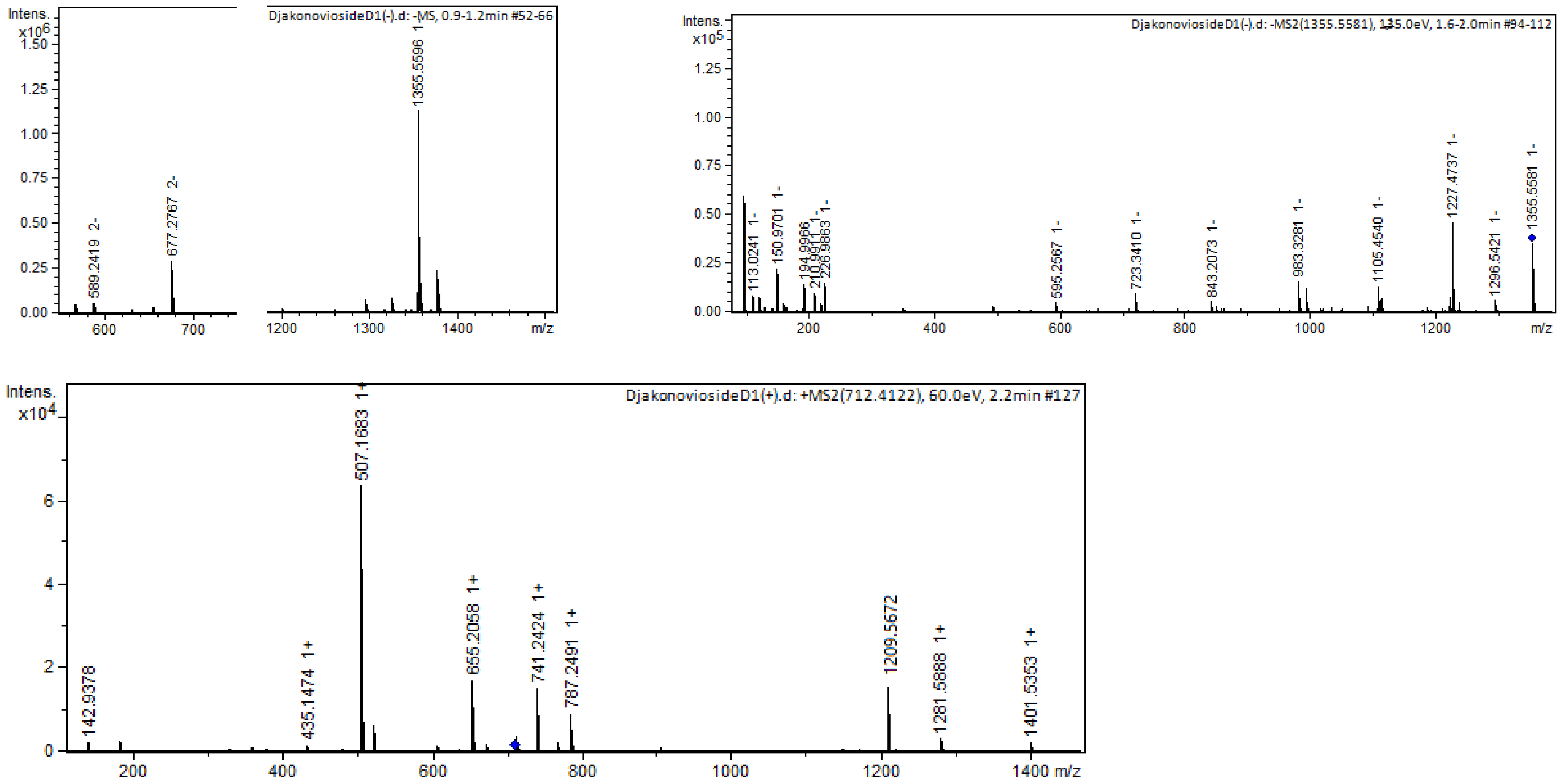

Figure S16. HR-ESI-MS and ESI-MS/MS spectra of djakonovioside D1 (**2**)

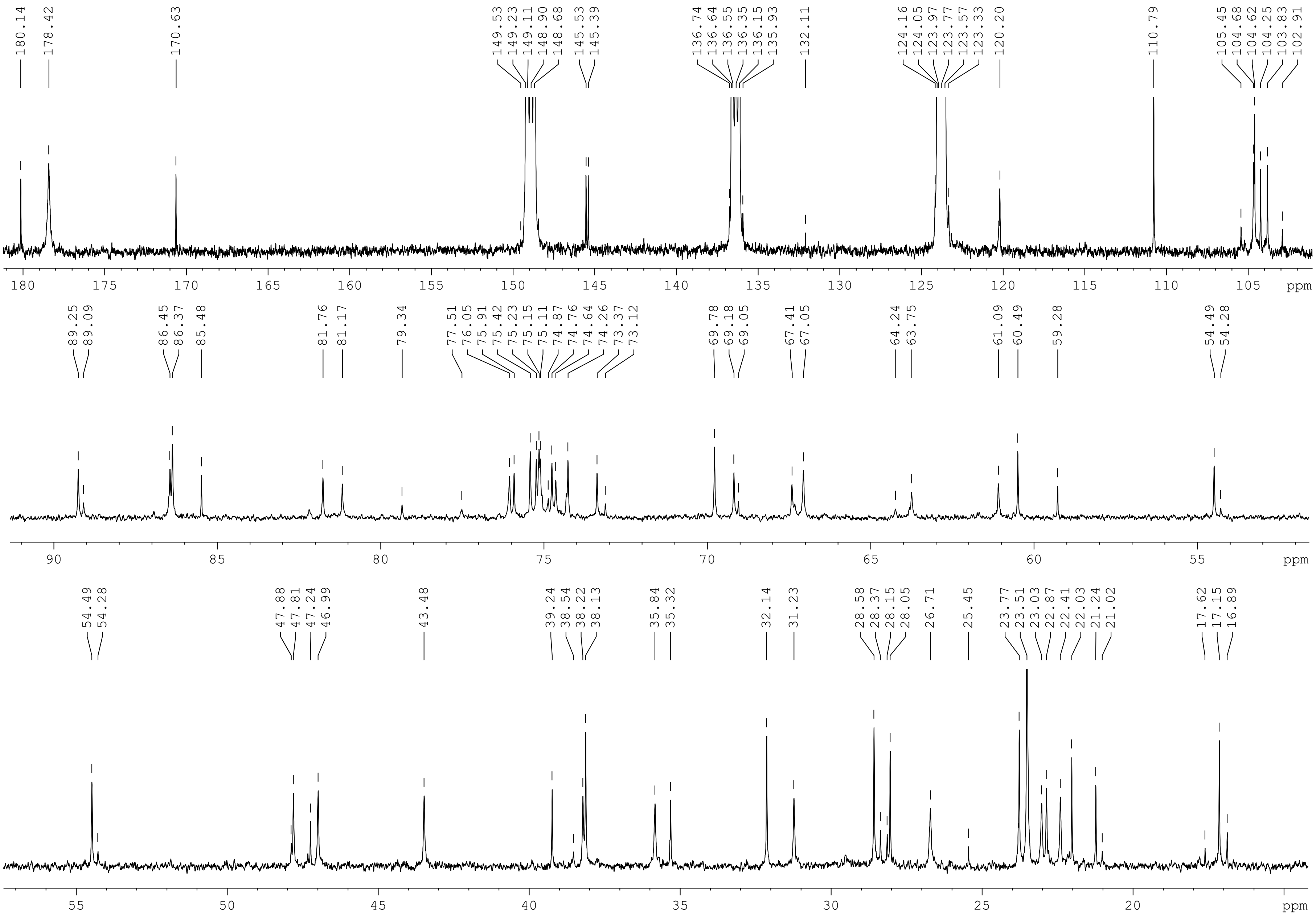

Figure S17. The ^{13}C NMR (125.67 MHz) spectrum of djakonovioside E_1 (**3**) in C_5D_5N/D_2O (4/1)

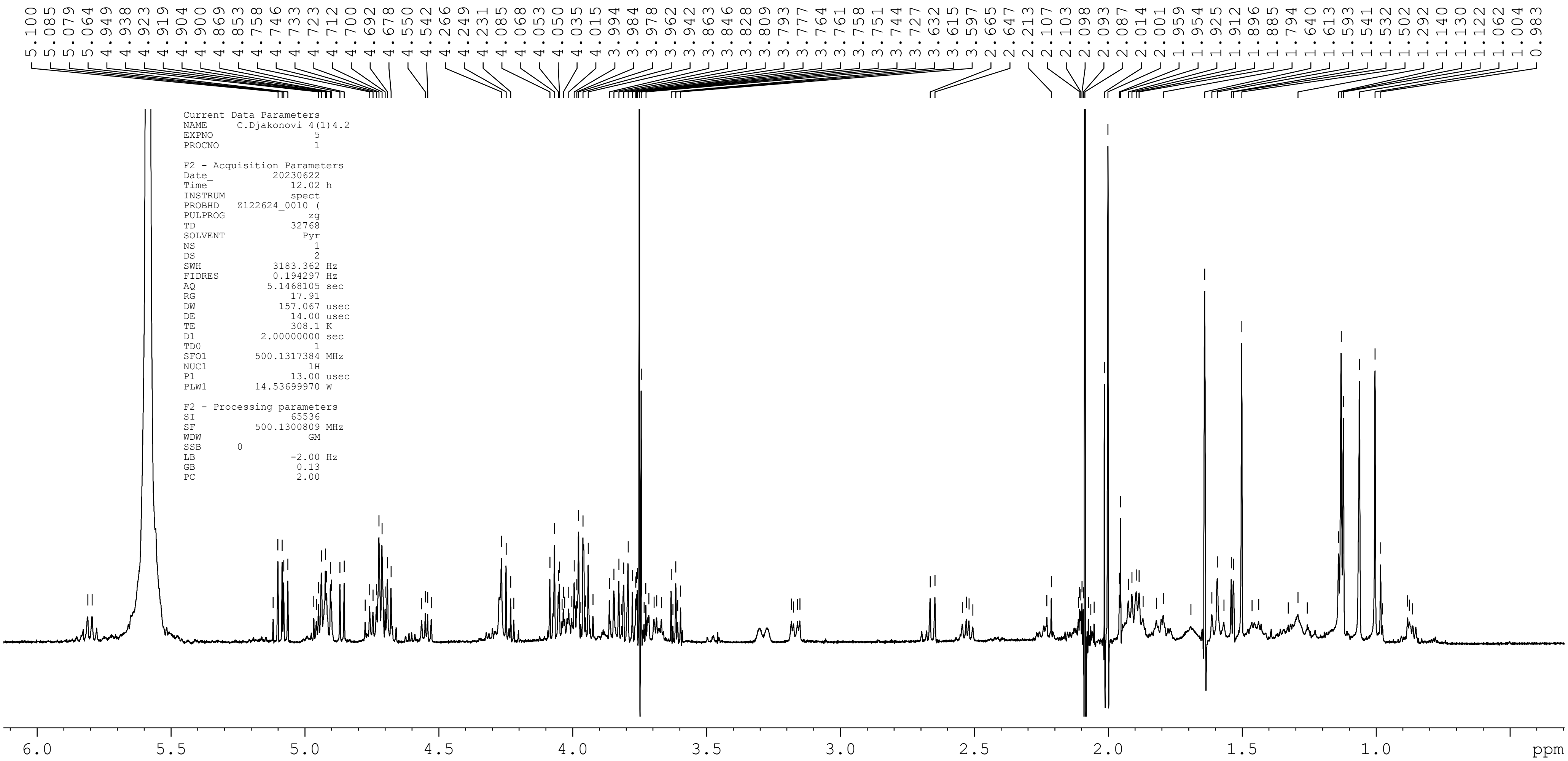

Figure S18. The ^{1}H NMR (500.12 MHz) spectrum of djakonovioside E_1 (**3**) in C_5D_5N/D_2O (4/1)

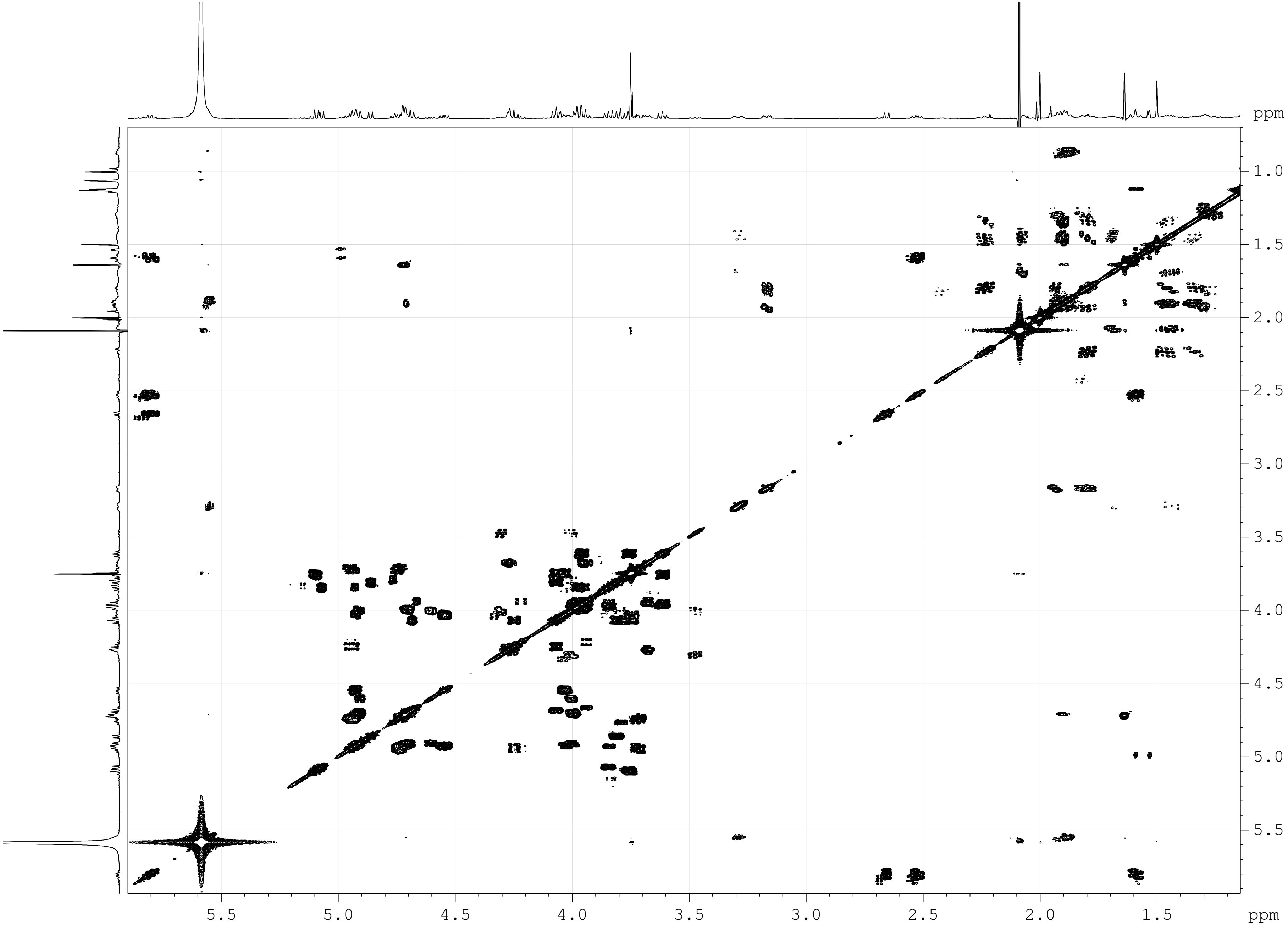

Figure S19. The COSY (500.12 MHz) spectrum of djakonovioside E_1 (**3**) in C_5D_5N/D_2O (4/1)

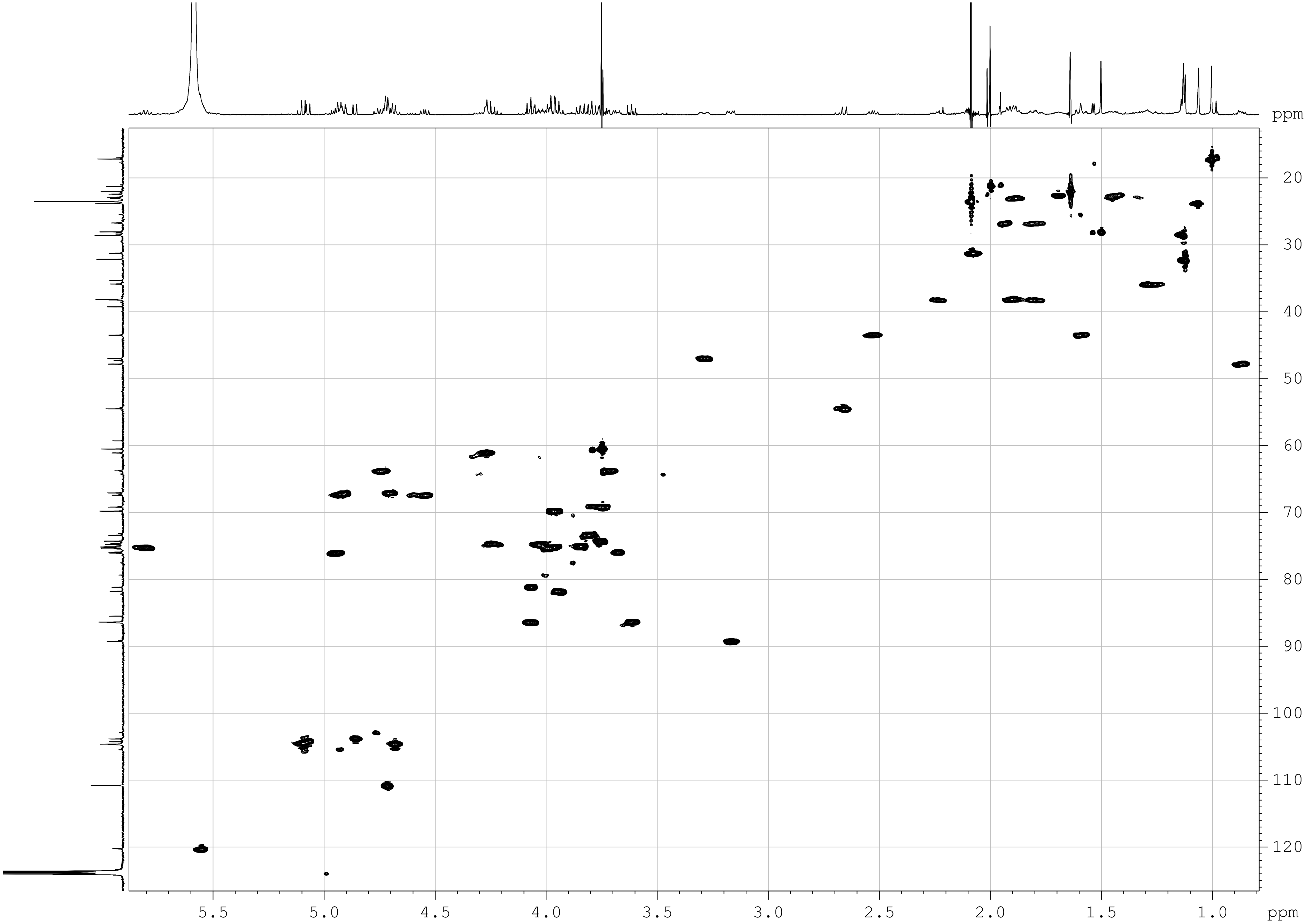

Figure S20. The HSQC (500.12 MHz) spectrum of djakonovioside E_1 (**3**) in C_5D_5N/D_2O (4/1)

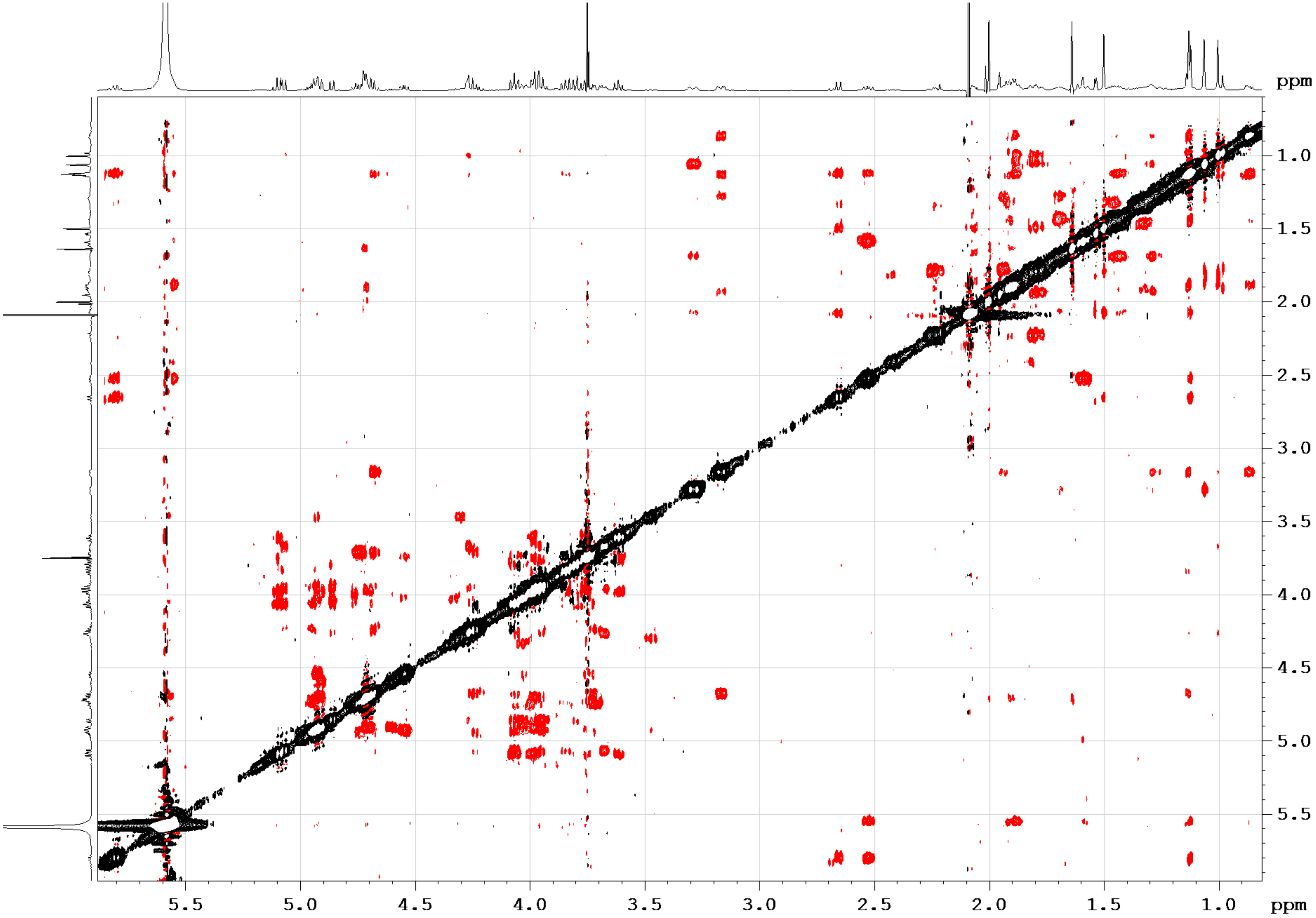

Figure S21. The ROESY (500.12 MHz) spectrum of djakonovioside E_1 (**3**) in C_5D_5N/D_2O (4/1)

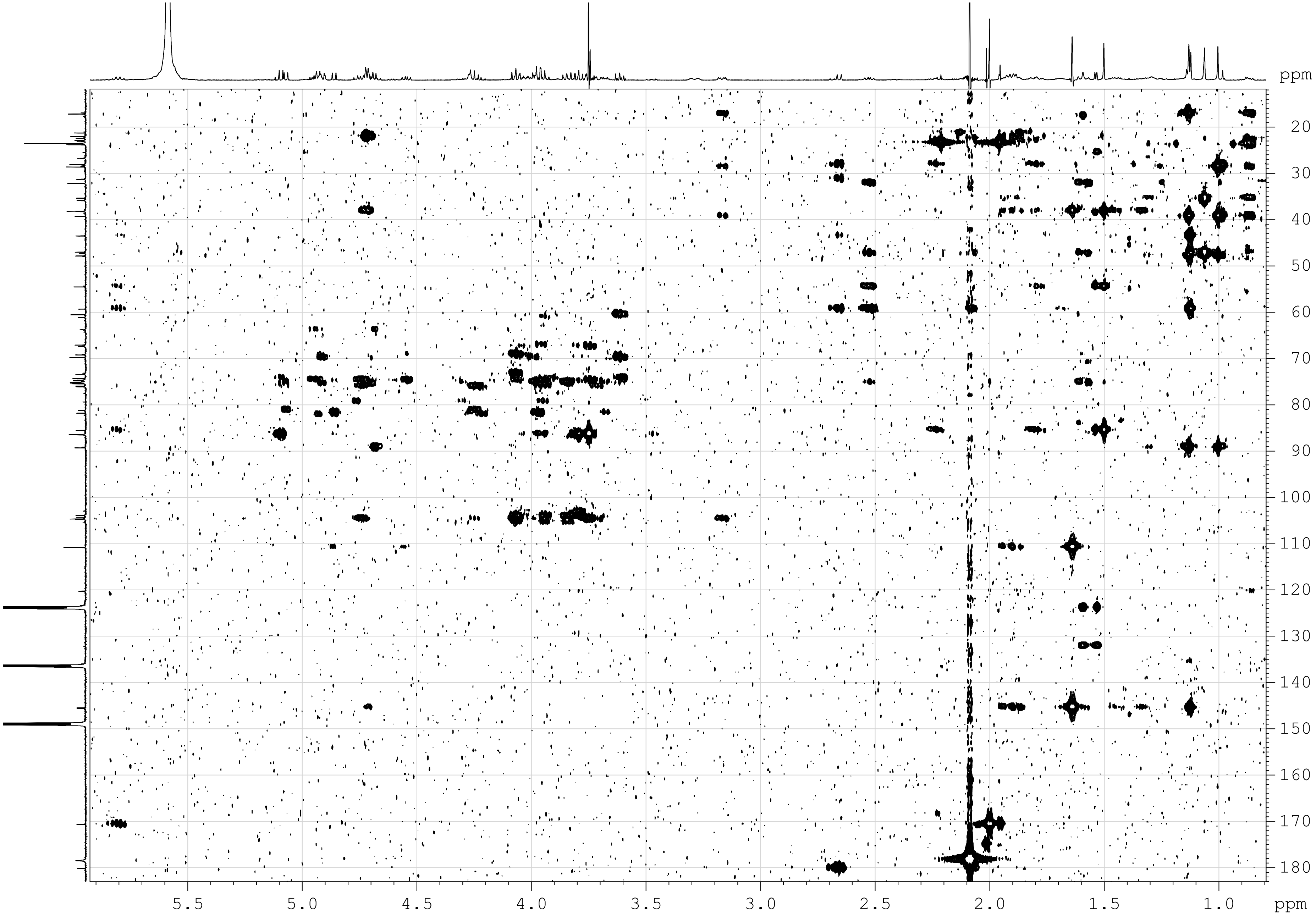

Figure S22. The HMBC (500.12 MHz) spectrum of djakonovioside E1 (**3**) in C_5D_5N/D_2O (4/1)

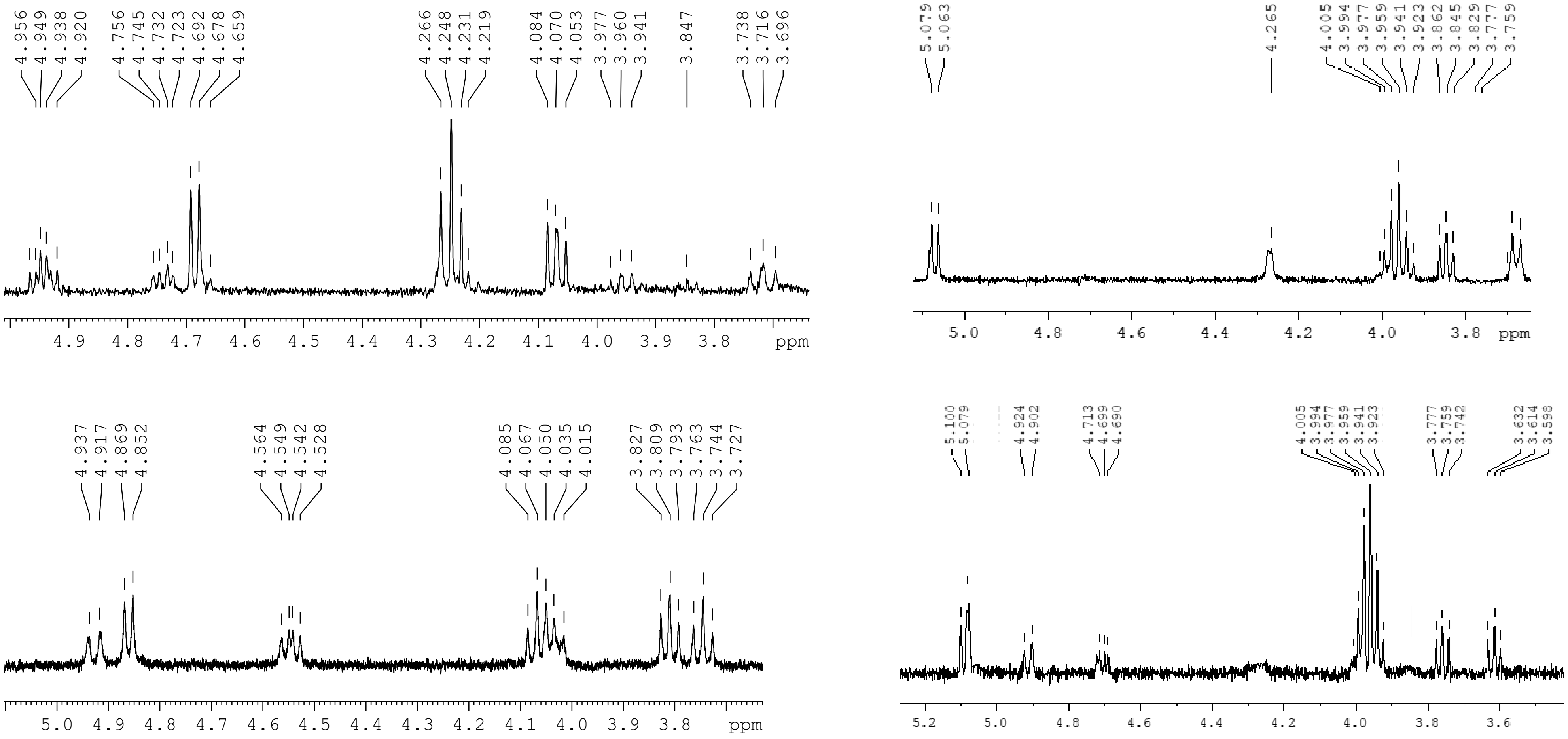

Figure S23. 1D TOCSY (500.12 MHz) spectra of Xyl1, Glc2, Glc3, MeGlc4 of djakonovioside E1 (**3**) in C_5D_5N/D_2O (4/1)

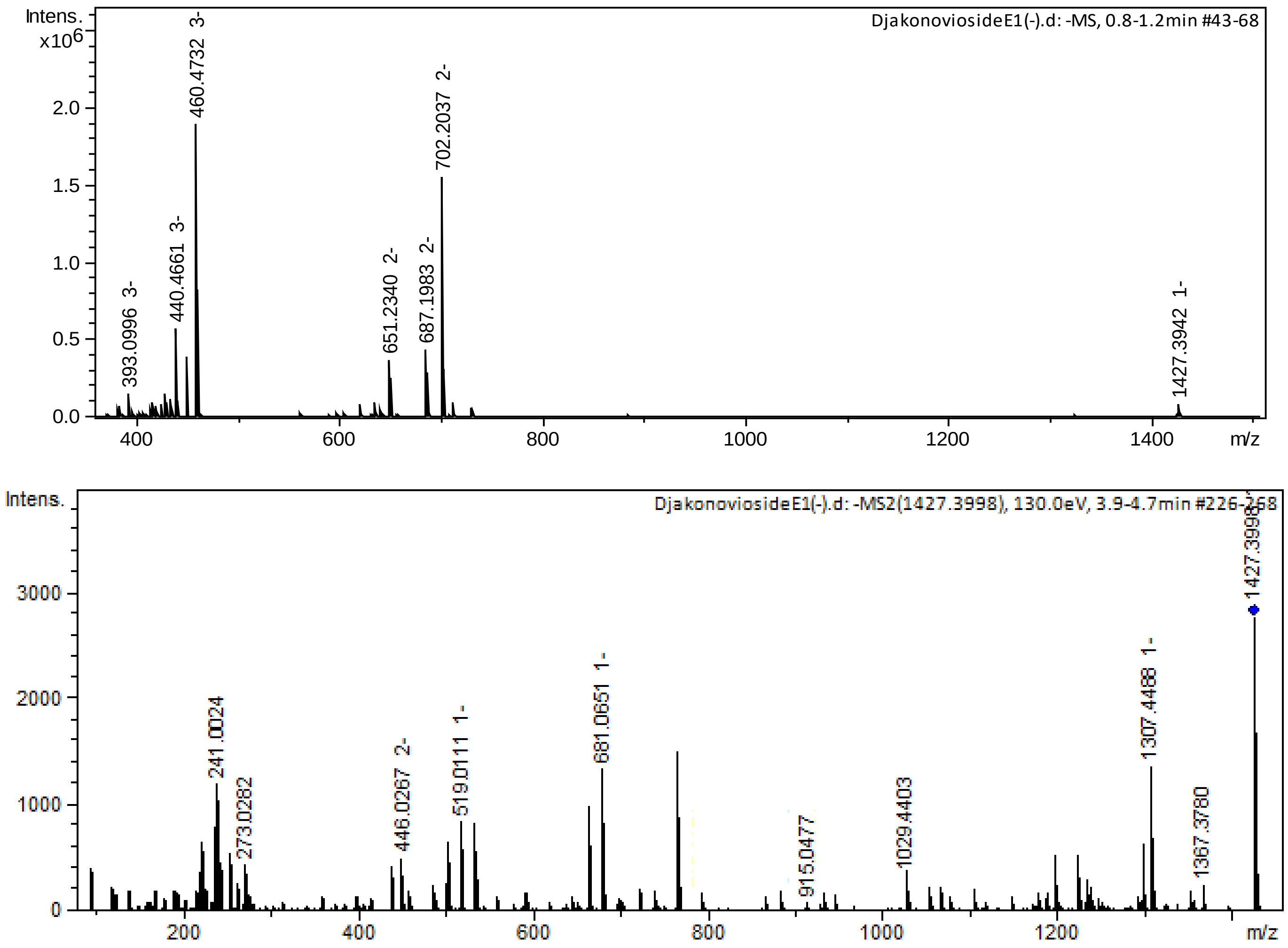

Figure S24. HR-ESI-MS and ESI-MS/MS spectra of djakonovioside E$_1$ (**3**)

Table S1. ^{13}C and ^{1}H NMR chemical shifts, HMBC and ROESY correlations of the aglycone part of djakonovioside E_1 (**3**)

Position	δ_C mult.[a]	δ_H mult. (*J* in Hz)[b]	HMBC	ROESY
1	35.8 CH_2	1.29 m		H-3, H-11, H-19
2	26.7 CH_2	1.93 m		
		1.80 m		
3	89.3 CH	3.17 dd (4.1; 11.6)		H-1, H-5, H-31, H1-Xyl1
4	39.2 C			
5	47.9 CH	0.87 dd (6.6; 12.4)	C: 19	H-1, H-3, H-31
6	23.0 CH_2	1.89 m		
7	120.4 CH	5.55 m		H-15, H-32
8	145.5 C			
9	47.0 CH	3.29 brd (14.0)		H-19
10	35.3 C			
11	22.4 CH_2	1.68 m		H-1
		1.44 m		
12	31.2 CH_2	2.07 m		H-17, H-21, H-32
13	59.3 C			
14	47.2 C			
15	43.5 CH_2	2.53 dd (7.4; 12.4)	C: 13, 14, 17, 32	H-7, H-32
		1.59 brt (12.4)		
16	75.2 CH	5.80 dd (8.5; 16.8)		H-32
17	54.5 CH	2.66 d (9.1)	C: 12, 13, 18, 21	H-12, H-16, H-21, H-32
18	180.1 C			
19	23.8 CH_3	1.06 s	C: 5, 9, 10	H-1, H-2, H-6, H-9
20	85.5 C			
21	28.0 CH_3	1.50 s	C: 17, 20, 22	H-12, H-17, H-22
22	38.2 CH	2.23 m		
		1.80 m		H-21
23	22.9 CH_2	1.47 m		
		1.35 m		
24	38.1 CH_2	1.90 m		
25	145.4 C			
26	110.8 CH_2	4.72 m	C: 24, 27	H-24
27	22.0 CH_3	1.64 s	C: 24, 25, 26	
30	17.1 CH_3	1.00 s	C: 3, 4, 5, 31	H-2, H-6, H-31
31	28.6 CH_3	1.13 s	C: 3, 4, 5, 30	H-3, H-5, H-6, H-30, H-1 Xyl1
32	32.1 CH_3	1.12 s	C: 8, 13, 14, 15	H-7, H-12, H-15, H-16, H-17
O<u>C</u>OCH$_3$	170.6 C			
OCO<u>C</u>H$_3$	21.0 CH_3	2.00 s	OAc	

[a] Recorded at 125.67 MHz in C_5D_5N/D_2O (4/1). [b] Recorded at 500.12 MHz in C_5D_5N/D_2O (4/1).

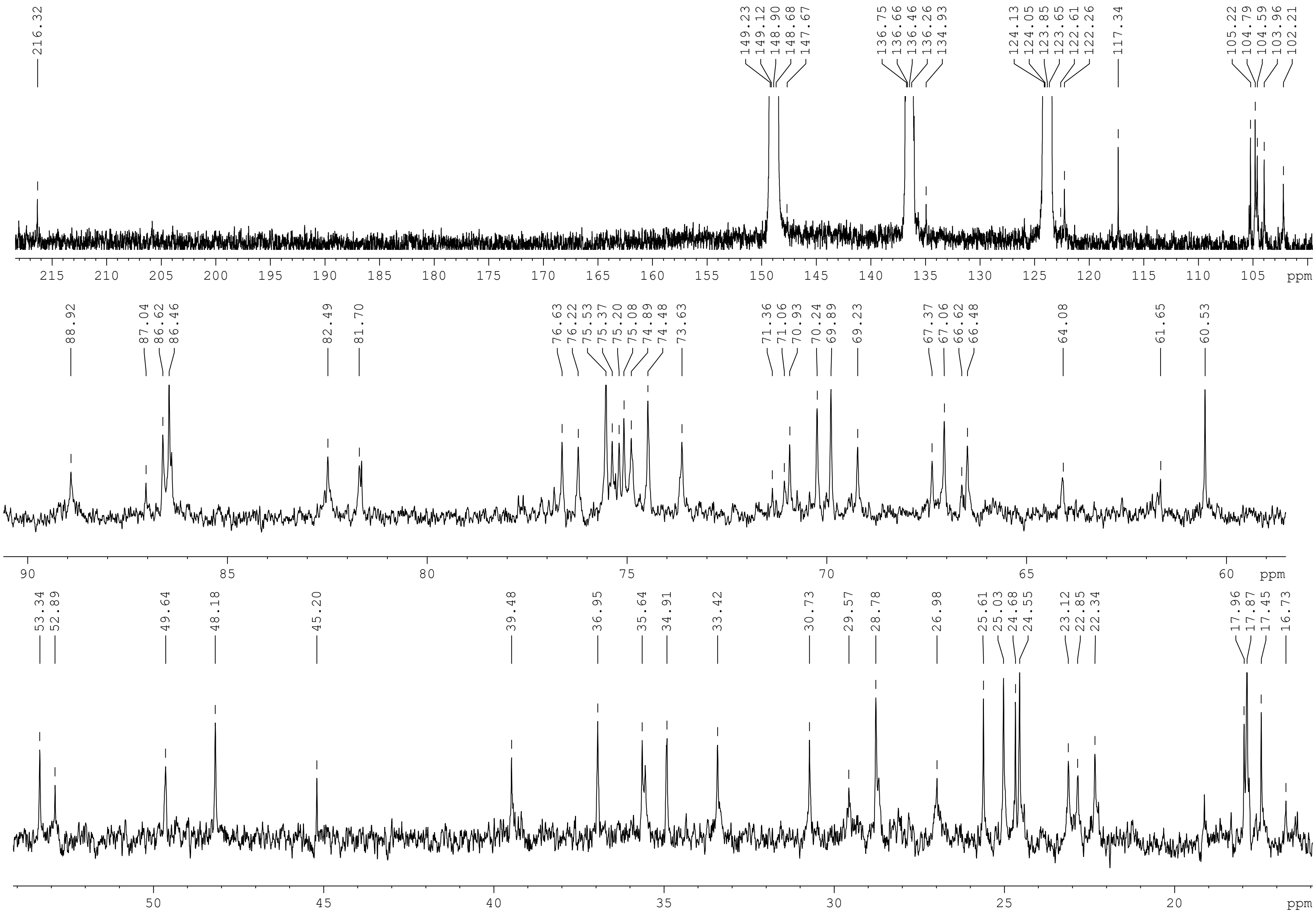

Figure S25. The ^{13}C NMR (125.67 MHz) spectrum of djakonovioside F_1 (**4**) in C_5D_5N/D_2O (4/1)

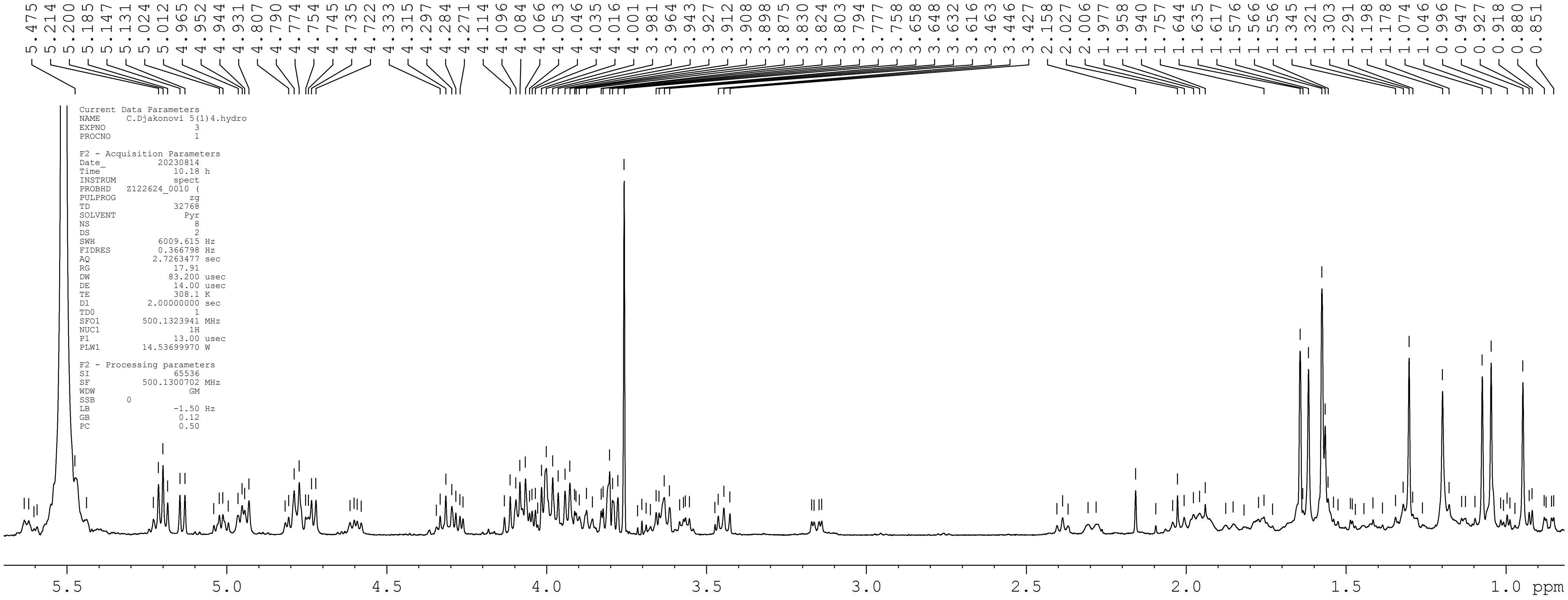

Figure S26. The ^{1}H NMR (500.12 MHz) spectrum of djakonovioside F$_1$ (**4**) in C_5D_5N/D_2O (4/1)

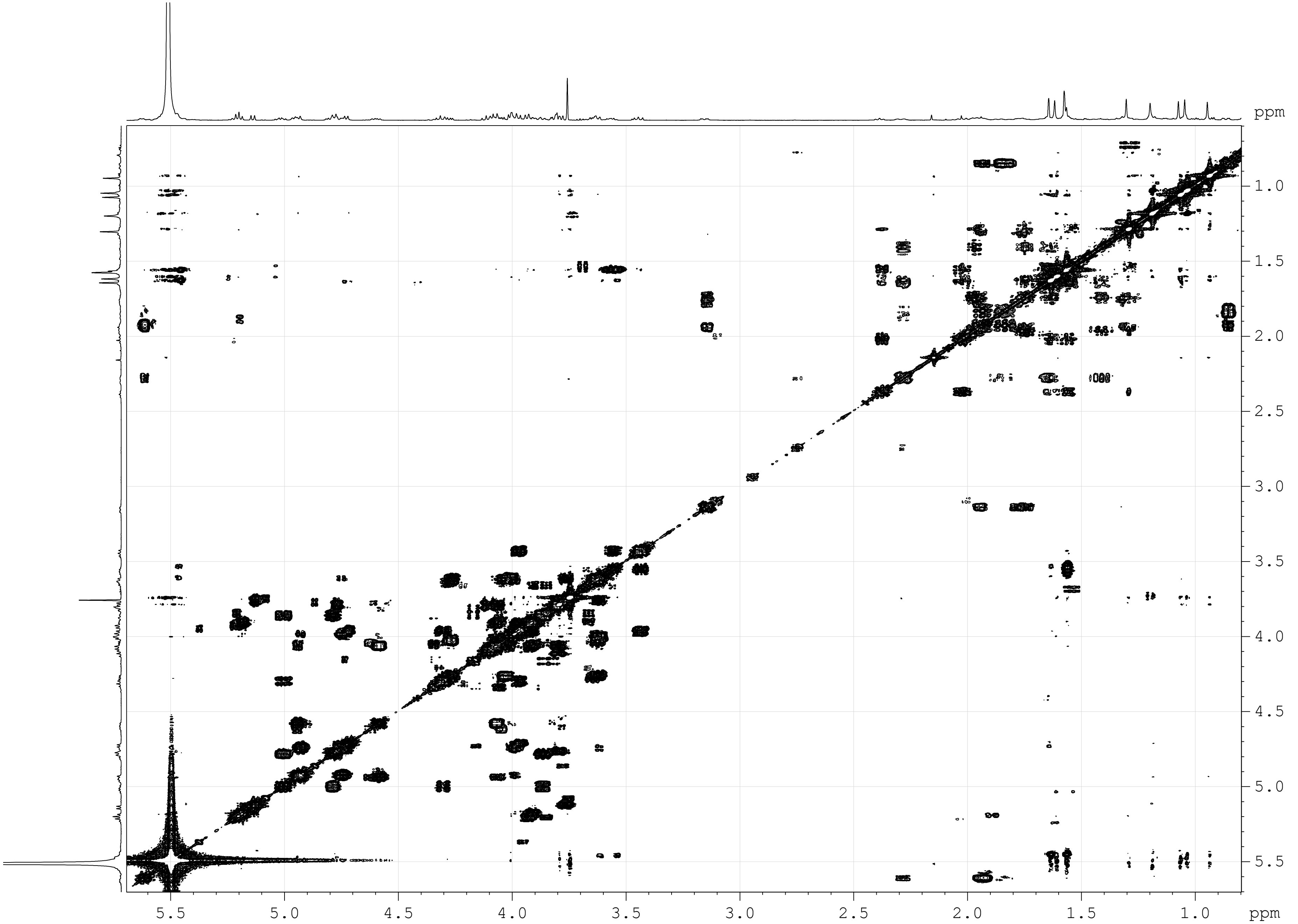

Figure S27. The COSY (500.12 MHz) spectrum of djakonovioside F_1 (**4**) in C_5D_5N/D_2O (4/1)

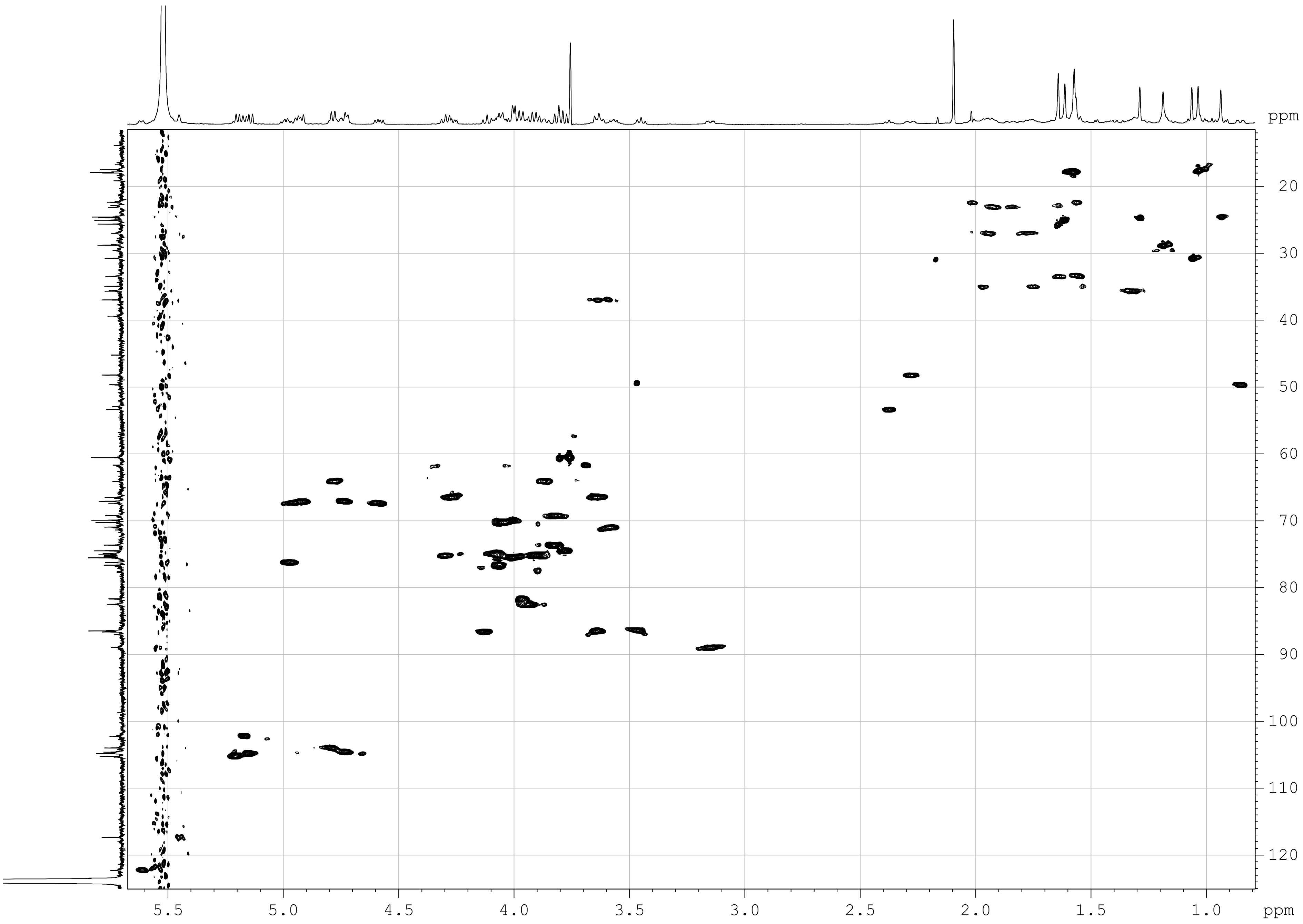

Figure S28. The HSQC (500.12 MHz) spectrum of djakonovioside F_1 (**4**) in C_5D_5N/D_2O (4/1)

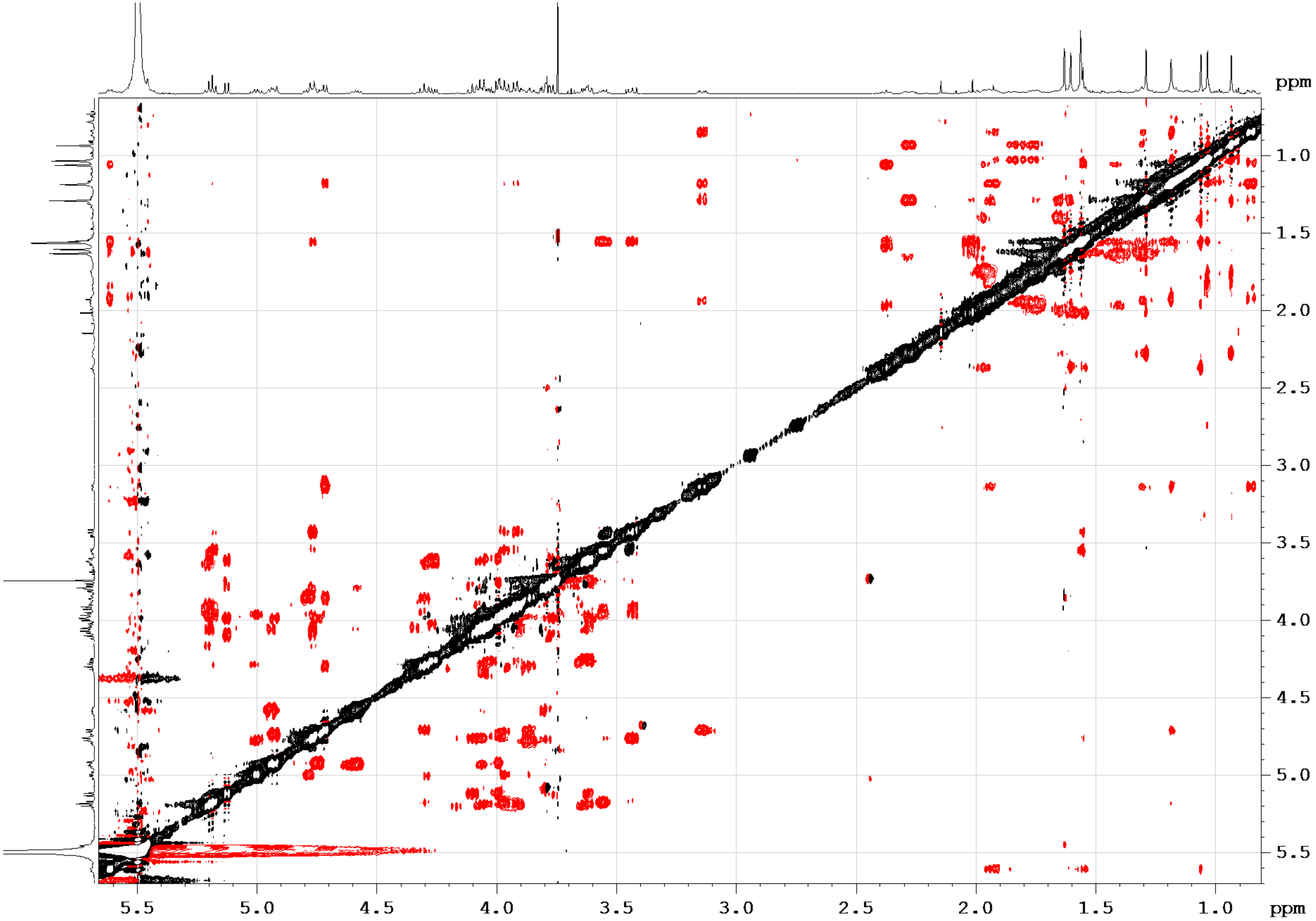

Figure S29. The ROESY (500.12 MHz) spectrum of djakonovioside F_1 (**4**) in C_5D_5N/D_2O (4/1)

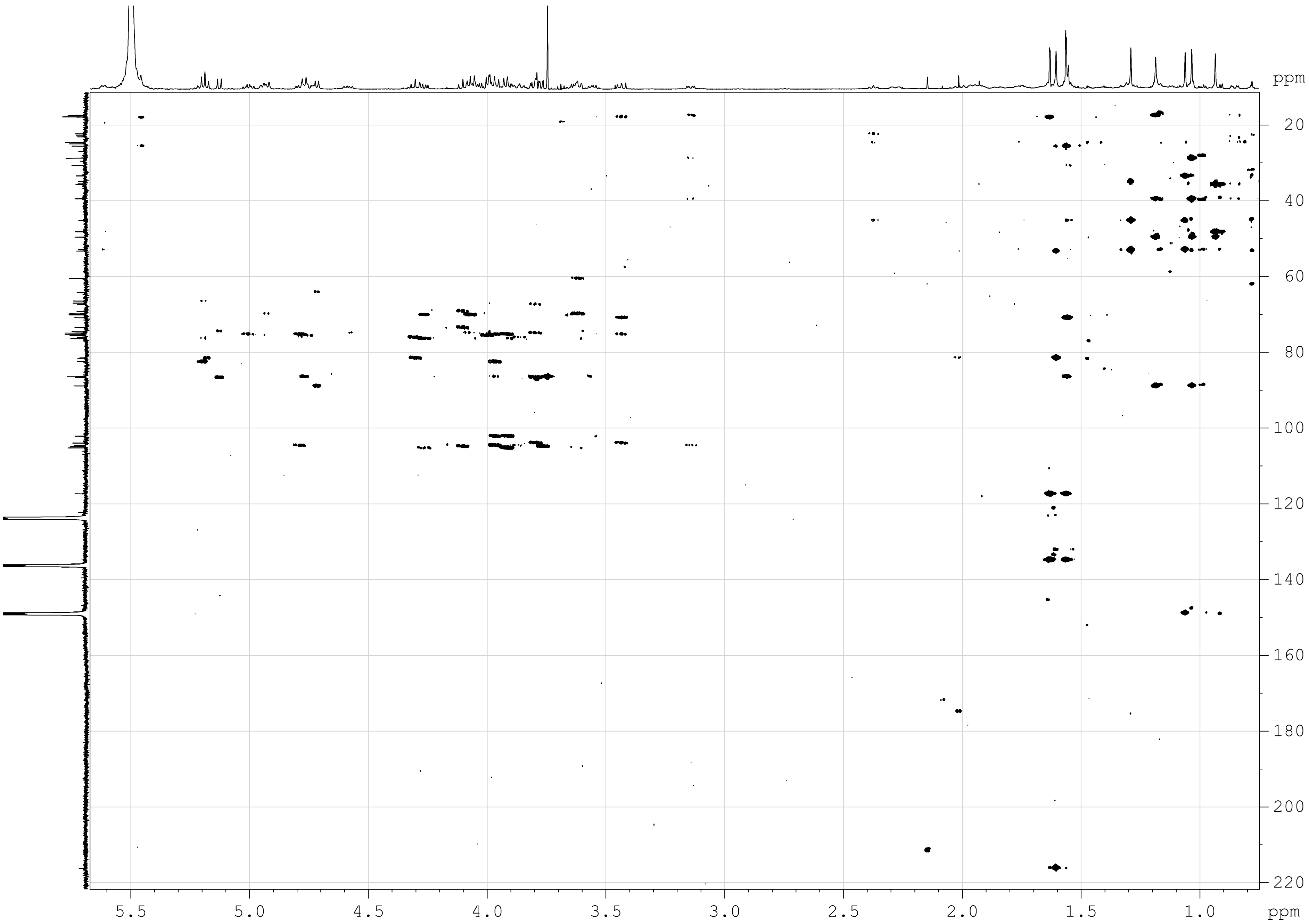

Figure S30. The HMBC (500.12 MHz) spectrum of djakonovioside F_1 (**4**) in C_5D_5N/D_2O (4/1)

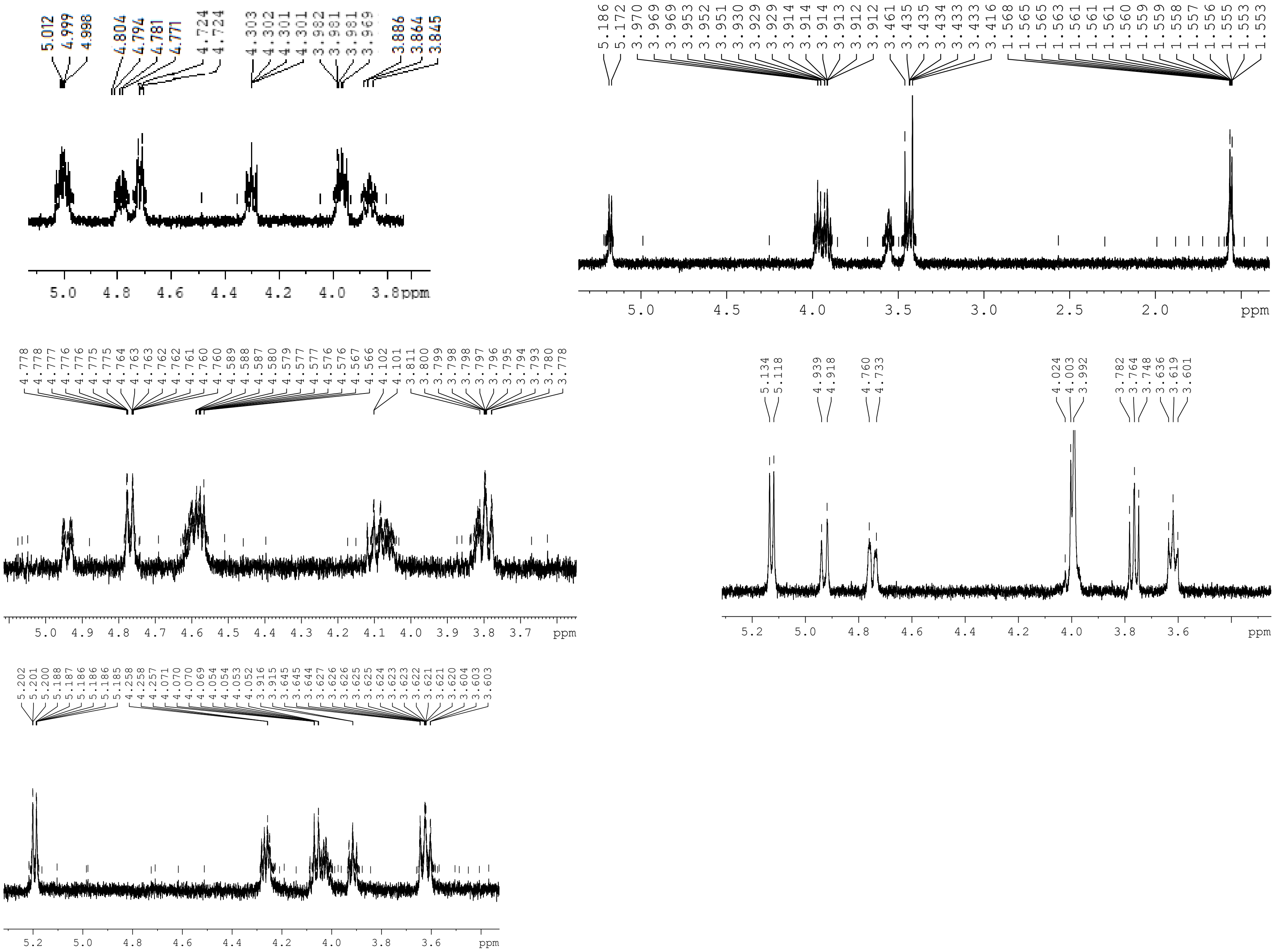

Figure S31. 1D TOCSY (500.12 MHz) spectra of Xyl1, Qui2, Glc3, MeGlc4, Xyl5 of djakonovioside F$_1$ (**4**) in C_5D_5N/D_2O (4/1)

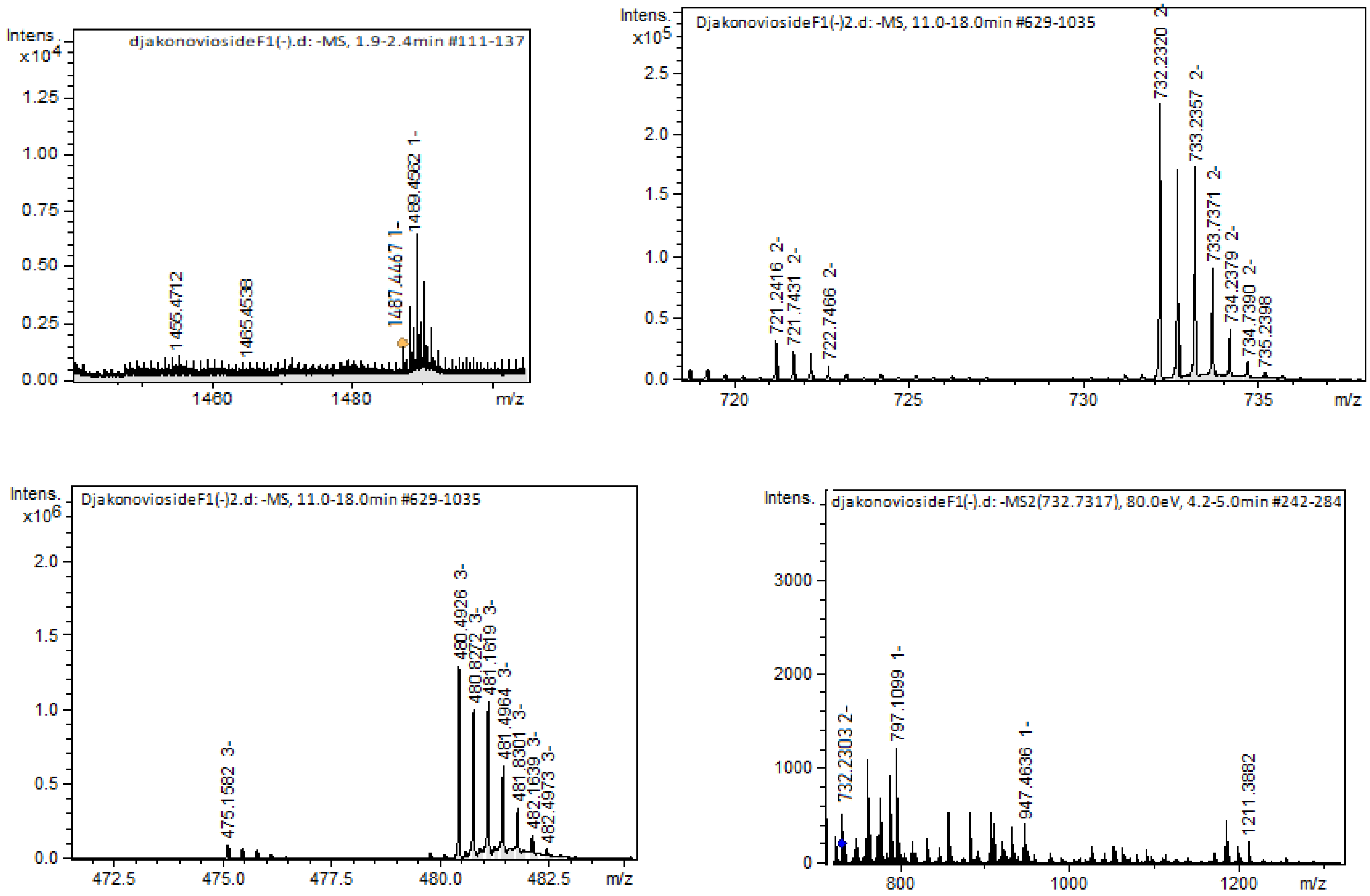

Figure S32. HR-ESI-MS and ESI-MS/MS spectra of djakonovioside F_1 (**4**)

Table S2. ^{13}C and ^{1}H NMR chemical shifts and HMBC and ROESY correlations of aglycone moiety of okhotoside A_2-1 (**5**).

Position	δ_C mult.[a]	δ_H mult. (*J* in Hz) [b]	HMBC	ROESY
1	35.8 CH_2	1.29 m		H-3, H-11, H-19
2	26.6 CH_2	1.91 m		
		1.81 m		H-19, H-30
3	89.2 CH	3.20 dd (4.0; 11.6)		H-1, H-5, H-31, H1-Xyl1
4	39.3 C			
5	47.8 CH	0.89 dd (5.3; 9.9)	C: 19	H-1, H-3, H-31
6	23.1 CH_2	1.89 m		H-19, H-30, H-31
7	120.2 CH	5.57 m		H-15, H-32
8	145.4 C			
9	47.0 CH	3.30 brd (14.5)		H-19
10	35.3 C			
11	22.4 CH_2	1.70 m		H-1
		1.47 m		
12	31.2 CH_2	2.09 m		H-17, H-21, H-32
13	59.3 C			
14	47.2 C			
15	43.5 CH_2	2.55 dd (7.6; 12.2)	C: 13, 17, 32	H-7, H-32
		1.60 dd (9.2; 12.2)		
16	75.2 CH	5.82 q (8.4)		H-32
17	54.5 CH	2.66 d (9.2)	C: 12, 13, 18, 21	H-12, H-16, H-21, H-32
18	180.1 C			
19	23.7 CH_3	1.05 s	C: 5, 9, 10	H-1, H-2, H-6, H-9
20	85.5 C			
21	28.0 CH_3	1.51 s	C: 17, 20, 22	H-12, H-17, H-22
22	38.2 CH	2.24 td (4.6; 13.0)		
		1.80 m		
23	22.9 CH_2	1.47 m		
		1.34 m		
24	38.1 CH_2	1.91 m		
25	145.5 C			
26	110.8 CH_2	4.72 m	C: 24, 27	H-24
27	22.0 CH_3	1.64 s	C: 24, 25, 26	
30	17.2 CH_3	1.04 s	C: 3, 4, 5, 31	H-2, H-6, H-31
31	28.5 CH_3	1.17 s	C: 3, 4, 5, 30	H-3, H-5, H-6, H-30
32	32.1 CH_3	1.15 s	C: 8, 13, 14, 15	H-7, H-12, H-15, H-16, H-17
O<u>C</u>OCH$_3$	170.6 C			
OCO<u>C</u>H$_3$	21.2 CH_3	2.01 s	OAc	OAc

[a] Recorded at 125.67 MHz in C_5D_5N/D_2O (4/1). [b] Recorded at 500.12 MHz in C_5D_5N/D_2O (4/1). The original spectra of **5** are provided as Figures S33–S38.

Table S3. ^{13}C and ^{1}H NMR chemical shifts and HMBC and ROESY correlations of carbohydrate moiety of okhotoside A$_2$-1 (**5**).

Atom	δC mult.[a]	δH mult. (*J* in Hz)[b,c,d]	HMBC	ROESY
Xyl1 (1→C-3)				
1	104.6 CH	4.72 d (7.4)	C: 3	H-3; H-3, 5 Xyl1
2	**79.7** CH	4.17 t (8.9)	C: 1 Glc2; C: 1 Xyl1	H-1 Glc2
3	75.5 CH	4.33 t (8.9)	C: 4 Xyl1	H-1 Xyl1
4	*76.0* CH	4.98 m		
5	64.2 CH_2	4.77 dd (5.2; 11.9)	C: 4 Xyl1	
		3.77 brd (11.1)		
Glc2 (1→2Xyl1)				
1	101.5 CH	5.39 d (7.5)	C: 2 Xyl1	H-2 Xyl1; H-5 Glc2
2	**82.2** CH	3.96 t (8.4)	C: 1 Xyl5, C: 1, 3 Glc2	H-1 Xyl5
3	75.6 CH	4.08 t (8.4)	C: 2, 4 Glc2	H-1 Glc2
4	**80.6** CH	4.02 t (9.2)	C: 1 Glc3; C: 5 Glc2	H-1 Glc3
5	75.8 CH	3.67 brd (9.2)		H-1 Glc2
6	61.4 CH_2	4.34 dd (11.7; 2.5)		
		4.25 dd (11.7; 5.0)		
Glc3 (1→4Glc2)				
1	103.6 CH	4.96 d (8.4)	C: 4 Glc2	H-4 Glc2; H-3, 5 Glc3
2	73.5 CH	3.90 t (8.4)	C: 1, 3 Glc3	
3	**86.8** CH	4.15 t (8.4)	C: 2, 4 Glc3	H-1 MeGlc4
4	69.2 CH	3.88 t (8.4)		
5	77.1 CH	3.85 t (8.4)		H-1 Glc3
6	61.1 CH_2	4.25 brd (11.9)		
		3.98 m		
MeGlc4 (1→3Glc3)				
1	104.5 CH	5.16 d (8.3)	C: 3 Glc3	H-3 Glc3; H-3, 5 MeGlc4
2	74.5 CH	3.85 t (9.0)	C: 1, 3 MeGlc4	
3	86.7 CH	3.66 t (9.0)	OMe	H-1 MeGlc4; OMe
4	70.3 CH	3.87 m	C: 5 MeGlc4	
5	77.5 CH	3.89 m		H-1 MeGlc4
6	61.7 CH_2	4.36 dd (11.8; 2.9)	C: 4 MeGlc4	
		4.04 dd (11.8; 5.5)	C: 4, 5 MeGlc4	
OMe	60.7 CH_3	3.80 s	C: 3 MeGlc4	
Xyl5 (1→2Glc2)				
1	105.2 CH	5.20 d (6.9)	C: 2 Glc2	H-2 Glc2; H-3, 5 Xyl5
2	74.7 CH	3.94 t (9.0)	C: 1, 3 Xyl5	
3	76.3 CH	4.04 t (9.0)	C: 2 Xyl5	H-1 Xyl5
4	70.1 CH	4.10 m		
5	66.3 CH_2	4.31 dd (11.8; 4.8)		
		3.59 dd (11.8; 9.7)		H-1 Xyl5

[a] Recorded at 125.67 MHz in C_5D_5N/D_2O. [b] Bold = interglycosidic positions. [c] Italic = sulfate position. [d] Recorded at 500.12 MHz in C_5D_5N/D_2O. The original spectra of **5** are provided as Figures S33–S38.

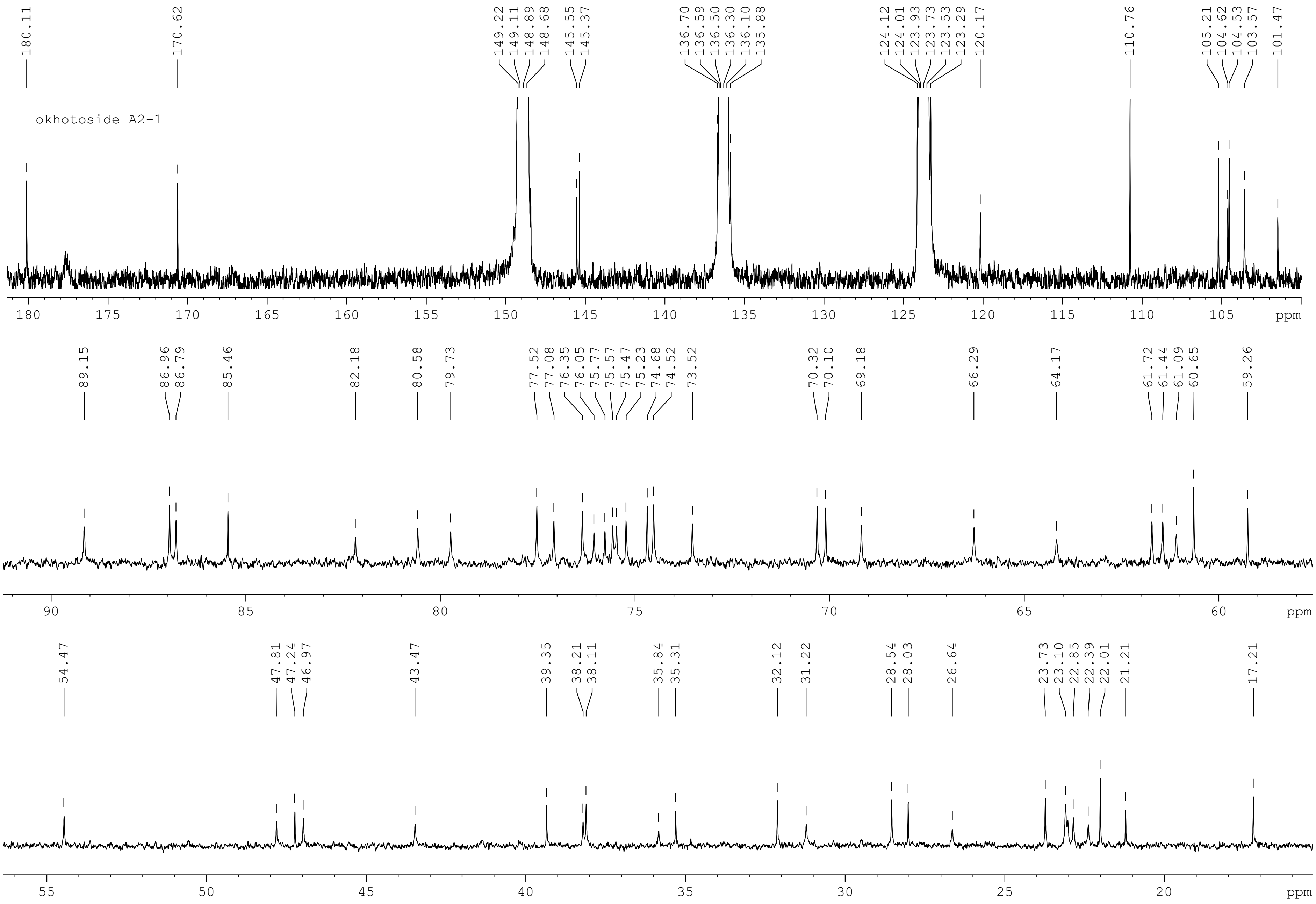

Figure S33. The ^{13}C NMR (125.67 MHz) spectrum of okhotoside A_2-1 (**5**) in C_5D_5N/D_2O (4/1)

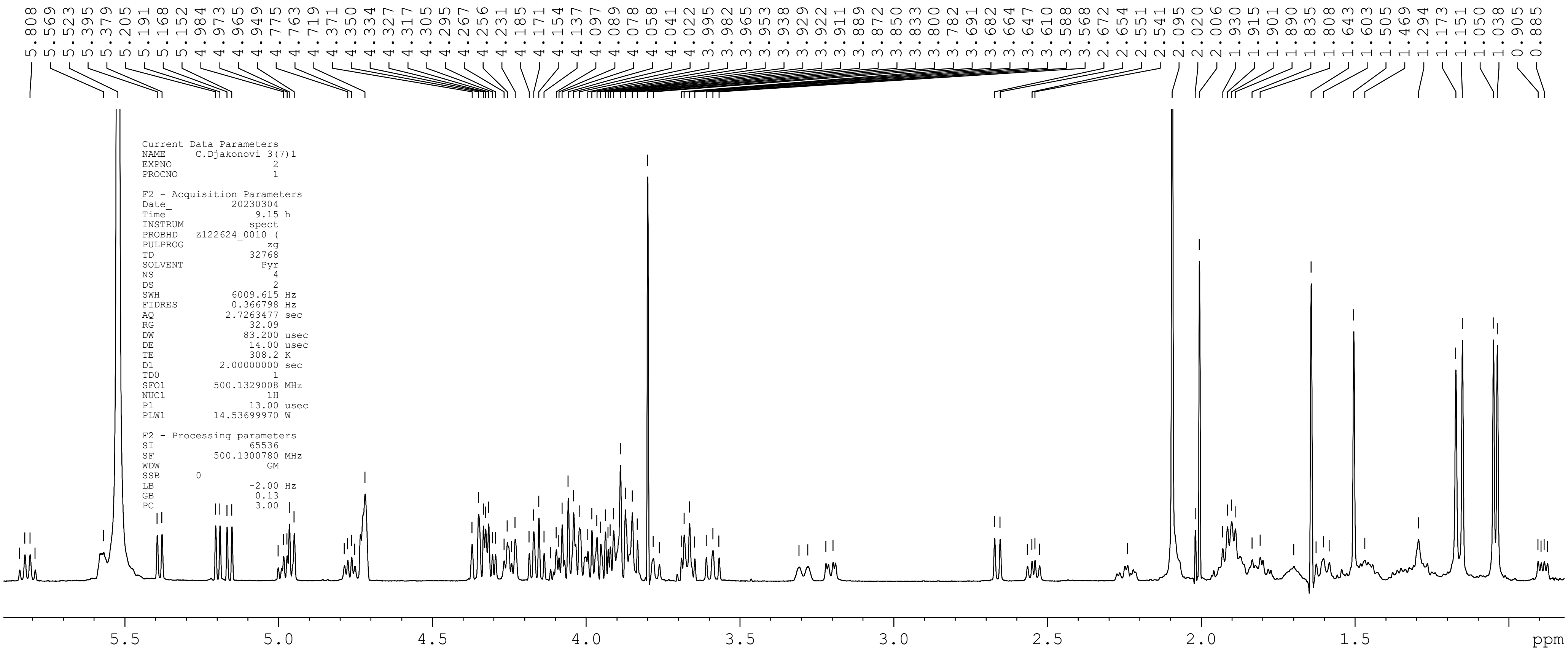

Figure S34. The ^{1}H NMR (500.12 MHz) spectrum of okhotoside A_2-1 (**5**) in C_5D_5N/D_2O (4/1)

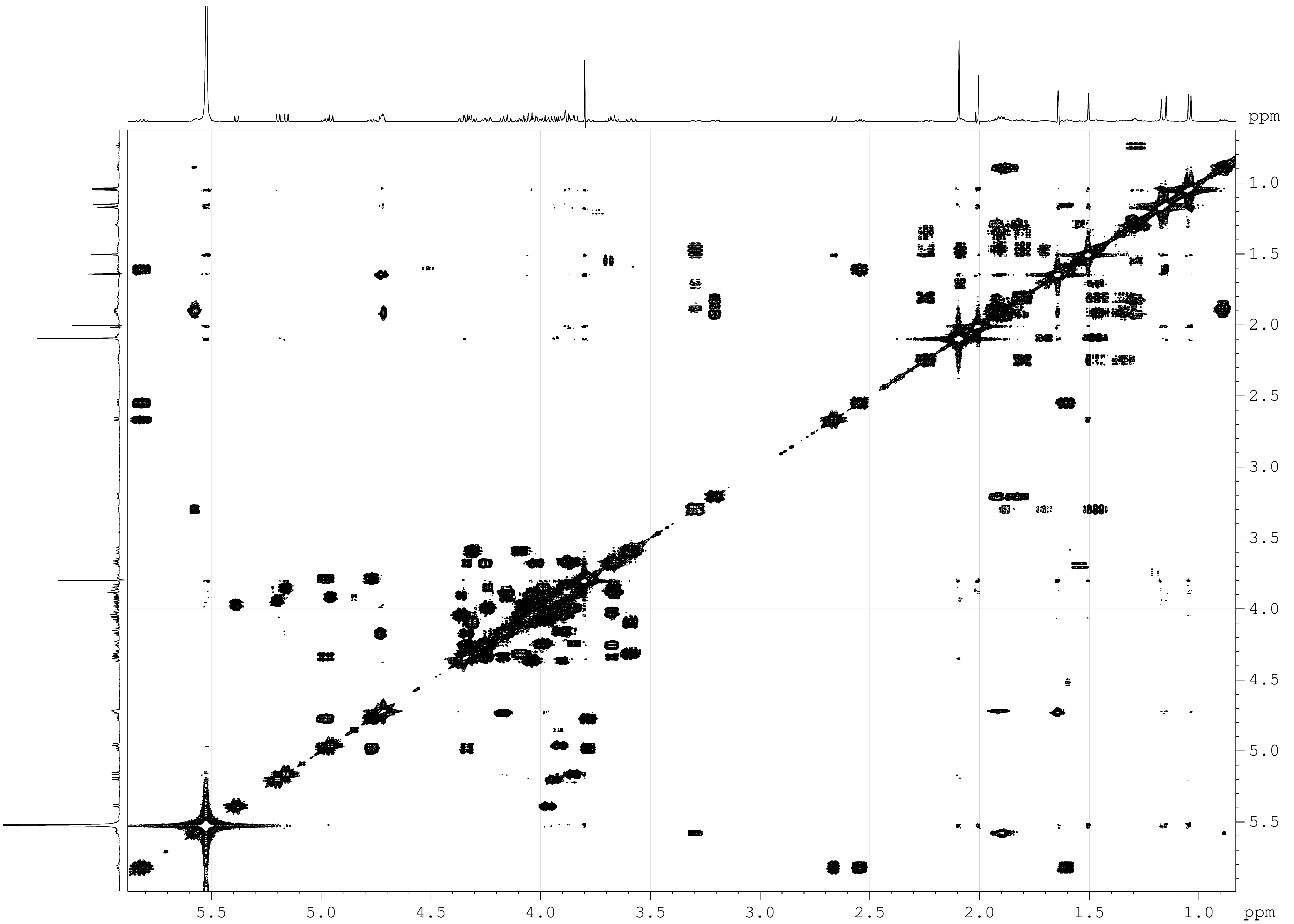

Figure S35. The COSY (500.12 MHz) spectrum of okhotoside A_2-1 (**5**) in C_5D_5N/D_2O (4/1)

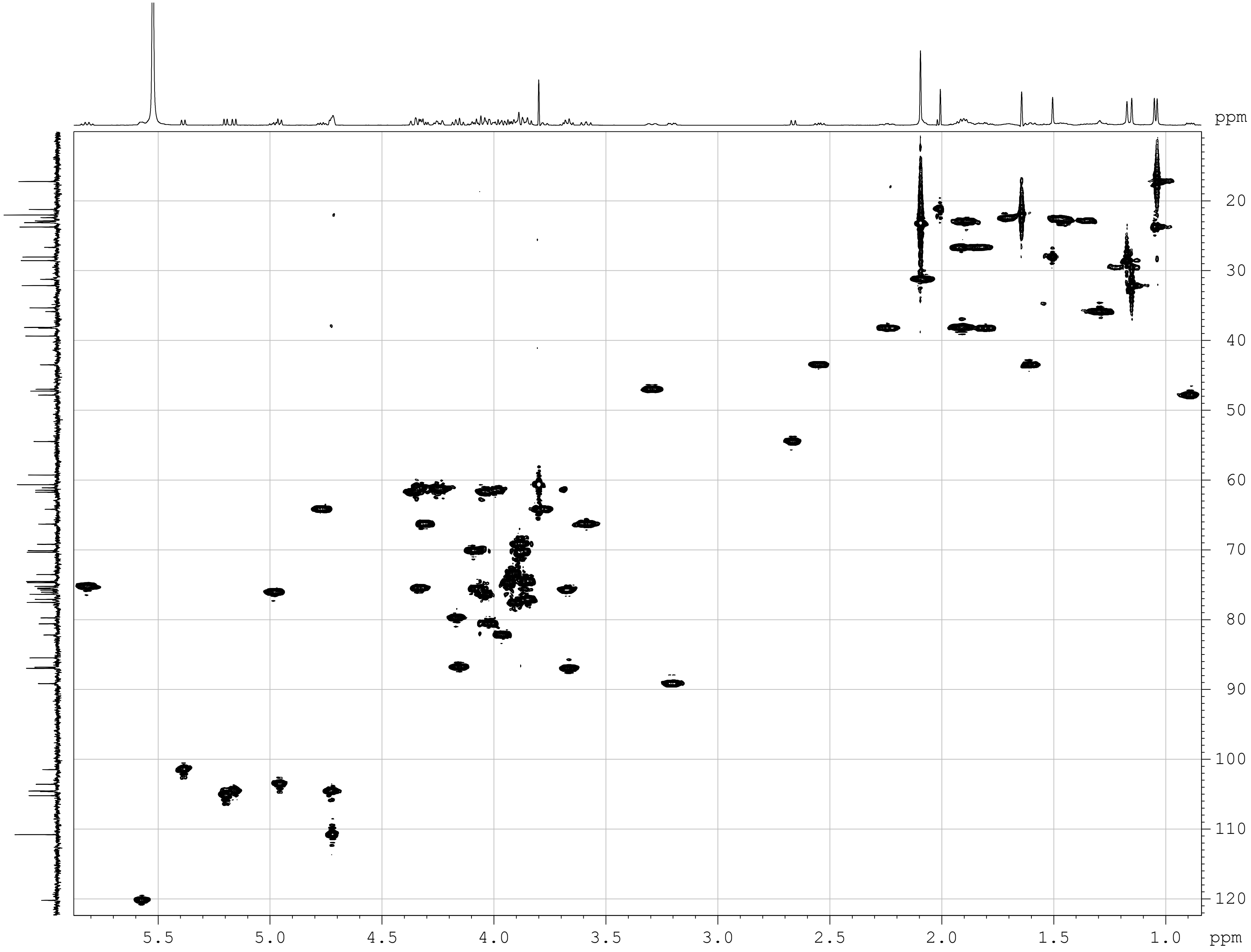

Figure S36. The HSQC (500.12 MHz) spectrum of okhotoside A_2-1 (**5**) in C_5D_5N/D_2O (4/1)

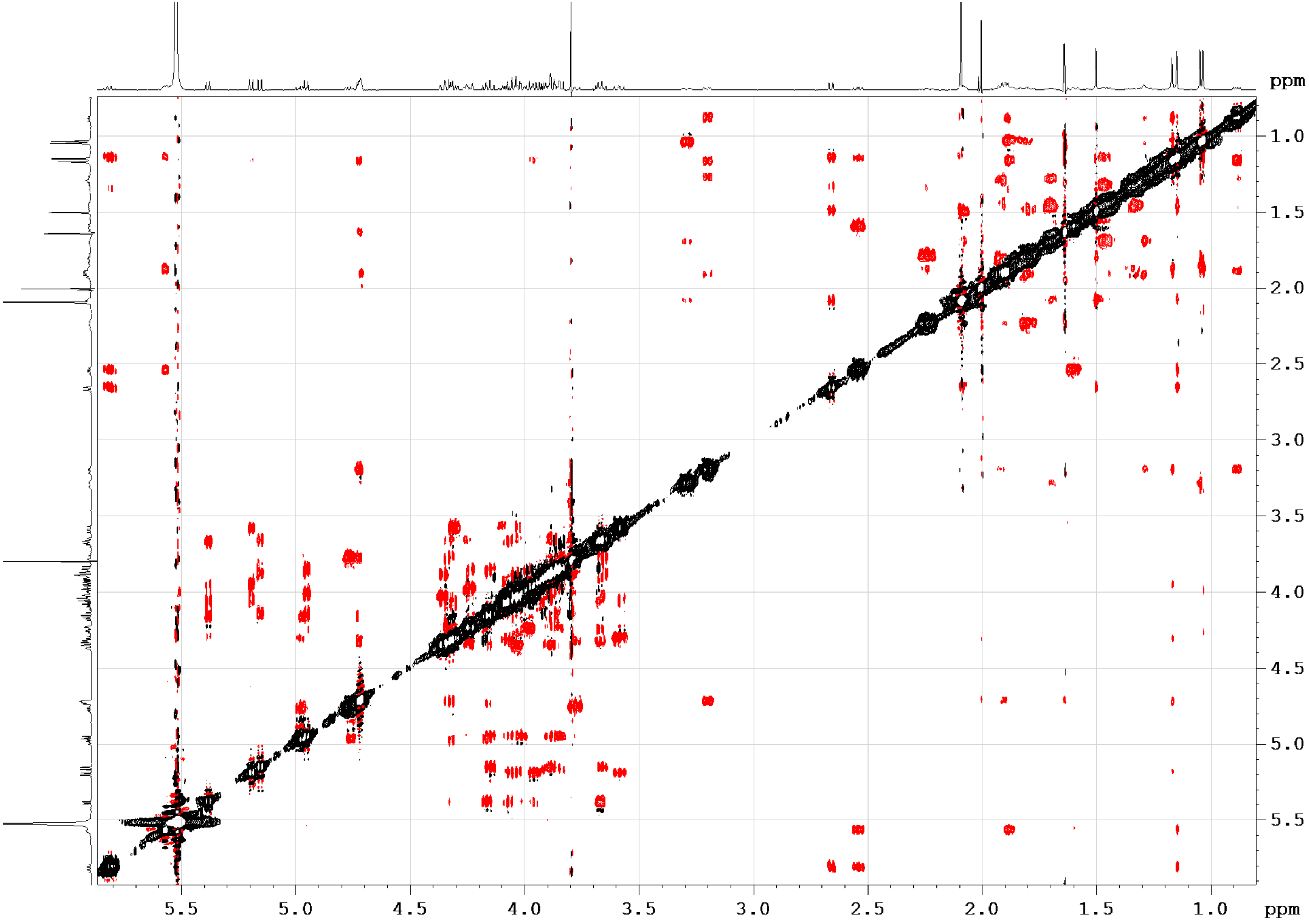

Figure S37. The ROESY (500.12 MHz) spectrum of okhotoside A2-1 (**5**) in C_5D_5N/D_2O (4/1)

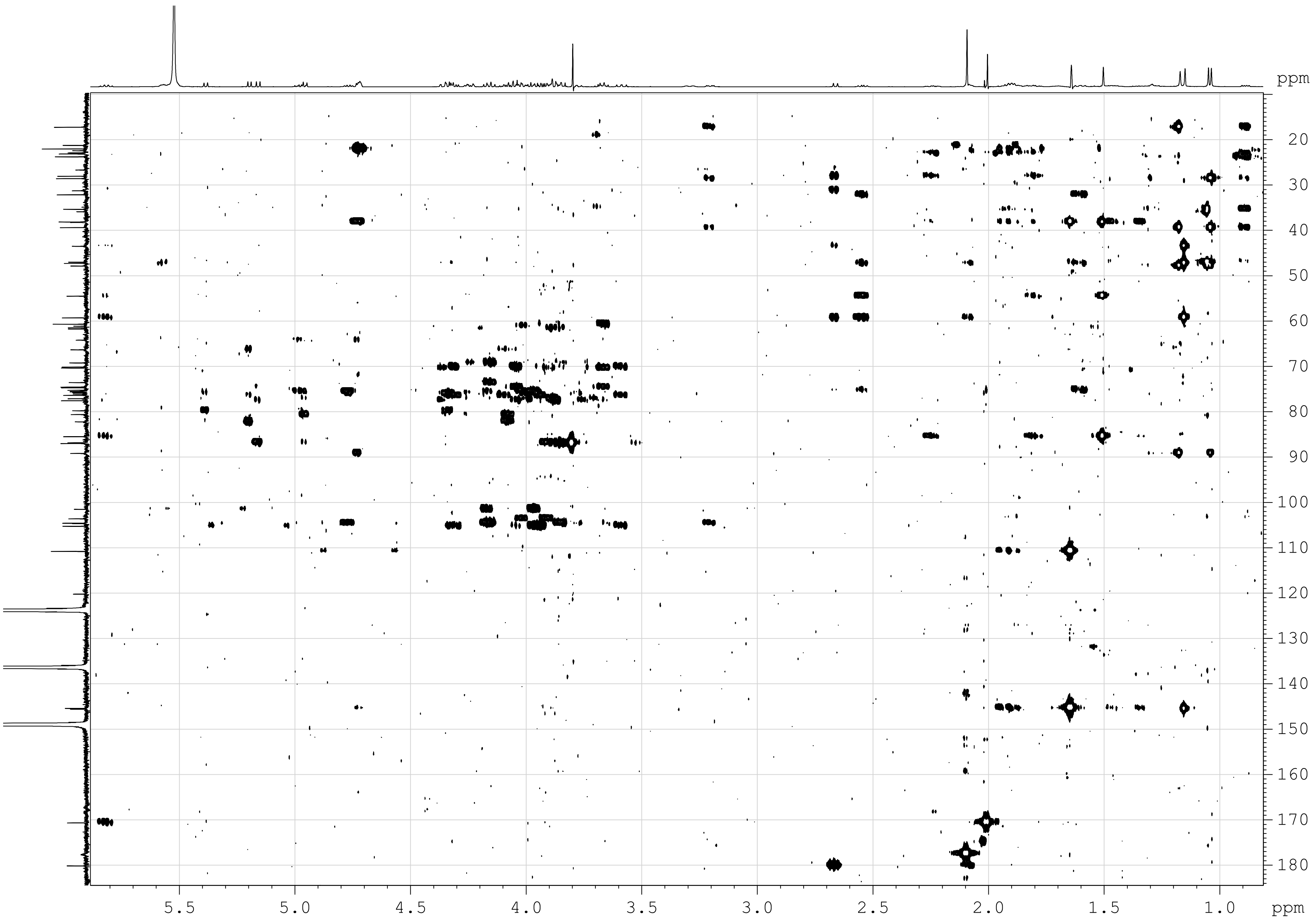

Figure S38. The HMBC (500.12 MHz) spectrum of okhotoside A2-1 (**5**) in C5D5N/D2O (4/1)

Table S4. ^{13}C and ^{1}H NMR chemical shifts and HMBC and ROESY correlations of aglycone moiety of cucumarioside A$_2$-5 (**6**).

Position	δC mult.[a]	δH mult. (*J* in Hz)[b]	HMBC	ROESY
1	35.8 CH$_2$	1.32 m		H-11
2	26.7 CH$_2$	1.94 m		
		1.78 m		
3	88.9 CH	3.19 dd (3.8; 12.6)		H-5, H-31, H1-Xyl1
4	39.3 C			
5	47.8 CH	0.90 dd (5.1; 10.0)	C: 19	H-3, H-31
6	23.1 CH$_2$	1.95 m		
		1.90 m		H-31
7	120.4 CH	5.57 m		H-15
8	145.4 C			
9	47.0 CH	3.27 brd (14.5)		H-19
10	35.4 C			
11	22.4 CH$_2$	1.72 m		H-1
		1.46 m		
12	31.0 CH$_2$	2.03 m		H-21
13	57.8 C			
14	47.4 C			
15	43.5 CH$_2$	2.62 dd (7.0; 11.7)	C: 12, 13, 14, 17	H-7
		1.48 dd (3.0; 11.7)		
16	76.1 CH	5.68 q (8.5)	OAc	H-32
17	55.1 CH	3.19 m	C: 13, 18, 21	H-12, H-21, H-32
18	180.0 C			
19	23.8 CH$_3$	1.09 s	C: 5, 9, 10	H-1, H-2, H-6, H-9
20	82.6 C			
21	29.4 CH$_3$	1.58 s	C: 17, 20, 22	H-12, H-17
22	52.6 CH	3.82 m	C: 20, 23	
		3.20 m	C: 17, 20, 23	
23	209.1 C			
24	51.5 CH$_2$	2.42 dd (5.5; 15.6)	C: 23, 25, 26, 27	
		2.24 ddd (7.8; 12.5; 15.6)	C: 23, 25, 26, 27	
25	24.3 CH	2.08 m	C: 26, 27	
26	22.4 CH$_3$	0.87 d (6.7)	C: 24, 25, 27	H-25
27	22.2 CH$_3$	0.83 d (6.7)	C: 24, 25, 26	H-25
30	17.3 CH$_3$	1.02 s	C: 3, 4, 5, 31	H-2, H-6, H-31
31	28.6 CH$_3$	1.18 s	C: 3, 4, 5, 30	H-3, H-5, H-6, H-30, H-1 Xyl1
32	32.0 CH$_3$	1.06 s	C: 8, 13, 14, 15	H-15, H-16, H-17
O<u>C</u>OCH$_3$	170.1 C			
OCO<u>C</u>H$_3$	21.3 CH$_3$	2.01 s	OAc	

[a] Recorded at 125.67 MHz in C_5D_5N/D_2O (4/1). [b] Recorded at 500.12 MHz in C_5D_5N/D_2O (4/1). The original spectra of **6** are provided as Figures S39–S44.

Table S5. ^{13}C and ^{1}H NMR chemical shifts and HMBC and ROESY correlations of carbohydrate moiety of cucumarioside A$_2$-5 (**6**).

Atom	δC mult.[a]	δH mult. (*J* in Hz)[b,c,d]	HMBC	ROESY
Xyl1 (1→C-3)				
1	104.6 CH	4.74 d (6.4)	C: 3	H-3; H-3, 5 Xyl1
2	**81.4** CH	3.98 t (8.4)	C: 1 Qui2; C: 1 Xyl1	
3	75.4 CH	4.32 t (8.4)	C: 2, 4 Xyl1	
4	*76.3* CH	5.00 dd (13.7; 8.4)	C: 3 Xyl1	H-2 Xyl1
5	64.1 CH$_2$	4.79 dd (11.6; 5.8)	C: 1, 3 Xyl1	
		3.85 t (10.0)		
Qui2 (1→2Xyl1)				
1	101.9 CH	5.20 d (8.5)	C: 2 Xyl1	H-2 Xyl1; H-5 Qui2
2	82.4 CH	3.95 m	C: 1, 3 Qui2	
3	75.3 CH	4.00 m	C: 2, 4 Qui2	H-1 Qui2
4	**86.0** CH	3.55 t (8.8)	C: 1 Glc3; C: 3, 5 Qui2	H-1 Glc3
5	71.0 CH	3.59 m		H-1 Qui2
6	17.9 CH$_3$	1.59 d (5.8)		
Glc3 (1→4Qui2)				
1	104.0 CH	4.86 d (7.8)	C: 4 Qui2	H-4 Qui2; H-3, 5 Glc3
2	73.6 CH	3.93 t (8.4)	C: 1 Glc3	
3	**86.8** CH	4.21 t (8.4)	C: 1 MeGlc4; C: 4 Glc3	H-1 MeGlc4
4	69.3 CH	3.90 m	C: 3 Glc3	
5	77.1 CH	3.90 m	C: 6 Glc3	H-1 Glc3
6	61.6 CH$_2$	4.29 brd (12.0)		
		4.01 brd (12.0)	C: 5 Glc3	
MeGlc4 (1→3Glc3)				
1	104.6 CH	5.22 d (8.9)	C: 3 Glc3	H-3 Glc3; H-3, 5 MeGlc4
2	74.6 CH	3.87 t (8.9)	C: 1, 3 MeGlc4	
3	87.0 CH	3.69 t (8.9)	OMe; C: 2, 4 MeGlc4	H-1 MeGlc4; Ome
4	70.4 CH	3.89 m	C: 5 MeGlc4	
5	77.6 CH	3.93 m		
6	61.8 CH$_2$	4.37 d (11.0)		
		4.05 dd (11.0; 4.8)	C: 5 MeGlc4	
OMe	60.6 CH$_3$	3.81 s	C: 3 MeGlc4	
Xyl5 (1→2Qui2)				
1	105.3 CH	5.19 d (7.4)	C: 2 Qui2	H-2 Qui2; H-3,5 Xyl5
2	74.8 CH	3.92 t (8.5)	C: 1, 3 Xyl5	
3	76.3 CH	4.01 t (9.4)	C: 2, 4 Xyl5	H-1 Xyl5
4	70.0 CH	4.08 m		
5	66.4 CH$_2$	4.31 m	C: 4 Xyl5	H-1 Xyl5
		3.59 m		

[a] Recorded at 25.67 MHz in C_5D_5N/D_2O. [b] Recorded at 500.12 MHz in C_5D_5N/D_2O. [c] Bold = interglycosidic positions. [d] Italic – sulfate positions. The original spectra of **6** are provided as Figures S39–S44.

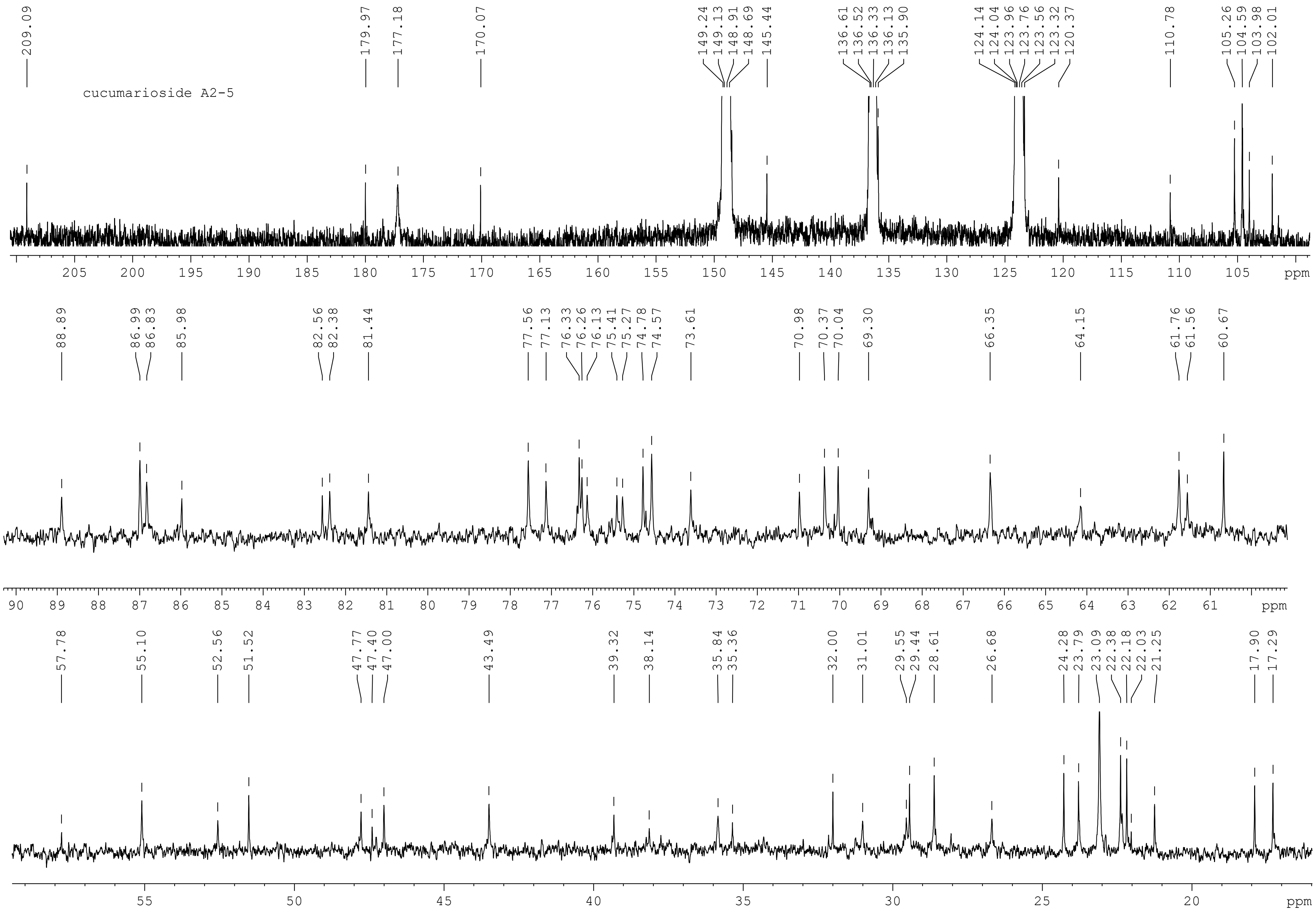

Figure S39. The ^{13}C NMR (125.67 MHz) spectrum of cucumarioside A_2-5 (**6**) in C_5D_5N/D_2O (4/1)

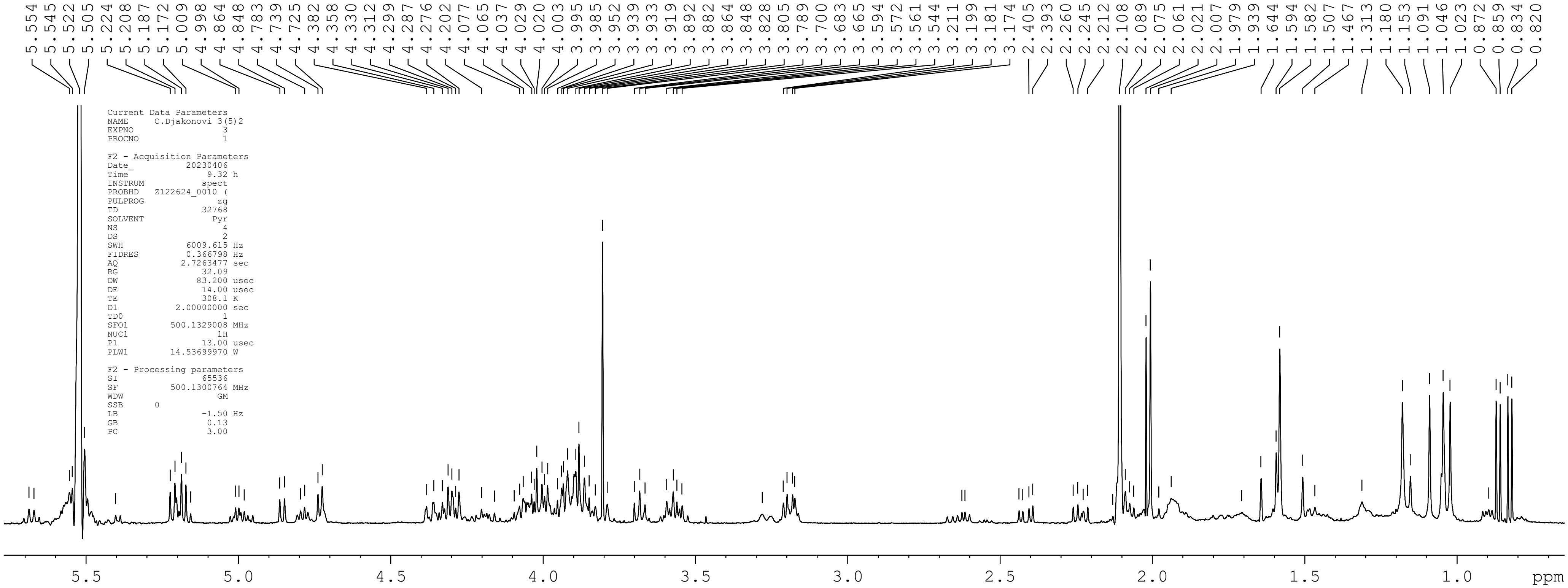

Figure S40. The ^{1}H NMR (500.12 MHz) spectrum of cucumarioside A$_2$-5 (**6**) in C_5D_5N/D_2O (4/1)

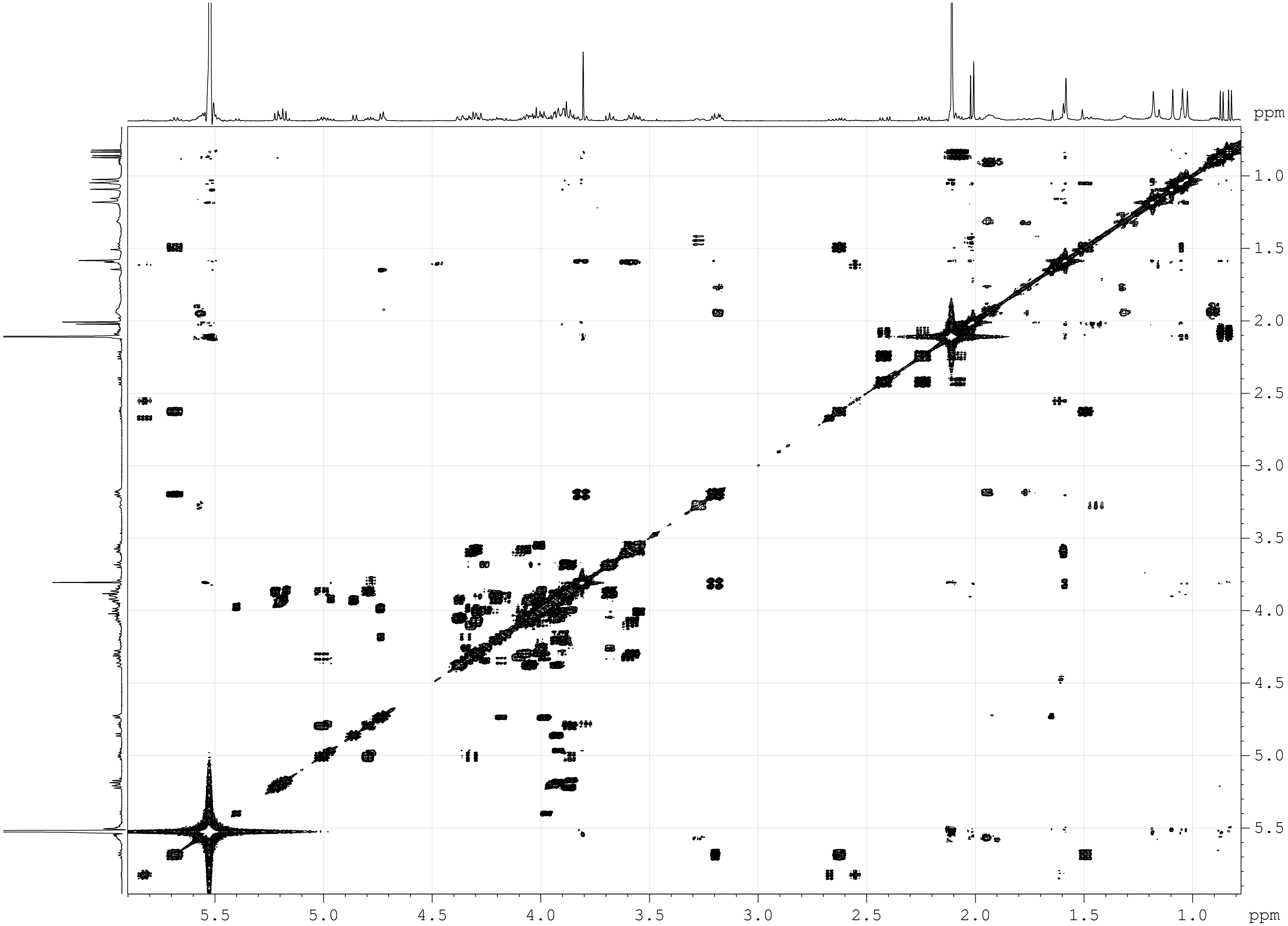

Figure S41. The COSY (500.12 MHz) spectrum of cucumarioside A_2-5 (**6**) in C_5D_5N/D_2O (4/1)

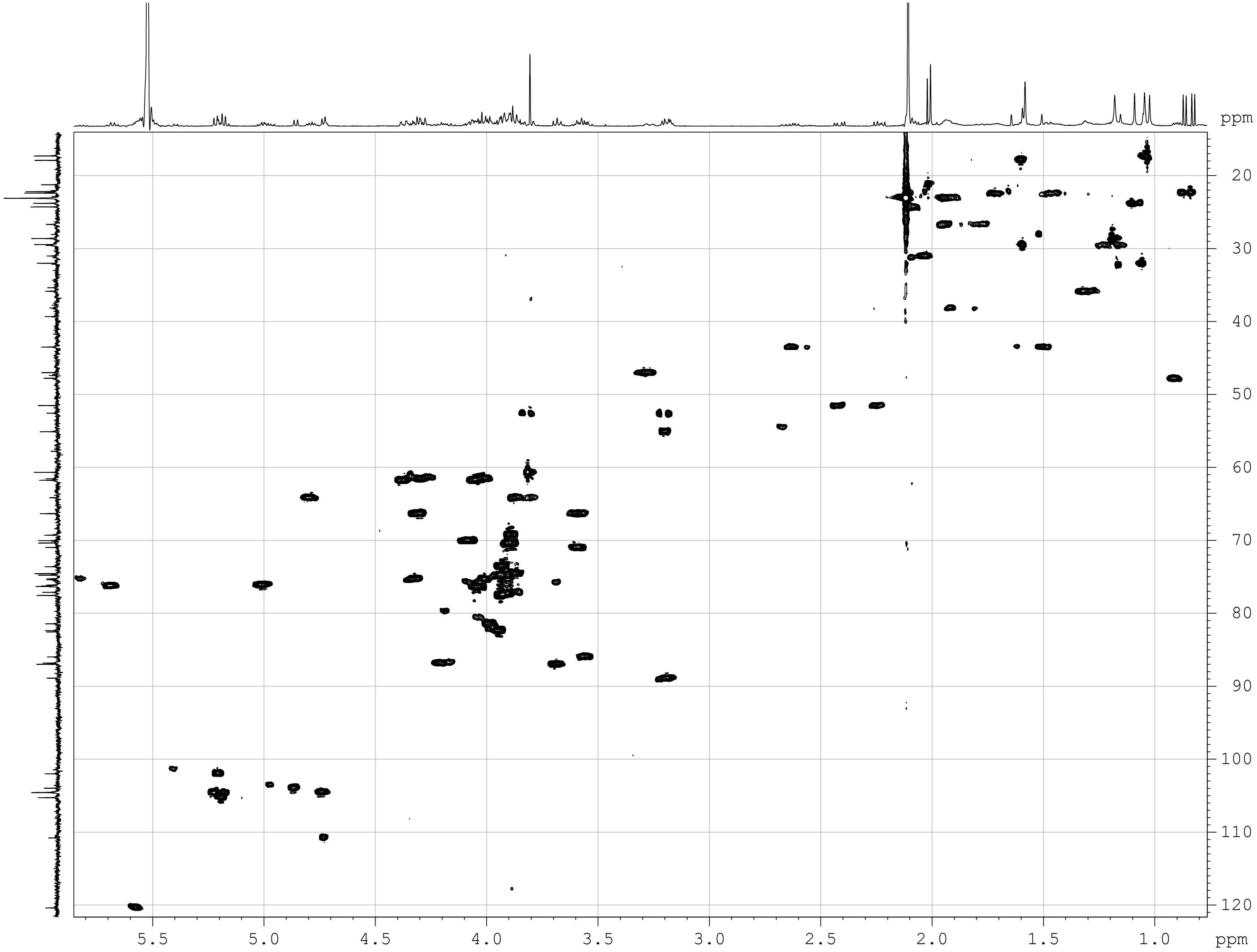

Figure S42. The HSQC (500.12 MHz) spectrum of cucumarioside A$_2$-5 (**6**) in C_5D_5N/D_2O (4/1)

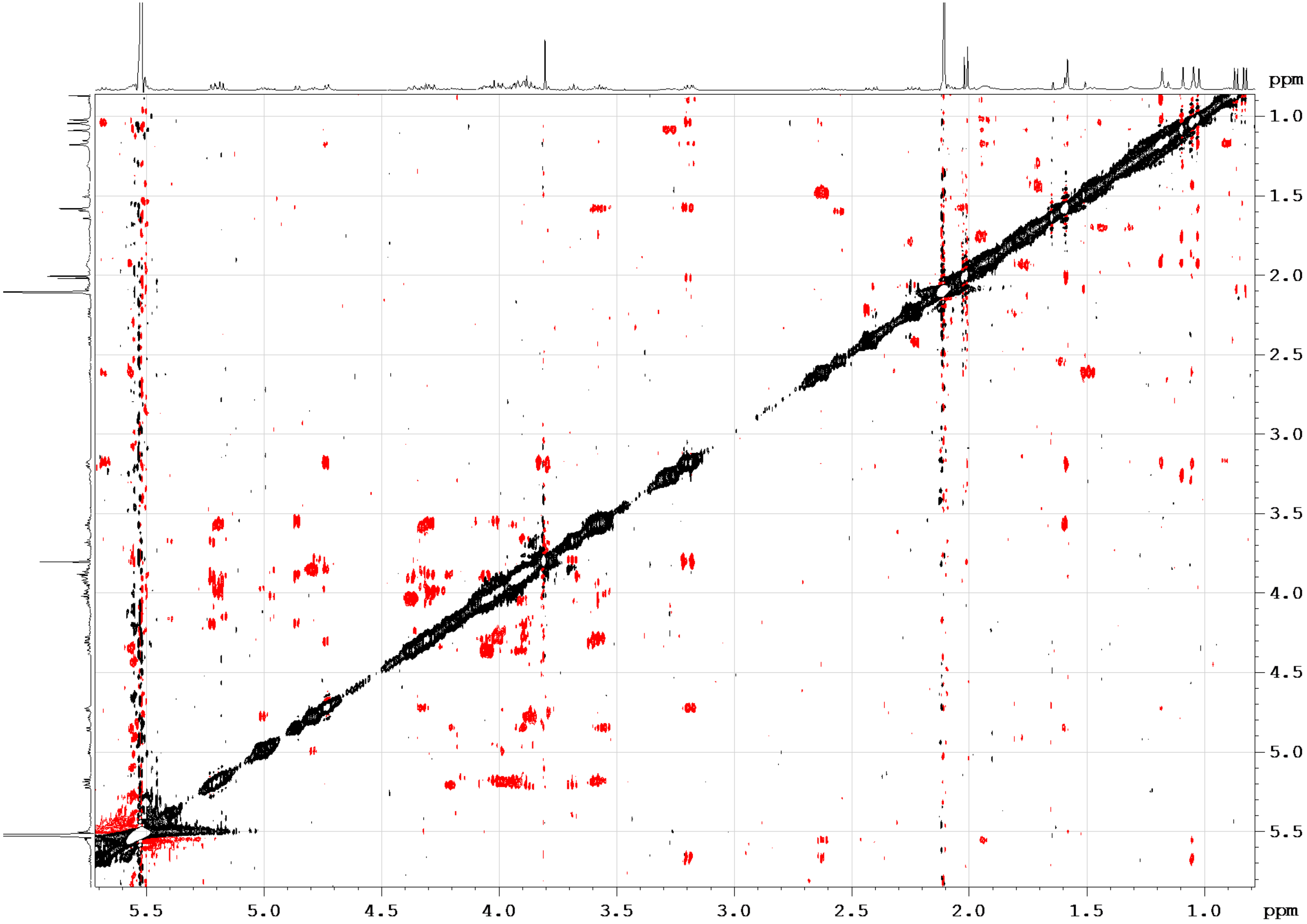

Figure S43. The ROESY (500.12 MHz) spectrum of cucumarioside A_2-5 (**6**) in C_5D_5N/D_2O (4/1)

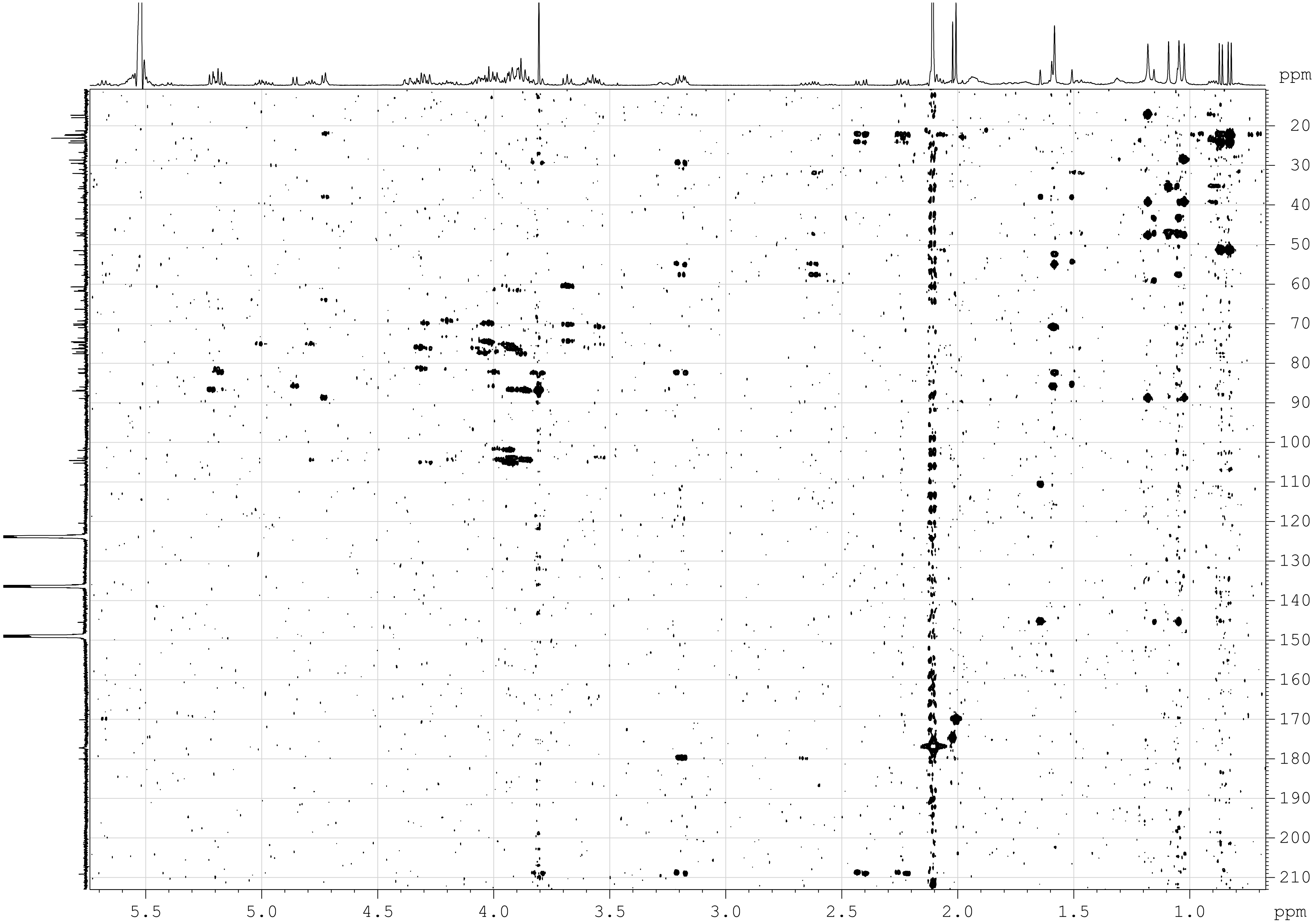

Figure S44. The HMBC (500.12 MHz) spectrum of cucumarioside A_2-5 (**6**) in C_5D_5N/D_2O (4/1)

Table S6. ^{13}C NMR chemical shifts of frondoside A_2-3 (**7**).

**Position**	**δ_C mult.**[a]	**Position**	**δ_C mult.**[a]
1	36.0 CH_2	2	81.4 CH
2	26.7 CH_2	3	75.4 CH
3	89.0 CH	4	76.1 CH
4	39.4 C	5	64.1 CH_2
5	48.0 CH	Qui2 (1→2Xyl1)	
6	23.2 CH_2	1	102.0 CH
7	120.0 CH	2	82.4 CH
8	146.7 C	3	75.3 CH
9	47.3 CH	4	86.0 CH
10	35.3 C	5	71.0 CH
11	22.7 CH_2	6	17.9 CH_3
12	30.1 CH_2	Glc3 (1→4Qui2)	
13	58.7 C	1	104.0 CH
14	46.1 C	2	73.6 CH
15	34.1 CH_2	3	86.8 CH
16	23.1 CH_2	4	69.3 CH
17	53.1 CH	5	77.1 CH
18	180.9 C	6	61.5 CH_2
19	23.9 CH_3	MeGlc4 (1→3Glc3)	
20	84.4 C	1	104.6 CH
21	26.1 CH_3	2	74.6 CH
22	42.0 CH_2	3	87.0 CH
23	119.8 CH	4	70.4 CH
24	143.7 CH	5	77.5 CH
25	66.6 C	6	61.8 CH_2
26	29.9 CH_3	OMe	60.7 CH_3
27	29.8 CH_3	Xyl5 (1→2Qui2)	
30	17.3 CH_3	1	105.2 CH
31	28.7 CH_3	2	74.7 CH
32	30.7 CH_3	3	76.3 CH
Xyl1 (1→C-3)		4	70.0 CH
1	104.6 CH	5	66.3 CH_2

[a] Recorded at 125.67 MHz in C_5D_5N/D_2O (4.1).

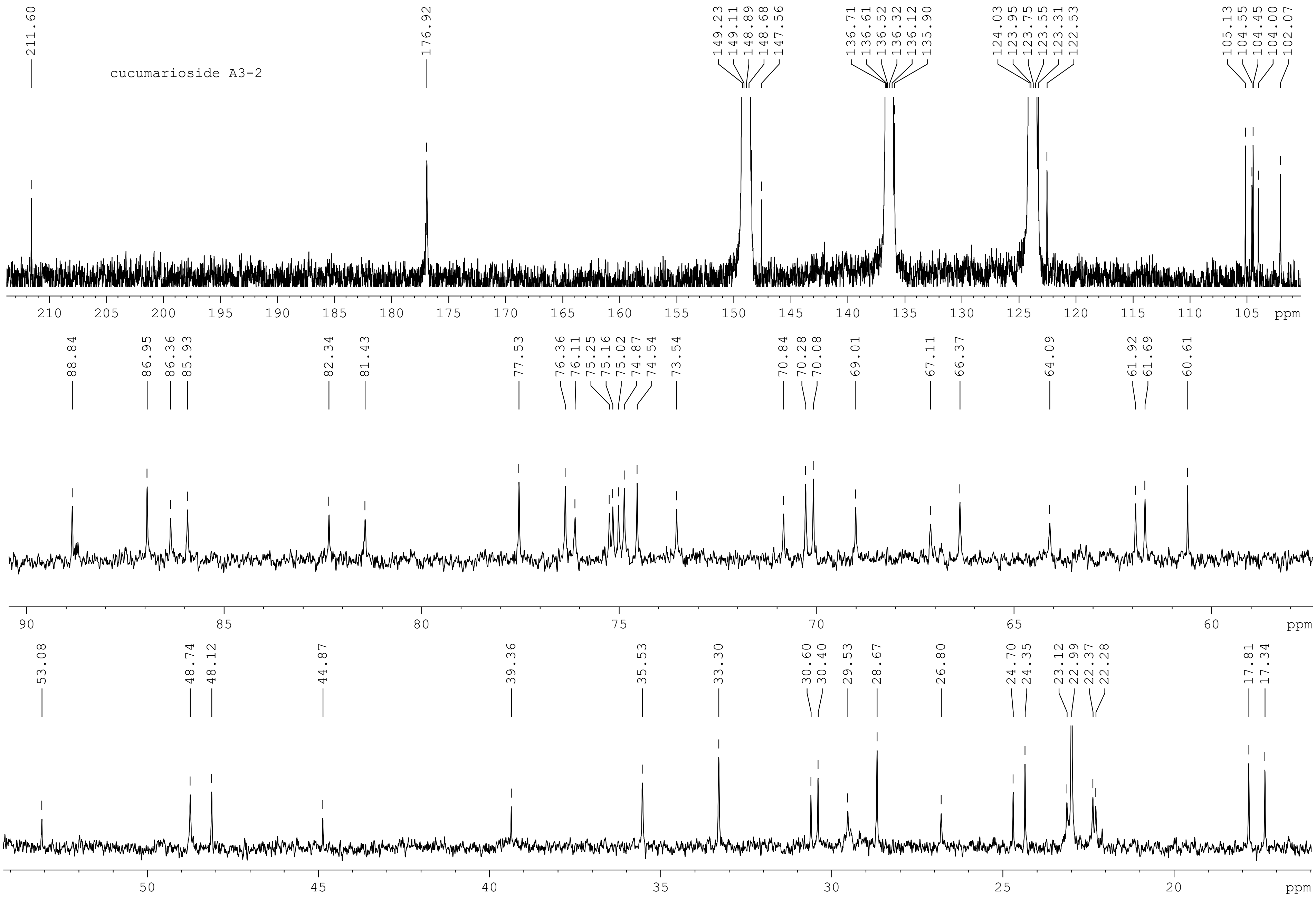

Figure S45. The ^{13}C NMR (125.67 MHz) spectrum of cucumarioside A$_3$-2 (**8**) in C_5D_5N/D_2O (4/1)

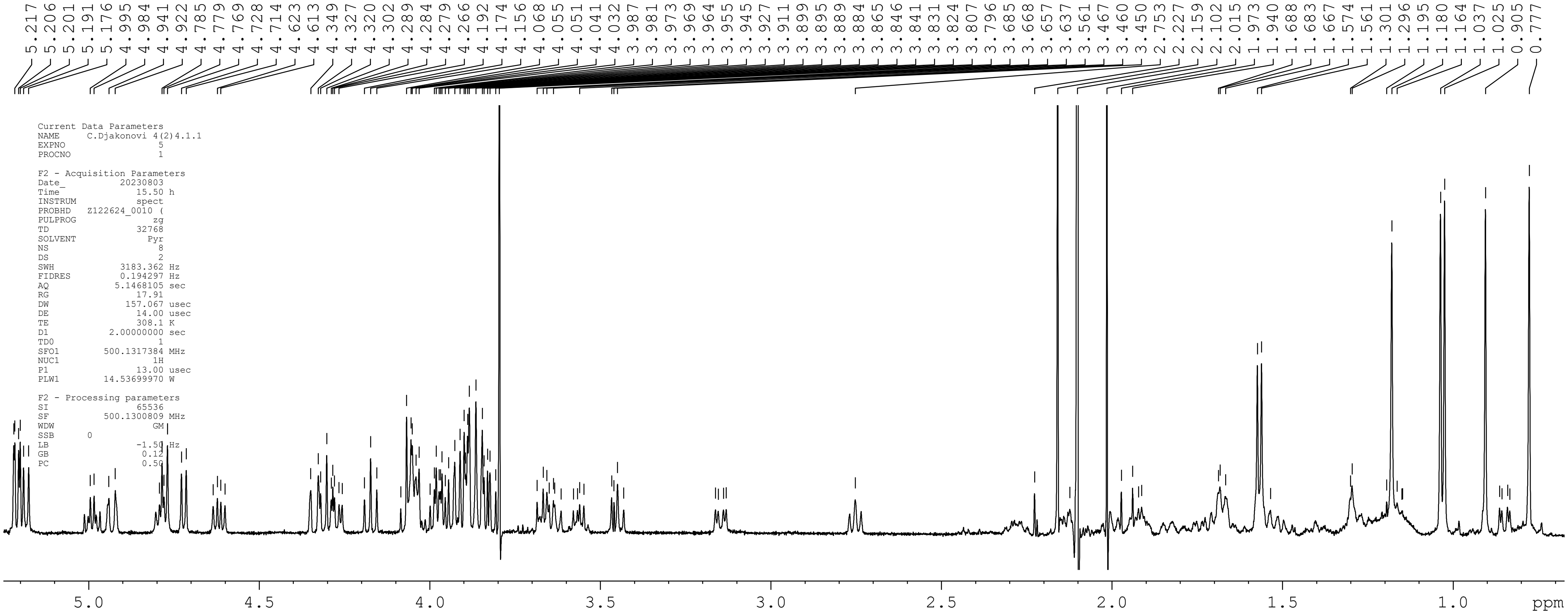

Figure S46. The ^{1}H NMR (500.12 MHz) spectrum of cucumarioside A3-2 (**8**) in C_5D_5N/D_2O (4/1)

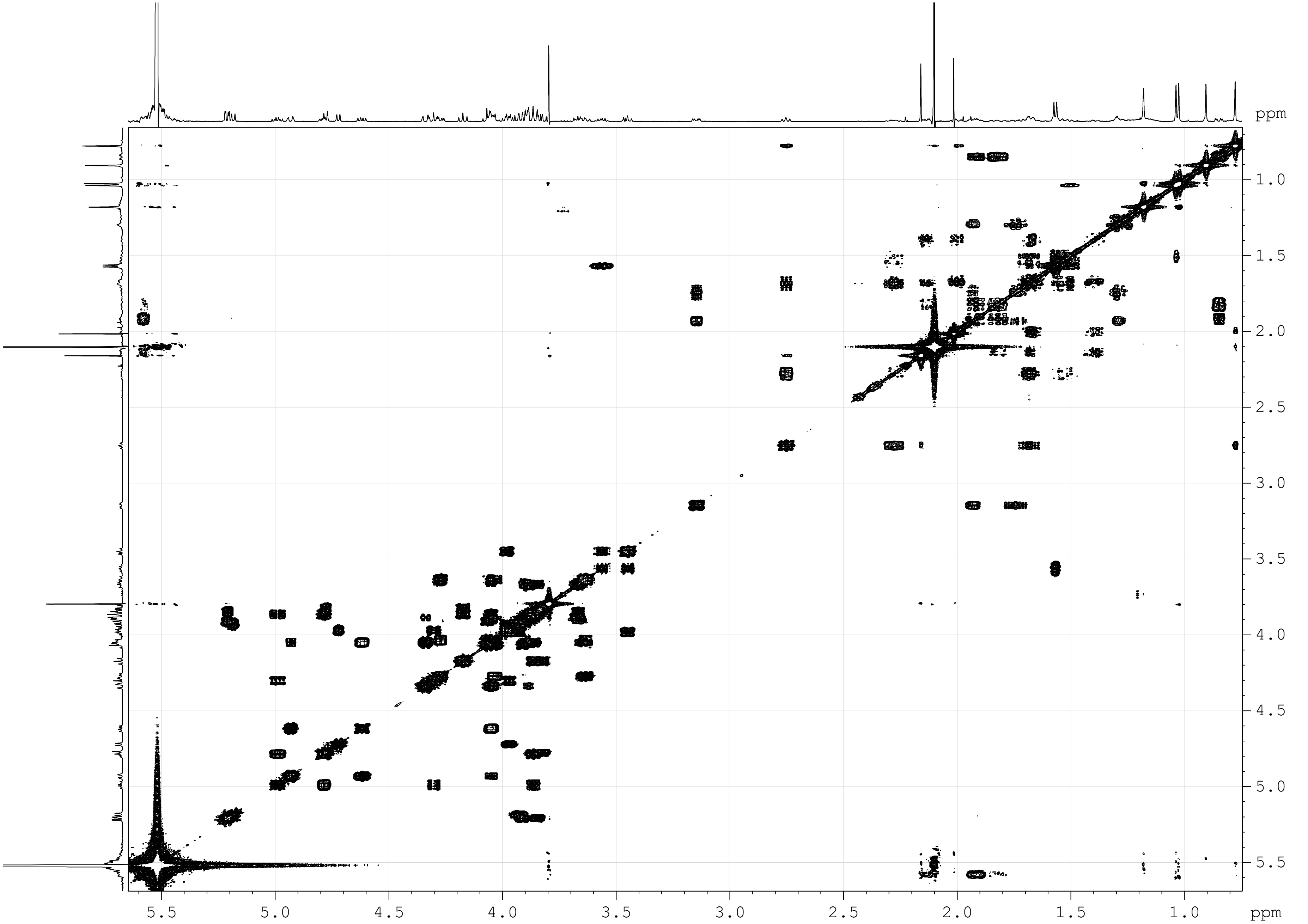

Figure S47. The COSY (500.12 MHz) spectrum of cucumarioside A_3-2 (**8**) in C_5D_5N/D_2O (4/1)

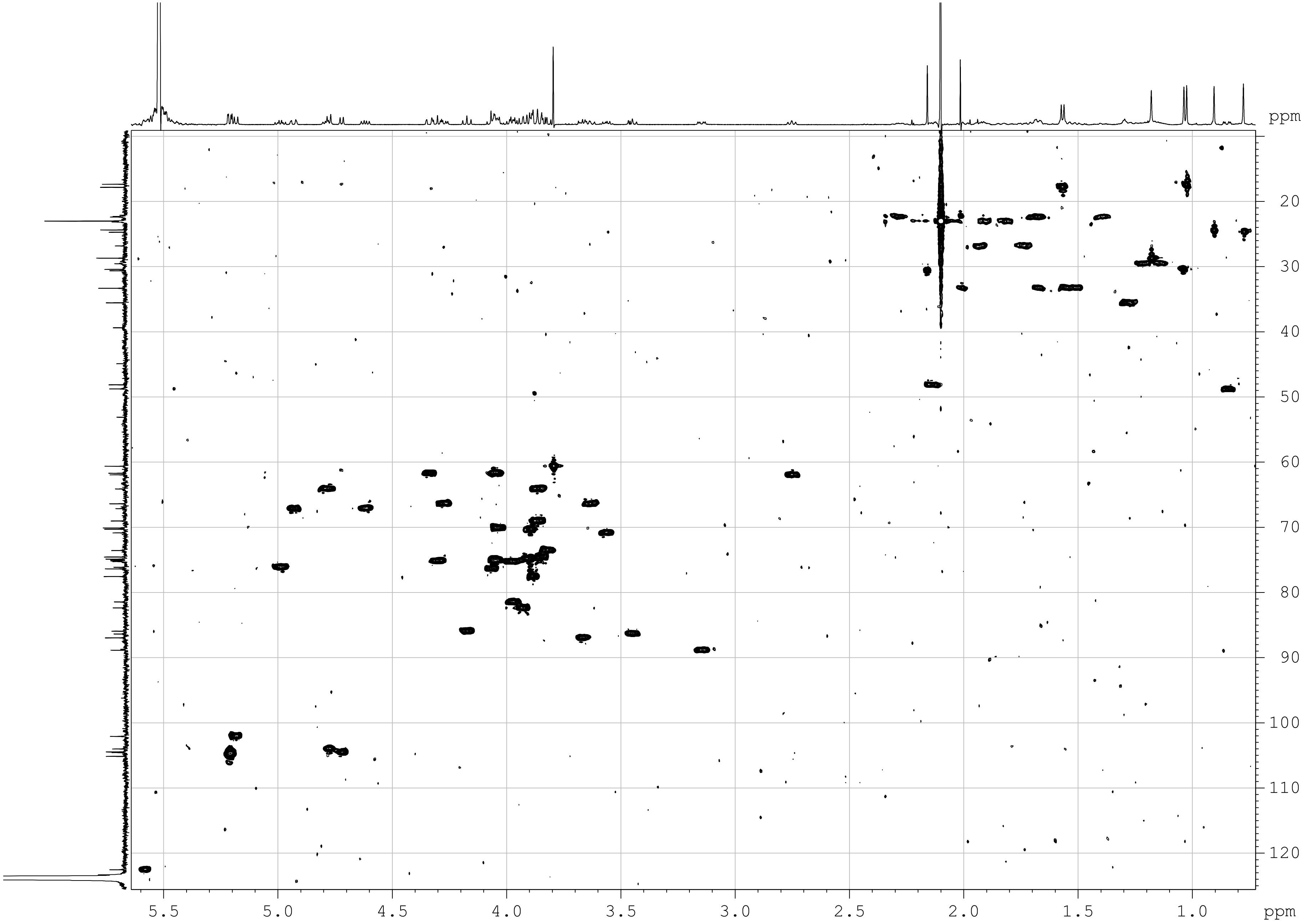

Figure S48. The HSQC (500.12 MHz) spectrum of cucumarioside A_3-2 (**8**) in C_5D_5N/D_2O (4/1)

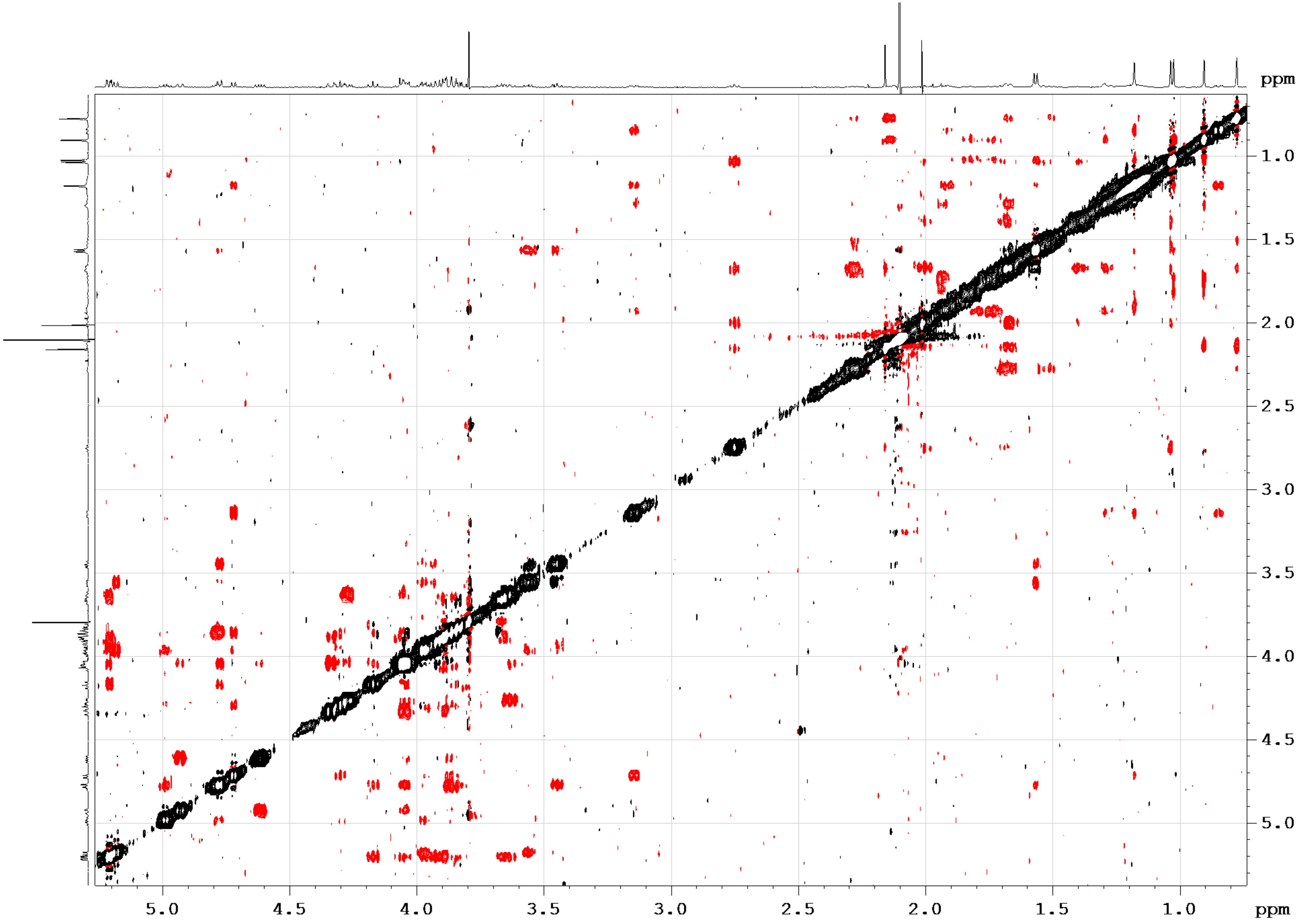

Figure S49. The ROESY (500.12 MHz) spectrum of cucumarioside A_3-2 (**8**) in C_5D_5N/D_2O (4/1)

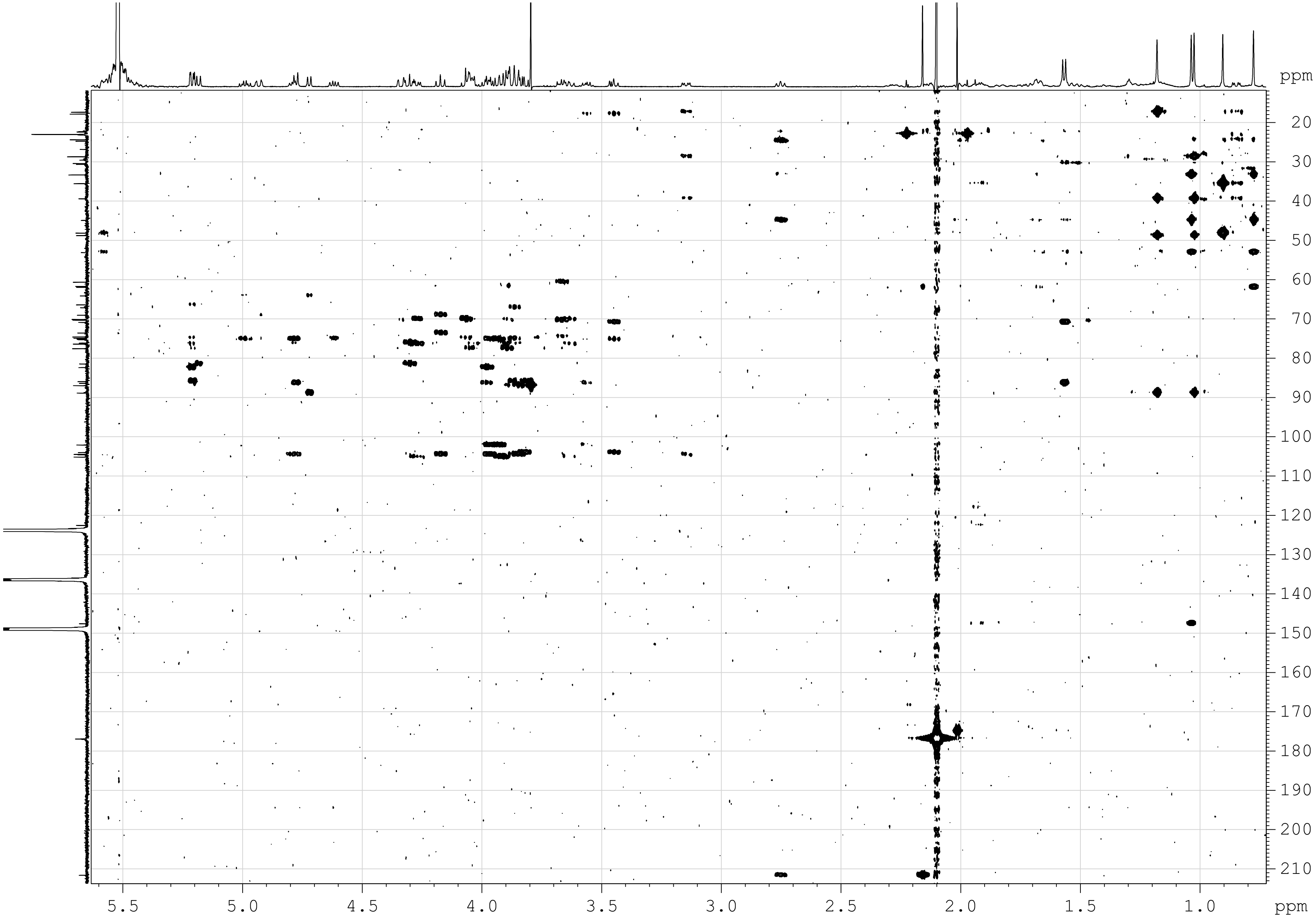

Figure S50. The HMBC (500.12 MHz) spectrum of cucumarioside A3-2 (**8**) in C_5D_5N/D_2O (4/1)

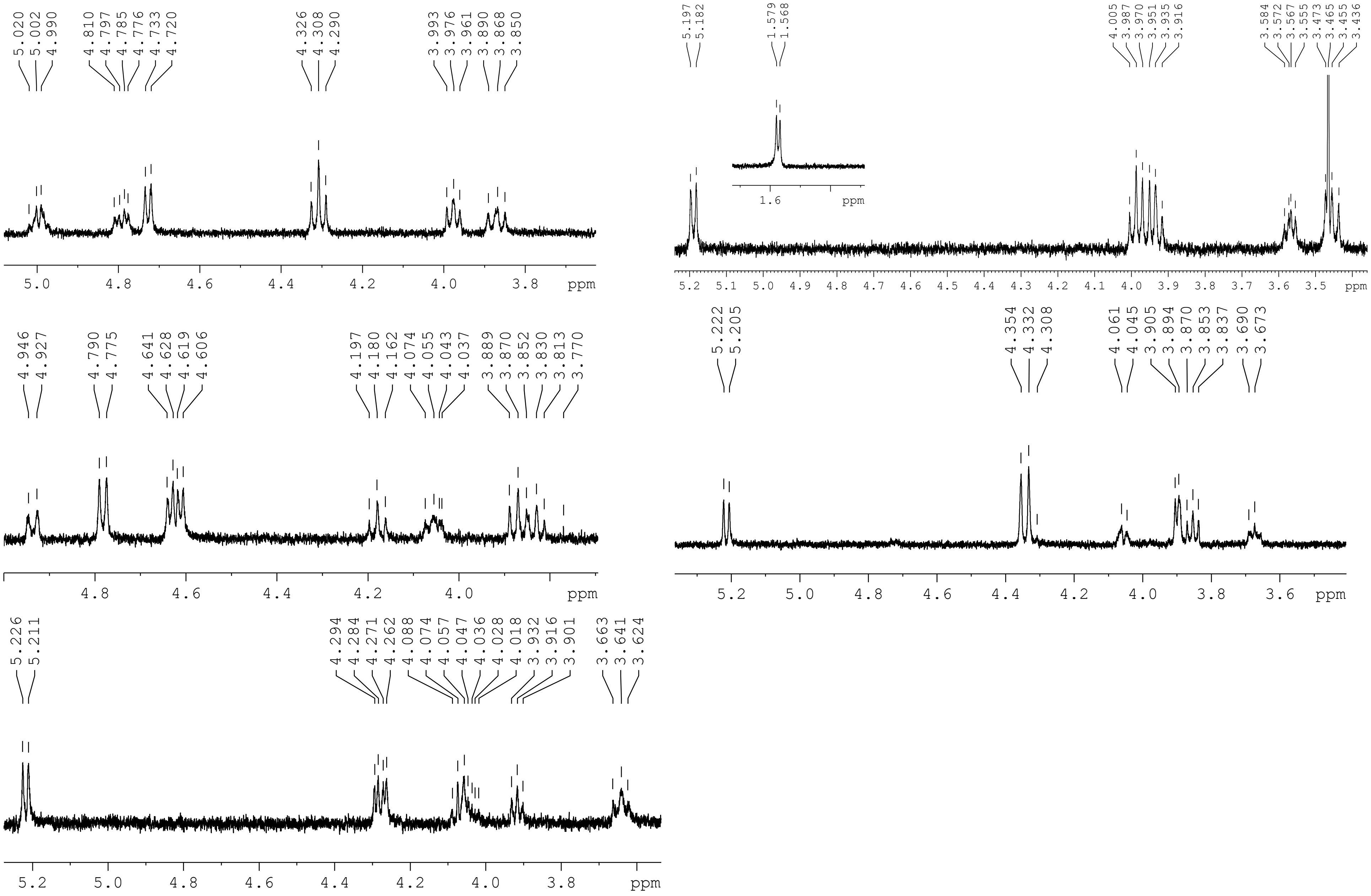

Figure S51. 1D TOCSY (500.12 MHz) spectra of Xyl1, Qui2, Glc3, MeGlc4, Xyl5 of cucumarioside A3-2 (**8**) in C_5D_5N/D_2O (4/1)

Table S7. ^{13}C and ^{1}H NMR chemical shifts and HMBC and ROESY correlations of carbohydrate moiety of isokoreoside A (**9**).

Atom	δC mult.[a]	δH mult. (*J* in Hz)[b,c,d]	HMBC	ROESY
Xyl1 (1→C-3)				
1	104.6 CH	4.73 d (6.9)	C: 3; C: 3, 5 Xyl1	H-3; H-3, 5 Xyl1
2	**81.5** CH	3.97 dd (6.9; 9.3)	C: 1 Qui2; C: 1 Xyl1	H-4 Xyl1; H-1 Qui2
3	75.1 CH	4.31 t (9.3)	C: 2, 4 Xyl1	H-1, 5 Xyl1
4	*76.1* CH	4.99 dd (8.3; 13.7)	C: 3 Xyl1	
5	64.1 CH_2	4.79 dd (4.4; 11.3)	C: 1 Xyl1	
		3.88 dd (8.3; 11.3)		H-1, 3 Xyl1
Qui2 (1→2Xyl1)				
1	102.1 CH	5.19 d (7.5)	C: 2 Xyl1	H-2 Xyl1; H-3, 5 Qui2
2	**82.4** CH	3.95 t (8.2)	C: 1 Xyl5, C: 1, 3 Qui2	H-1 Xyl5, H-4 Qui2
3	75.2 CH	3.99 t (8.2)	C: 2, 4 Qui2	
4	**86.3** CH	3.47 t (8.2)	C: 1 Glc3; C: 3, 5 Qui2	H-1 Glc3; H-2 Qui2
5	70.9 CH	3.58 dd (6.0; 8.2)	C: 4 Qui2	H-1, 3 Qui2
6	17.8 CH_3	1.58 d (6.0)	C: 4, 5 Qui2	
Glc3 (1→4Qui2)				
1	103.9 CH	4.78 d (7.8)	C: 4 Qui2	H-4 Qui2; H-5 Glc3
2	73.5 CH	3.81 t (9.6)	C: 1, 3 Glc3	
3	**86.5** CH	4.12 t (9.6)	C: 1 MeGlc4; C: 2, 4	H-1 MeGlc4; H-1 Glc3
4	69.1 CH	3.81 t (9.6)	C: 5, 6 Glc3	
5	75.5 CH	4.07 m	C: 4 MeGlc4	H-1 Glc3
6	*67.3* CH_2	4.95 d (10.5)		
		4.60 dd (6.1; 11.3)	C: 5 Glc3	H-4 Glc3
MeGlc4 (1→3Glc3)				
1	104.8 CH	5.15 d (7.9)	C: 3 Glc3	H-3 Glc3; H-3, 5 MeGlc4
2	74.3 CH	3.78 t (8.7)	C: 1 MeGlc4	H-4 MeGlc4
3	86.3 CH	3.64 t (8.7)	OMe; C: 2, 4 MeGlc4	H-1, 5 MeGlc4; OMe
4	69.8 CH	4.01 m	C: 5 MeGlc4	
5	75.5 CH	4.00 m		H-1 MeGlc4
6	*67.0* CH_2	4.93 d (11.3)	C: 4 MeGlc4	
		4.75 dd (3.8; 11.3)		
OMe	60.5 CH_3	3.76 s	C: 3 MeGlc4	
Xyl5 (1→2Qui2)				
1	105.1 CH	5.23 d (7.0)	C: 2 Qui2	H-2 Qui2; H-3, 5 Xyl5
2	74.9 CH	3.92 t (8.2)	C: 1, 3 Xyl5	
3	76.4 CH	4.08 t (8.2)	C: 4 Xyl5	H-1 Xyl5
4	70.1 CH	4.06 m		
5	66.4 CH_2	4.29 dd (5.1; 11.4)	C: 3, 4 Xyl5	
		3.66 brt (10.1)	C: 3 Xyl5	H-1 Xyl5

[a] Recorded at 125.67 MHz in C_5D_5N/D_2O. [b] Recorded at 500.12 MHz in C_5D_5N/D_2O. [c] Bold = interglycosidic positions.[d] Italic – sulfate positions. Multiplicity by 1D TOCSY. The original spectra of **9** are provided as Figures S51–S56.

Table S8. ^{13}C and ^{1}H NMR chemical shifts, HMBC and ROESY correlations of aglycone moiety of isokoreoside A (**9**).

Position	δ_C mult.[a]	δ_H mult. (*J* in Hz)[b]	HMBC	ROESY
1	36.2 CH_2	1.63 m		H-11
		1.28 m		H-3, H-11
2	26.7 CH_2	2.00 m		
		1.77 m		H-19, H-30
3	88.6 CH	3.11 dd (4.1; 11.4)	C: 4, 30, 31, C: 1 Xyl1	H-1, H-5, H-31, H1-Xyl1
4	39.6 C			
5	52.8 CH	0.77 brd (12.0)	C: 10, 19, 30	H-3, H-31
6	21.1 CH_2	1.60 m		
		1.32 m		
7	28.3 CH_2	1.55 m		
8	41.5 CH	2.02 m		H-15, H-18, H-19
9	148.5 C			
10	39.2 C			
11	114.1 CH	5.20 m	C: 8, 10, 13	H-1
12	35.9 CH_2	2.29 brd (16.5)		H-17, H-32
		1.90 brdd (6.2; 16.5)	C: 9, 11, 13, 14, 18	H-18, H-21
13	46.1 C			
14	47.5 C			
15	33.9 CH_2	1.35 m		H-8, H-18, H-32
16	21.8 CH_2	2.34 m	C: 14, 17	
		1.61 m		
17	59.8 CH	2.95 t (8.9)	C: 12, 13, 16, 18, 20	H-12, H-21, H-32
18	16.4 CH_3	0.51 s	C: 12, 13, 14, 17	H-8, H-12, H-15, H-16, H-19, H-
19	22.1 CH_3	0.92 s	C: 1, 5, 9, 10	H-1, H-2, H-6, H-8
20	211.9 C			
21	31.0 CH_3	2.17 s	C: 17, 20	H-12, H-17, H-18
30	16.6 CH_3	0.99 s	C: 3, 4, 5, 31	H-2, H-6, H-31
31	28.0 CH_3	1.17 s	C: 3, 4, 5, 30	H-3, H-5, H-6
32	18.6 CH_3	0.75 s	C: 8, 13, 14, 15	H-12, H-15, H-17

[a] Recorded at 125.67 MHz in C_5D_5N/D_2O (4/1). [b] Recorded at 500.12 MHz in C_5D_5N/D_2O (4/1). The original spectra of **9** are provided as Figures S51–S56.

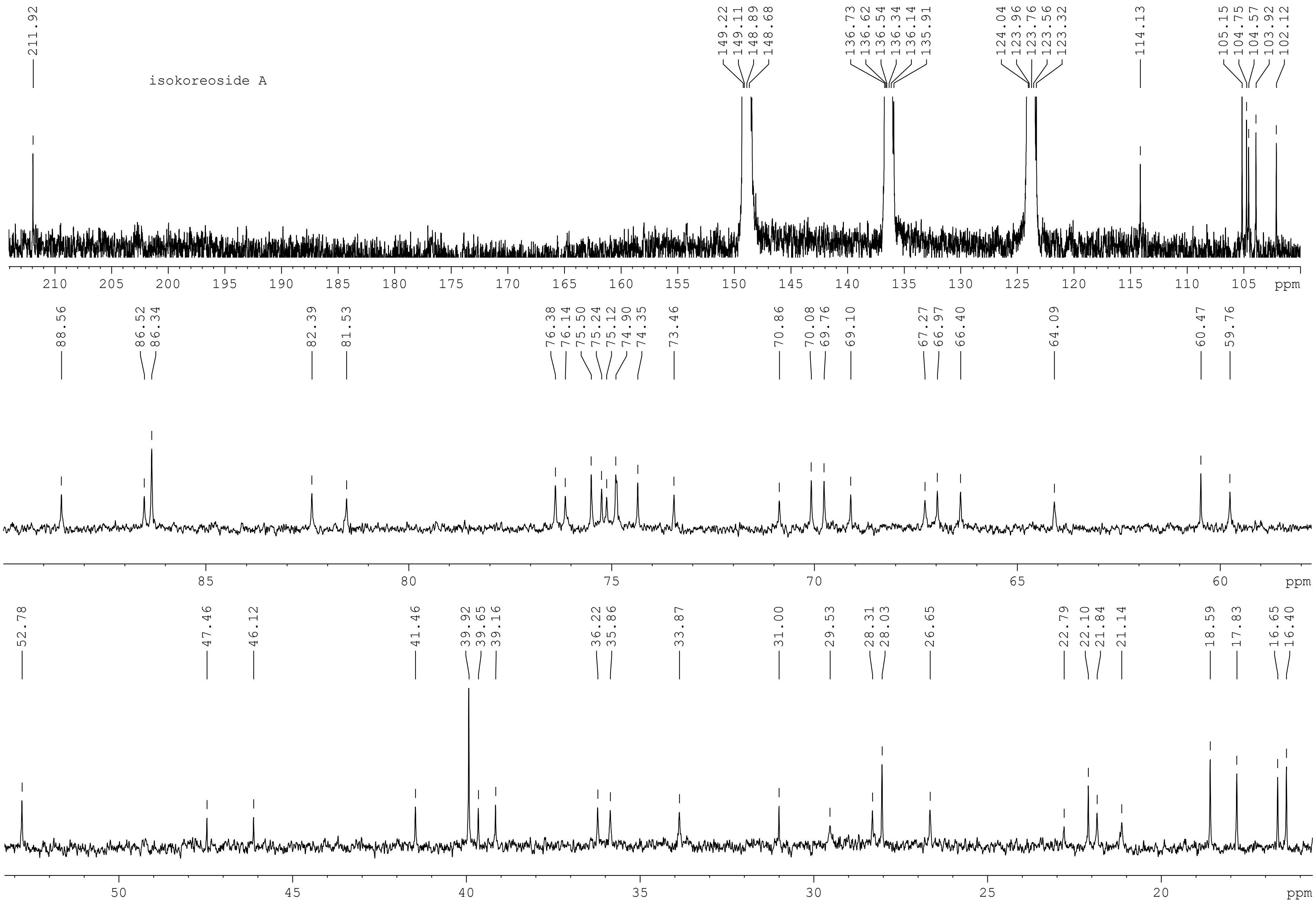

Figure S52. The ^{13}C NMR (125.67 MHz) spectrum of isokoreoside A (**9**) in C_5D_5N/D_2O (4/1)

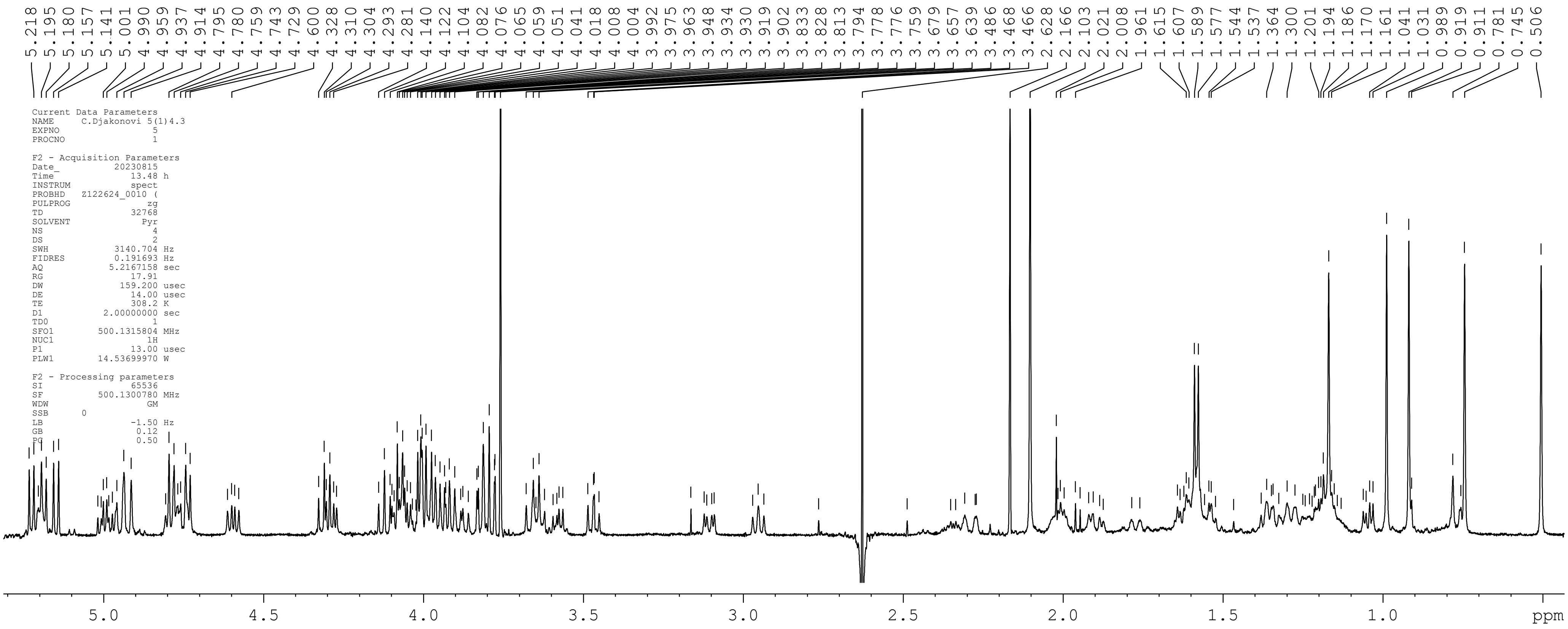

Figure S53. The ¹H NMR (500.12 MHz) spectrum of isokoreoside A (**9**) in C_5D_5N/D_2O (4/1)

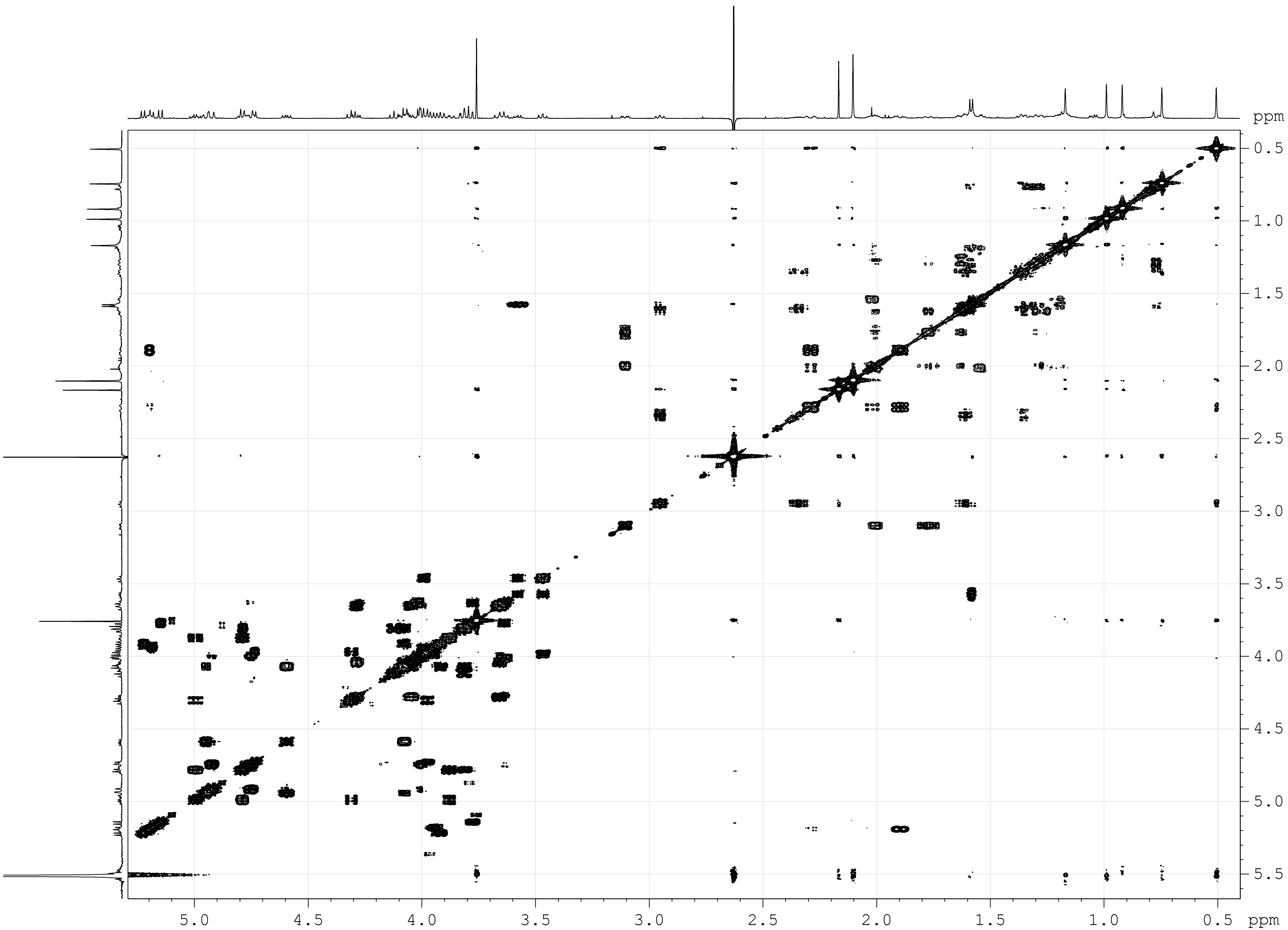

Figure S54. The COSY (500.12 MHz) spectrum of isokoreoside A (**9**) in C_5D_5N/D_2O (4/1)

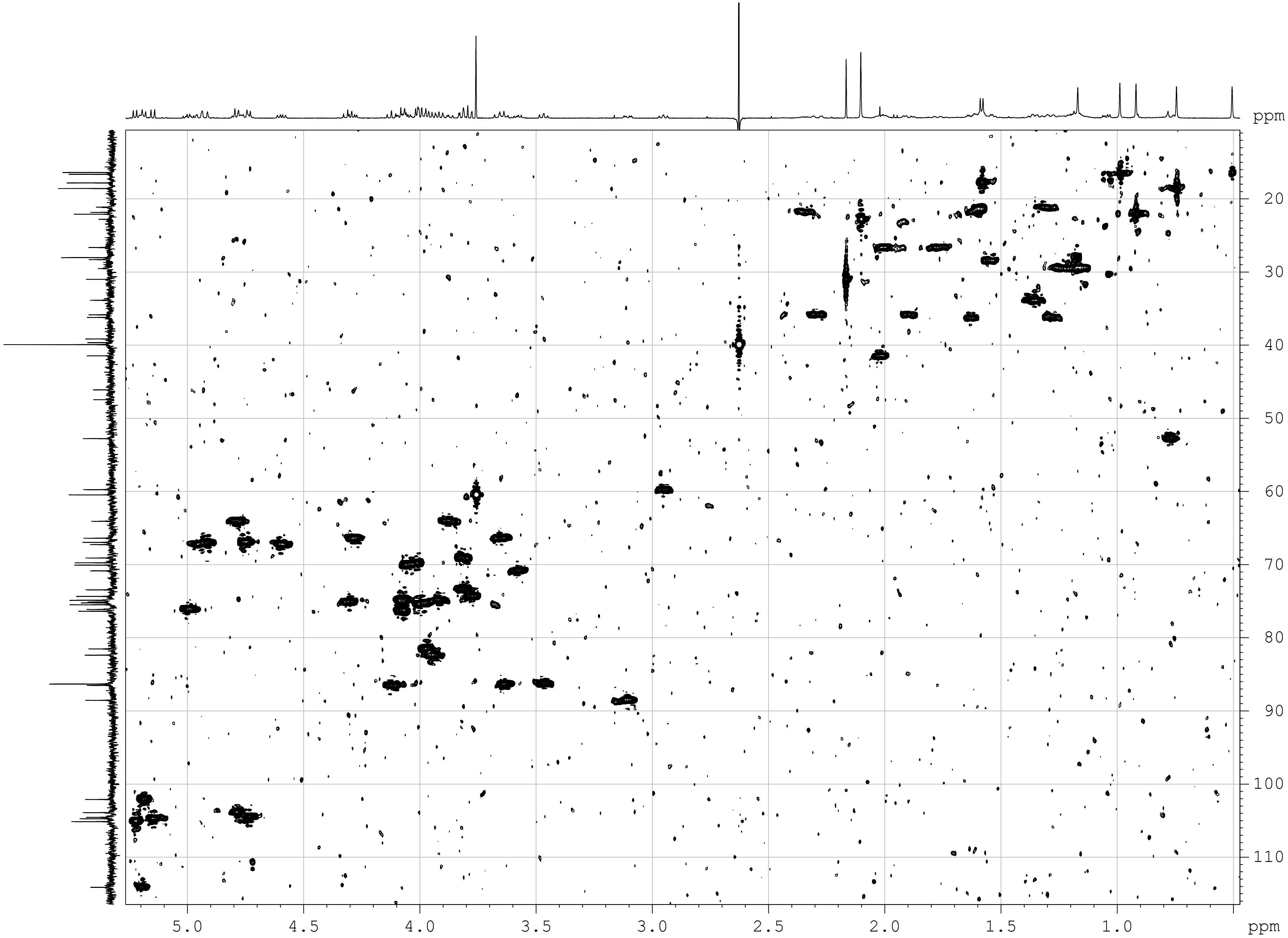

Figure S55. The HSQC (500.12 MHz) spectrum of isokoreoside A (**9**) in C_5D_5N/D_2O (4/1)

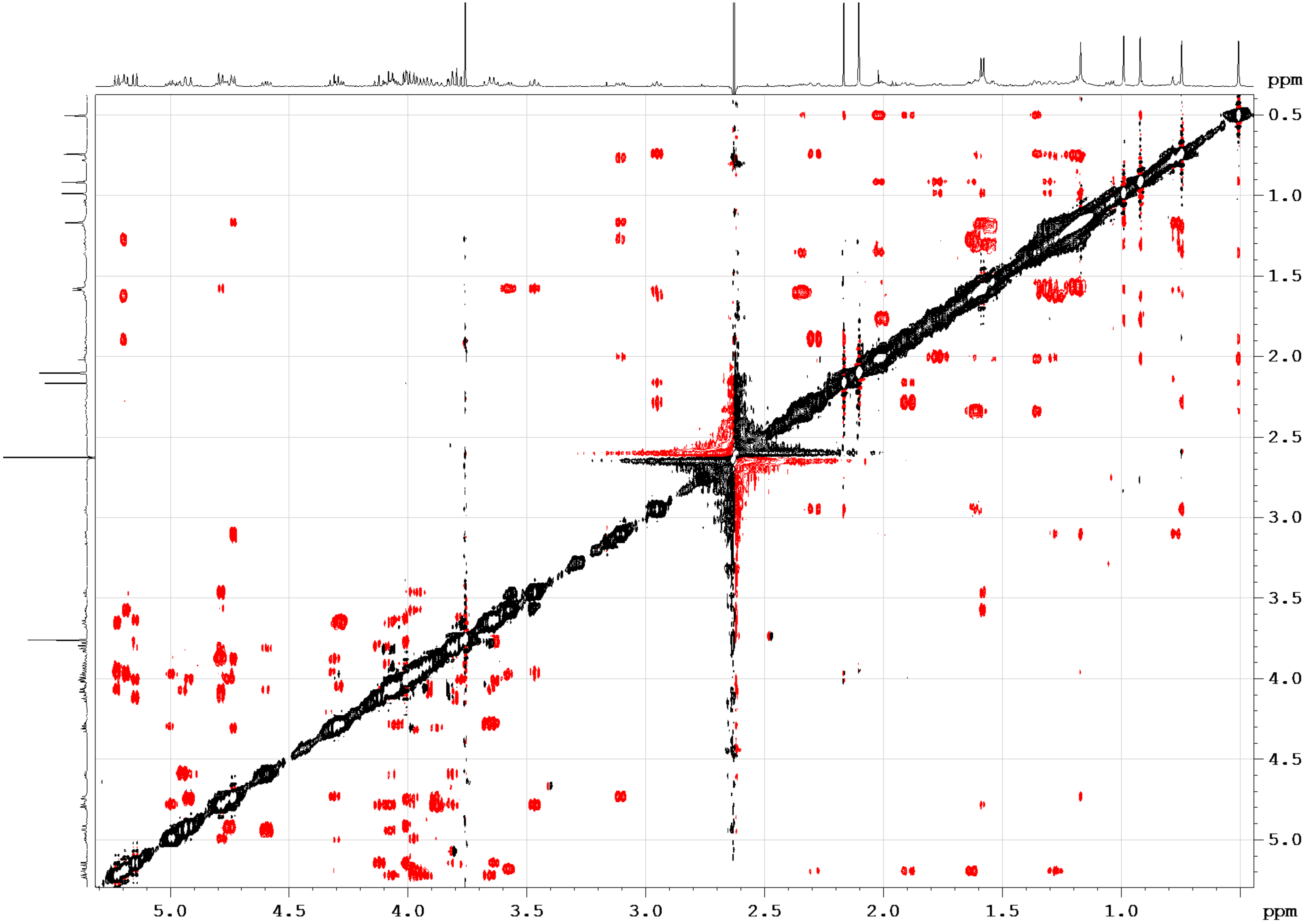

Figure S56. The ROESY (500.12 MHz) spectrum of isokoreoside A (**9**) in C_5D_5N/D_2O (4/1)

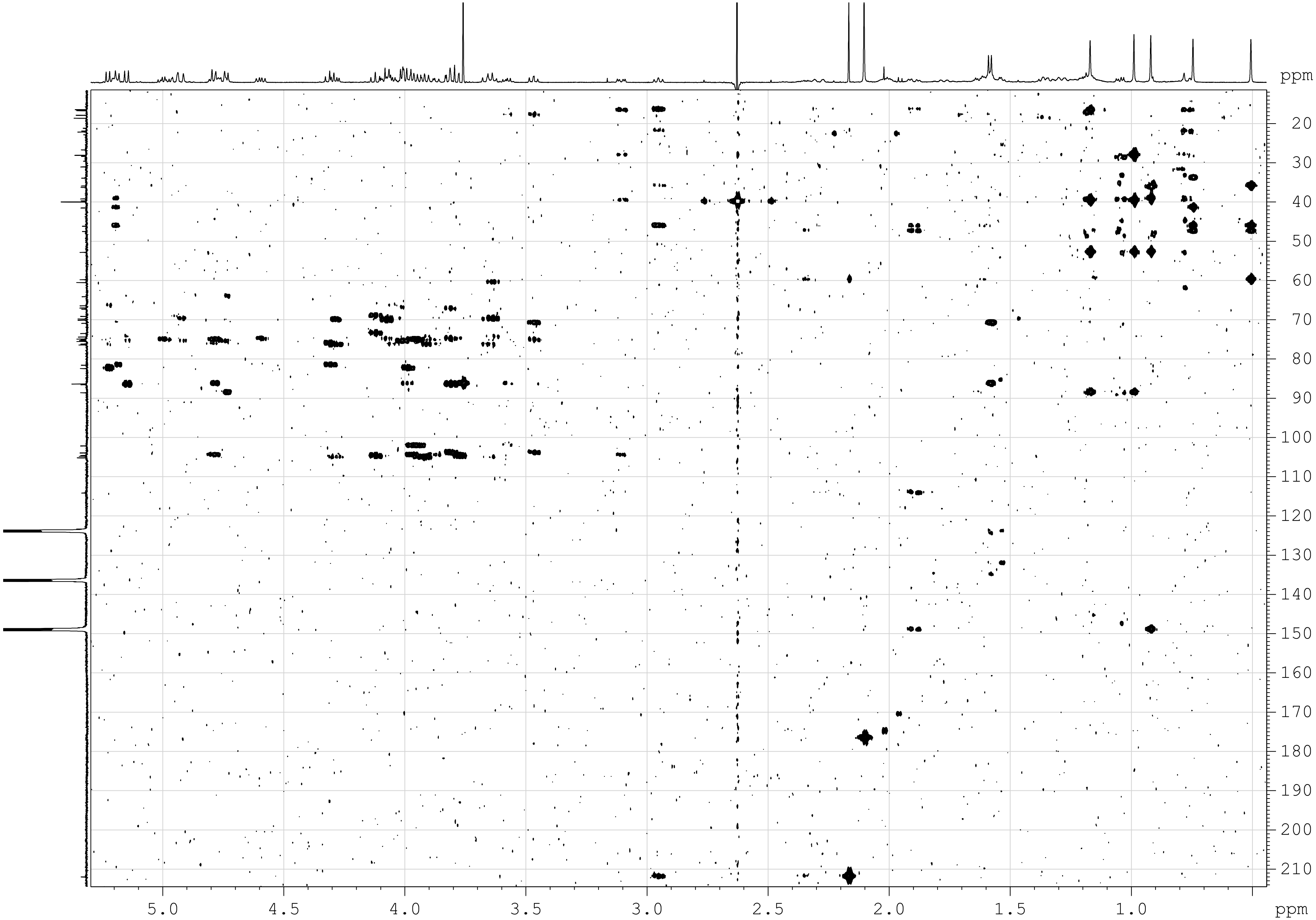

Figure S57. The HMBC (500.12 MHz) spectrum of isokoreoside A (**9**) in C_5D_5N/D_2O (4/1)

Table S9. ^{13}C NMR chemical shifts of koreoside A (**10**).

Position	δ_C mult.[a]	Position	δ_C mult.[a]
1	35.5 CH_2	Qui2 (1→2Xyl1)	
2	26.8 CH_2	1	102.1 CH
3	88.8 CH	2	82.4 CH
4	39.4 C	3	75.2 CH
5	48.7 CH	4	86.3 CH
6	23.1 CH_2	5	70.9 CH
7	122.5 CH	6	17.8 CH_3
8	147.6 C	Glc3 (1→4Qui2)	
9	48.1 CH	1	103.9 CH
10	35.5 C	2	73.4 CH
11	22.4 CH_2	3	86.5 CH
12	33.3 CH_2	4	69.1 CH
13	53.1 C	5	74.8 CH
14	44.9 C	6	67.3 CH_2
15	33.3 CH_2	MeGlc4 (1→3Glc3)	
16	22.3 CH_2	1	104.7 CH
17	61.9 CH	2	74.3 CH
18	24.7 CH_3	3	86.3 CH
19	24.4 CH_3	4	69.8 CH
20	211.7 C	5	75.6 CH
21	30.4 CH_3	6	67.0 CH_2
30	17.3 CH_3	OMe	60.5 CH_3
31	28.7 CH_3	Xyl5 (1→2Qui2)	
32	30.6 CH_3	1	105.2 CH
Xyl1 (1→C-3)		2	74.9 CH
1	104.5 CH	3	76.4 CH
2	81.6 CH	4	70.1 CH
3	75.1 CH	5	66.4 CH_2
4	76.1 CH		
5	64.1 CH_2		

[a] Recorded at 125.67 MHz in C_5D_5N/D_2O.

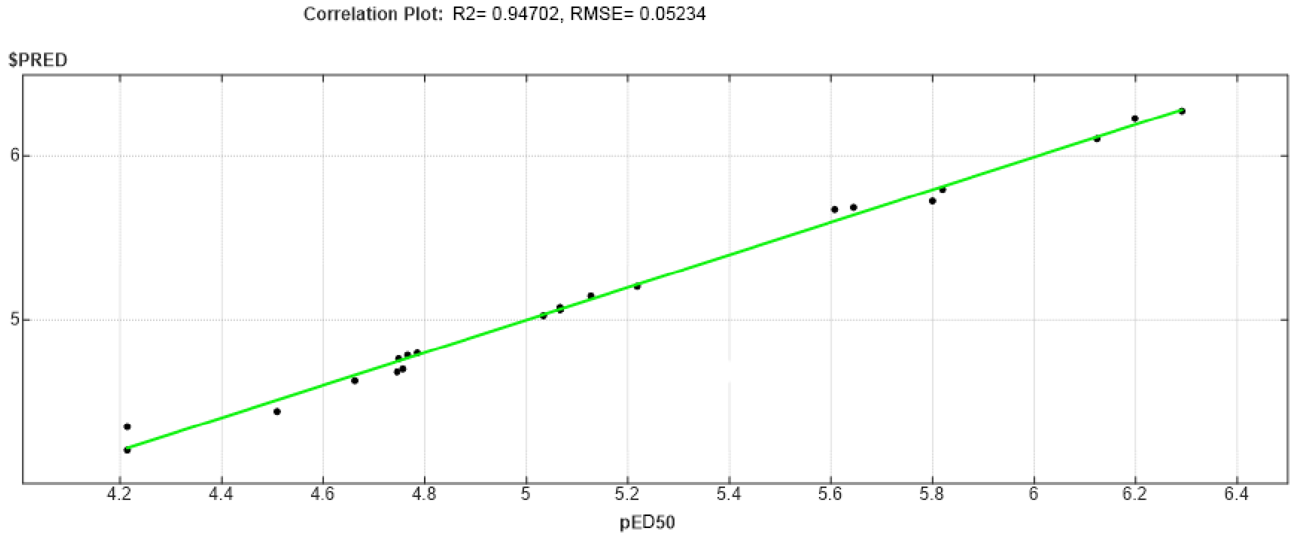

Figure S58. The PCR QSAR model correlation plot reflecting the relationship of predicted and experimental hemolytic activity. The PCR analysis of 148 selected calculated for 20 glycosides tested. The membranotropic action was expressed as pED_{50}.
